# Isotopic evidence for temperate oceans during the Cambrian Explosion

**DOI:** 10.1038/s41598-019-42719-4

**Published:** 2019-04-19

**Authors:** Thomas Wotte, Christian B. Skovsted, Martin J. Whitehouse, Artem Kouchinsky

**Affiliations:** 1 Institut für Geologie, TU Bergakademie Freiberg, Bernhard-von-Cotta-Straße 2, D-09599 Freiberg, Germany; 2 Department of Palaeobiology, Swedish Museum of Natural History, Box 50007, SE-104 05 Stockholm, Sweden; 3 Department of Geosciences, Swedish Museum of Natural History, Box 50007, SE-104 05 Stockholm, Sweden

**Keywords:** Palaeoceanography, Environmental impact, Marine chemistry

## Abstract

The Cambrian Explosion was a key event in the evolution of life on Earth. This event took place at a time when sea surface temperatures have been proposed to reach about 60 °C. Such high temperatures are clearly above the upper thermal limit of 38 °C for modern marine invertebrates and preclude a major biological revolution. To address this dichotomy, we performed *in situ* δ^18^O analyses of Cambrian phosphatic brachiopods via secondary ion mass spectrometry (SIMS). The δ^18^O_phosphate_ data, which are considered to represent the most primary δ^18^O_seawater_ signature, were identified by evaluating the diagenetic alteration of the analyzed shells. Assuming ice-free conditions for the Cambrian ocean and no change in δ^18^O_seawater_ (-1.4‰ to -1‰; V-SMOW) through time, our temperatures vary between 35 °C ± 12 °C and 41 °C ± 12 °C. They are thus clearly above (1) recent subequatorial sea surface temperatures of 27 °C–35 °C and (2) the upper lethal limit of 38 °C of marine organisms. Our new data can therefore be used to infer a minimal depletion in early Cambrian δ^18^O_seawater_ relative to today of about -3‰. With this presumption, our most pristine δ^18^O_phosphate_ values translate into sea surface temperatures of about 30 °C indicating habitable temperatures for subequatorial oceans during the Cambrian Explosion.

## Introduction

**Figure 1:**
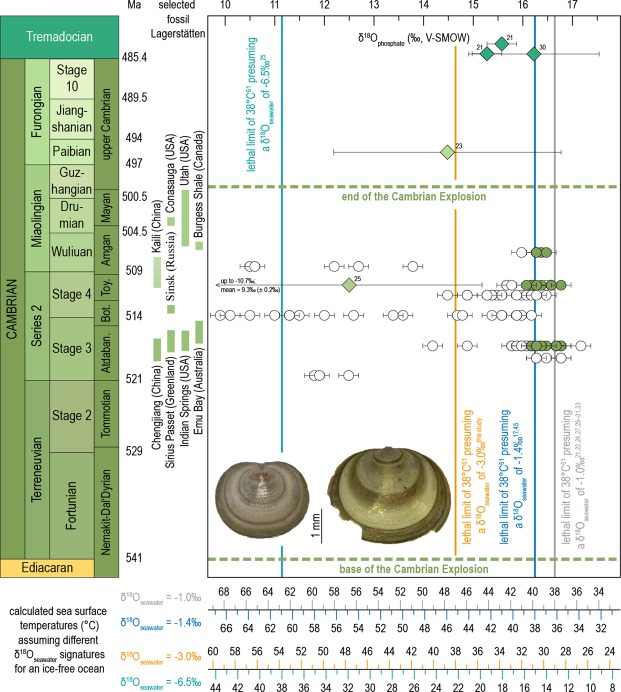
Oxygen isotope compositions of Cambrian and Tremadocian brachiopods and conodonts. Our δ^18^O_phosphate_ data range from 9.9‰ to 17.2‰ (V-SMOW; 1σ ± 0.2‰). Calculated seawater surface temperatures assuming δ^18^O_seawater_ signatures of -1.0‰^21,22,24,27,29–31,33^ and -1.4‰^17,45^ vary between 34 °C ± 12 °C and 68 °C ± 11 °C. Excluding altered and probably altered δ^18^O_phosphate_ data, our calculated sea surface temperatures range from 35 °C ± 12 °C to 41 °C ± 12 °C. In contrast, assuming a Cambrian δ^18^O_seawater_ signature of -6.5‰^25^, reconstructed sea surface temperatures are incredible low, ranging from 12 °C ± 14 °C to 16 °C ± 14 °C. Our new data therefore clearly support the hypothesis of a secular change in the ocean δ^18^O during Earth history. A minimal depletion in early Cambrian δ^18^O_seawater_ relative to today of about -3‰ is assumed. With this presumption, our most pristine δ^18^O_phosphate_ values translate into sea surface temperatures of 28 °C ± 13 °C to 32 °C ± 13 °C. Our most pristine δ^18^O_phosphate_ values are illustrated by green circles, (probably) altered data by white circles. Additional data points of the Cambrian Series 2–Tremadocian were generated from bulk sample analyses of conodonts and brachiopods^23,30^ and *in situ* measurements of conodonts^21^ and brachiopods^25^. SIMS data of ref.^25^ vary between -10.6‰ (±0.1‰) and 14.8‰ (±0.4‰); mean = 9.3‰ (±0.2‰). Global Cambrian time scale and selected fossil Lagerstätten are from ref.^38,43^. Siberian nomenclature from ref.^41^. Atdaban. = Atdabanian Stage, Bot. = Botoman Stage, Toy. = Toyonian Stage.

The Cambrian Explosion was one of the most important events in the history of life on Earth. Within a few million years, the simple life of the Precambrian evolved to highly organized organisms with modern anatomical characteristics such as a chorda dorsalis, complex eyes and legs, and biomineralized skeletal parts^1–5^. During this fundamental biotic event, the metazoans invaded all kinds of marine habitats and evolved complex ecosystems and food webs for the first time^6,7^. Insights into this often odd-looking Cambrian world are provided by a variety of global fossil Lagerstätten such as the Burgess Shale (Canada)^8^, Chengjiang (South China)^9^, or Sirius Passet (Greenland)^10^ (Fig. 1). The reasons for this radical faunal turnover are still poorly understood but changes in ocean chemistry^11,12^, the rise in atmospheric and oceanic oxygen concentration to modern levels^13,14^, and the presumed evolutionary arms race between predators and prey^15,16^ have been discussed as major driving mechanisms. Additionally, the establishment of habitable seawater temperatures seems to be most essential for the evolution of diverse marine metazoan life. Information on marine paleotemperatures is inferred from studies of oxygen isotopes (δ^18^O) from bulk carbonate samples, marine cements, cherts, phosphorites, or fossils of calcitic or phosphatic composition. However, independent of the material analyzed, these oxygen isotope ratios become progressively depleted in the heavier ^18^O towards older stratigraphic units^17–20^, resulting in a rising trend of reconstructed seawater temperatures with increasing age. For the Cambrian, temperatures of about 60 °C or even higher have been proposed^17–23^, which is intolerable for most animals in modern oceans. Whether this trend reflects (1) an increased diagenetic alteration of older rocks, (2) secular changes in the oxygen isotopic composition of ancient ocean seawater, or (3) higher original seawater temperatures in the past, is still controversial^17–23^. If seawater oxygen isotope composition is considered as constant throughout Earth history, a δ^18^O value of ~ -1‰ is generally assumed to characterize an ice-free planet^24^. In contrast, assuming that seawater δ^18^O changed substantially over time, a global average of Cambrian seawater δ^18^O of up to -6.5‰ or even -8‰ has been proposed^25^. These presumptions would shift the reconstructed temperature of ancient seawater towards higher or lower values, respectively.

Oxygen isotopes of marine carbonates (δ^18^O_carbonate_) have been used for studies of seawater temperatures since the beginning of geochemical research. One drawback of carbonate mineralogy is its susceptibility to diagenetic alteration, making the application of δ^18^O_carbonate_ more doubtful for older stratigraphic successions which are typically characterized by intense alteration and recrystallization. The analysis of phosphate minerals (i.e. apatite), which are considered to be more resistant to diagenetic processes than carbonate, are therefore an attractive alternative, or at least a complementary tool, for assessing paleoclimate conditions^18,26,27^. In Paleozoic successions, conodonts and phosphatic brachiopods have been used in addition to calcite-shelled brachiopods to provide information about the seawater temperatures via their oxygen isotope composition with high stratigraphic resolution^27–29^. Seawater temperatures calculated from δ^18^O_phosphate_ vary between 36 and 53 °C (or even 62 °C) for the late Cambrian (brachiopods)^23^ and between 33 and 41 °C for the Early Ordovician (Tremadocian; brachiopods and conodonts)^30^, in both cases assuming an ice-free ocean and no significant change in δ^18^O_seawater_ over time. However, the traditional δ^18^O method of analysis of phosphates requires bulk samples of about 0.5–1 mg^29,31^ which equates to several hundred conodont elements or several complete brachiopod shells depending on their size and weight. The pooling of various specimens may result in significant problems. Even if carefully evaluated, sampling of altered material cannot be avoided with certainty as these areas are not observable macroscopically. Incorporation of only a fraction of altered sample material will result in δ^18^O_phosphate_ data that do not represent the primary oxygen isotopic composition of ambient seawater. A possible alternative may be the analysis of clumped isotopes, which could allow the discrimination of the diagenetic isotopic signal from the primary one^32^. A critical drawback of this method, however, is the enormous sample size of up to 12 mg and 200 mg required for calcite and phosphate samples, respectively^32^. Against this background, *in situ* measurements of oxygen isotopes have been applied using SIMS and similar high precision *in situ* analyses^21,25,33–37^, which minimize sample sizes and therefore the risk of contamination of the primary oxygen isotope signal. Again, assuming ice-free conditions and no significant variation in seawater δ^18^O over time, the corresponding values of Ordovician δ^18^O_conodont_ in such analyses range from 15.3‰ to 19.6‰ (V-SMOW)^21^. Temperatures calculated from these values vary from 25 °C to 44 °C^21^. Even higher calculated temperatures are reported from middle Cambrian brachiopods with a mean value of 71 °C ± 11 °C and a minimum temperature of 46 °C ± 10 °C^25^. However, assuming a Cambrian δ^18^O seawater composition of -6.5‰ these Cambrian temperatures were re-calculated to a minimum seawater temperature of 22 °C ± 10 °C (mean value of 47 °C ± 12 °C) and interpreted to represent the original seawater temperature of early–middle Cambrian southern latitudes (65°S to 70°S)^25^.

Here we present a study of *in situ* oxygen isotope analyses of Cambrian phosphatic brachiopods using the SIMS technique (CAMECA ims1280 at the Nordsim laboratory, Swedish Museum of Natural History, Stockholm), in order to shed new light on the unexpected high seawater temperatures estimated for the Cambrian Period and their aforementioned implications.

## Stratigraphic Setting and Material

**Figure 2:**
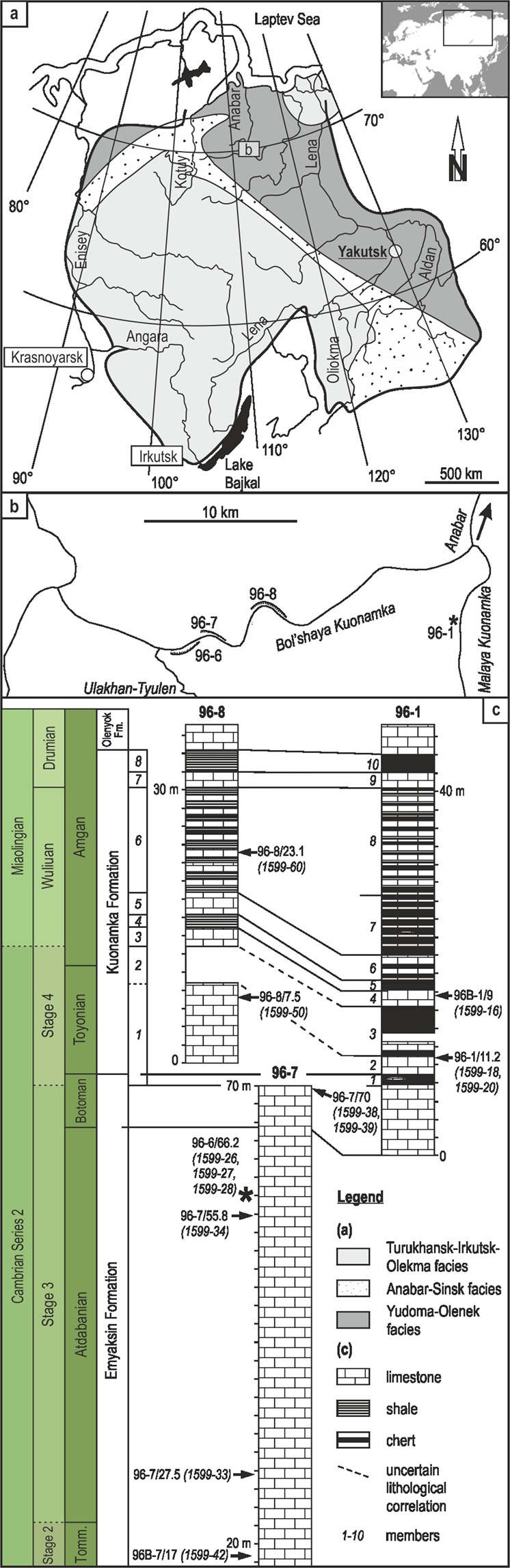
Geology of the sample area and stratigraphy of the samples. (**a**) Geological map of the northern Siberian Platform. (**b**) Geographic positions of the sections investigated. Section 96-1 is located on the Malaya Kuonamka River, sections 96-6, 96-7, and 96-8 are located on the Bol’shaya Kuonamka River. (**c**) Stratigraphic columns of the sections investigated. Derivation of brachiopod samples analyzed herein are marked by arrows with numbers. Tomm. = Tommotian Stage, Fm. = Formation.

Thirteen exceptionally conserved Cambrian lingulid and acrotetid brachiopod shells (Fig. 1; Supplementary Material) were analyzed for their oxygen isotopic composition. The shells are composed of fluorapatite. They come from the Siberian Platform which is characterized by an almost horizontally bedded Cambrian sedimentary succession showing no or only minor metamorphic or tectonic overprint. Within the sedimentary rocks covering the Terreneuvian–Miaolingian^38^ interval, three distinct facies realms are developed, characterizing an eastward deepening of the depositional environment^39,40^. From west to east these are the restricted–lagoonal Turukhansk-Irkutsk-Olekma, the open marine Yudoma-Olenek, and the transitional Anabar-Sinsk facies realms (Fig. 2). The thirteen brachiopods analyzed herein belong to the open marine Yudoma-Olenek facies realm. Sections investigated are located along the Malaya Kuonamka (sections 96-1 and 96B-1) and Bol’shaya Kuonamka (sections 96-7, 96B-7, and 96-8) rivers on the eastern flank of the Anabar Uplift of the Siberian Platform (Fig. 2). The fauna from the sections includes trilobites, brachiopods, echinoderms, and other shelly fossils^41^ typical for shallow marine Cambrian carbonate environments with normal salinity.

Samples cover the Tommotian–Amgan interval (according to the Siberian nomenclature^41,42^) respectively the Terreneuvian (Cambrian Stage 2)–Miaolingian (Wuliuan Stage) interval (according to the international nomenclature^38,43^; Figs 1 and 2), and thus offer a unique window into the time of the Cambrian Explosion.

## Results

### Calculation of seawater temperatures

Ninety-six spots on 13 brachiopod shells were analyzed for their oxygen isotopic composition. The δ^18^O_phosphate_ values obtained range from 9.9‰ to 17.2‰ (V-SMOW; 1σ ± 0.2‰). Oxygen isotope data vary between specimens, but also show intra-sample variations of about 3‰ up to 6‰ (samples 1599-16, 1599-38, 1599-39; see Supplementary Table 1). For calculation of paleotemperature we applied the equation of Lécuyer and co-authors^44^. The major part of the Terreneuvian–Miaolingian interval is considered as an ice-free period. Assuming (1) an ice-free Cambrian ocean and therefore a δ^18^O_seawater_ value of -1.0‰^21,22,24,27,29–31,33^ or -1.4‰^17,45^, and (2) considering error propagation, our reconstructed sea surface temperatures vary between 34 °C ± 12 °C and 68 °C ± 11 °C, (Fig. 1, and see Supplementary Material for additional information). In contrast, presuming an increasing δ^18^O_seawater_ over time and a global Cambrian ocean average δ^18^O of -6.5‰^25^, calculated temperatures become incredible cooler, ranging from 11 °C ± 14 °C to 44 °C ± 12 °C (Fig. 1).

### Diagenetic constraints

It is generally considered, that phosphate minerals are less susceptible to diagenetic alteration than carbonates. However, several studies indicate that skeletal phosphate minerals are metastable and were typically recrystallized during early diagenesis^17,46,47^. Whereas no widespread diagenetic processes are known to result in an enrichment of ^18^O in phosphates^17^, more negative δ^18^O values are generally considered as a sign of diagenetic alteration^27^.

**Figure 3:**
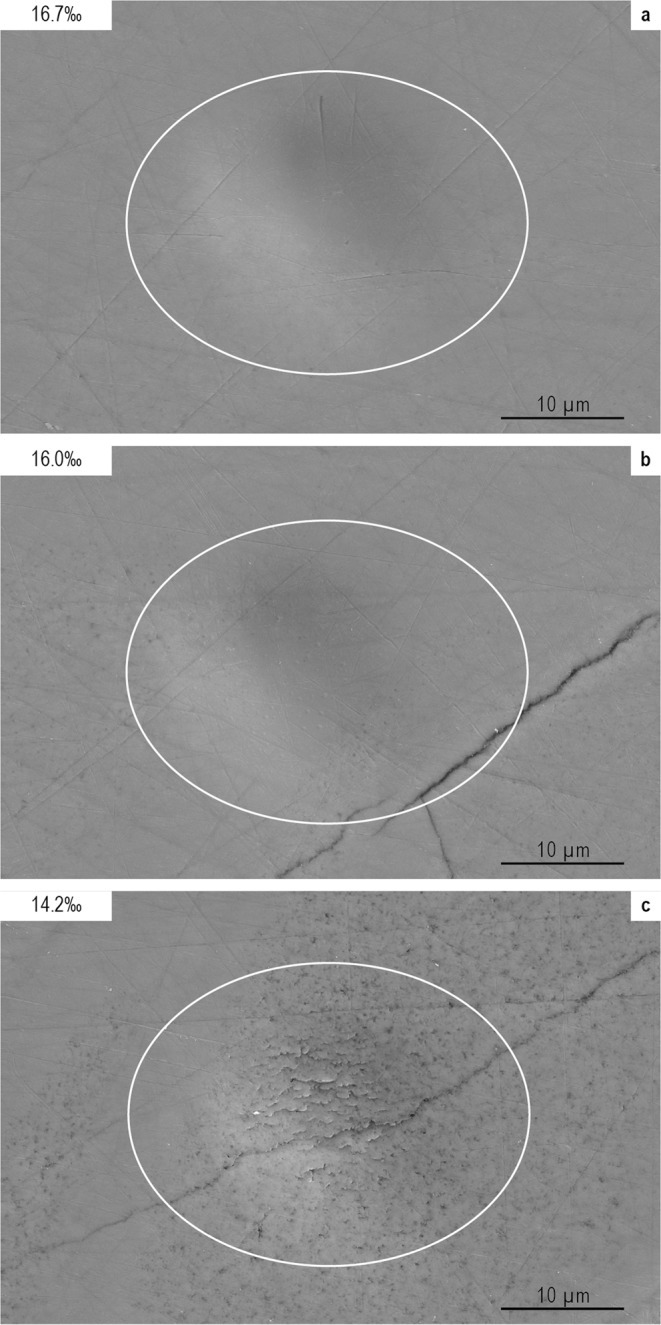
SEM images of selected spots from sample 1599-28 showing different stages of alteration. (**a**) Dense material (e.g., spot 1; 16.7‰) without any indication for recrystallization processes is generally characterized by more positive δ^18^O_phosphate_ values. We interpret such parts as representing the most primary δ^18^O_seawater_ signature. (**b**) Slightly more negative δ^18^O_phosphate_ values (e.g., spot 3; 16.0‰) most probably mirror diagenetic alteration caused by pores, fractures, or laminae acting as pathways for fluid migration. (**c**) Fractures and recrystallized shell material correspond to a considerably lower δ^18^O_phosphate_ values (spot 6; 14.2%), thus appear to represent diagenetically altered shell portions.

In our samples, reflected light optical images already indicate recrystallization of individual shells and shell portions, which are characterized by considerably darker color and inhomogeneous appearance (Supplementary Material). SEM photographs confirm this first order identification by revealing the brachiopod shell ultrastructure in detail (Fig. 3; Supplementary Material). Dense crystalline shell material without any indication for recrystallization and alteration is characterized by δ^18^O_phosphate_ values generally more positive than 16.0‰, which thus appear to represent the most primary δ^18^O_seawater_ signature. Pores, laminae, and secondary fissures in other shell portions potentially act as pathways for fluid migration and therewith for diagenetic alteration. The visually identified recrystallized shells or portions of shells often, if not ubiquitously, yield lower δ^18^O_phosphate_ values, and are thus interpreted as most probably diagenetically altered (see Fig. 1 and Supplementary Material). Even for spots showing only initial recrystallization or partially pores, diagenetic alteration cannot be excluded with certainty. All these (probably) altered data are not included in our final discussion. The co-occurrence of both, dense crystalline and recrystallized areas in one and the same shell also explains large intra-shell variations as identified in samples 1599-16, 1599-38, or 1599-39.

### Analytical constrains

Despite the obvious advantages of δ^18^O analyses on biogenic apatite using SIMS (e.g., analyses of very small samples or distinct shell portions), a number of sources for probable errors have to be addressed, not all of which are presently fully understood. The general chemical formula of apatite Ca_5_(PO_4_, CO_3_)_3_(CO_3_, F, OH) contains three common oxygen bearing molecular groups. Whereas δ^18^O_phosphate_ analyses performed by the conventional IRMS method only isolate the PO_4_^3−^ group as trisilverphosphate (Ag_3_PO_4_) and subsequently analyze the oxygen isotopes by high-temperature reduction^27,29,31,37,48^, the SIMS technique indiscriminately samples oxygen from PO_4_^3−^, CO_3_^2−^, OH^−^, as well as residual organics^36,49^. The integration of oxygen from these different sources will likely influence the SIMS-determined δ^18^O_phosphate_ values. This problem is relevant when analyzing e.g. organo-phosphatic brachiopods characterized by an alternation of carbonate-fluorapatite and organic-rich laminae. Studies on recent and fossil lingulid brachiopods have documented intra-shell δ^18^O_phosphate_ variations often exceeding 4‰ that probably represent vital fractionation effects^50^. Similar intra-element δ^18^O_phosphate_ variations were detected from conodonts^36^. Therefore, care should be taken when interpreting δ^18^O_phosphate_ values generated by SIMS. However, variations in δ^18^O_phosphate_ can be minimized by high spatial resolution SIMS analyses on the most pristine areas^36^.

Multitude of paired SIMS and IRMS δ^18^O_phosphate_ analyses on conodonts have shown, that SIMS normally yields values 0.5–1.0‰ higher^21,33,35,36^. There is still uncertainty about the systematic offset between both methods in measuring the δ^18^O_phosphate_ values in phosphate brachiopods. This unknown offset would of course bias the calculation of paleotemperatures to higher values. In this context it should be also mentioned, that coefficients in the available thermometer equations^44,51,52^ were determined by IRMS (isotope ratio mass spectrometer) analyses. Whether these equations have to be adjusted for treatment of SIMS data, needs further investigation.

A probable further weakness arises from the Durango apatite standard which is commonly used to calibrate the δ^18^O_phosphate_ measurements generated by SIMS. Crystals of Durango apatite are generally assumed to be homogeneous in δ^18^O^53,54^. A recent study, however, questions this presumption and identifies conspicuous intra-crystalline heterogeneity in δ^18^O in all apatite standards available^53^. However, δ^18^O analyses of our samples and in house Durango apatite were performed on restricted crystal portions and δ^18^O_Durango_ values show no significant variation and a reproducibility generally better than ±0.2‰ (1σ).

## Discussion

### Biological and paleogeographical considerations

**Figure 4:**
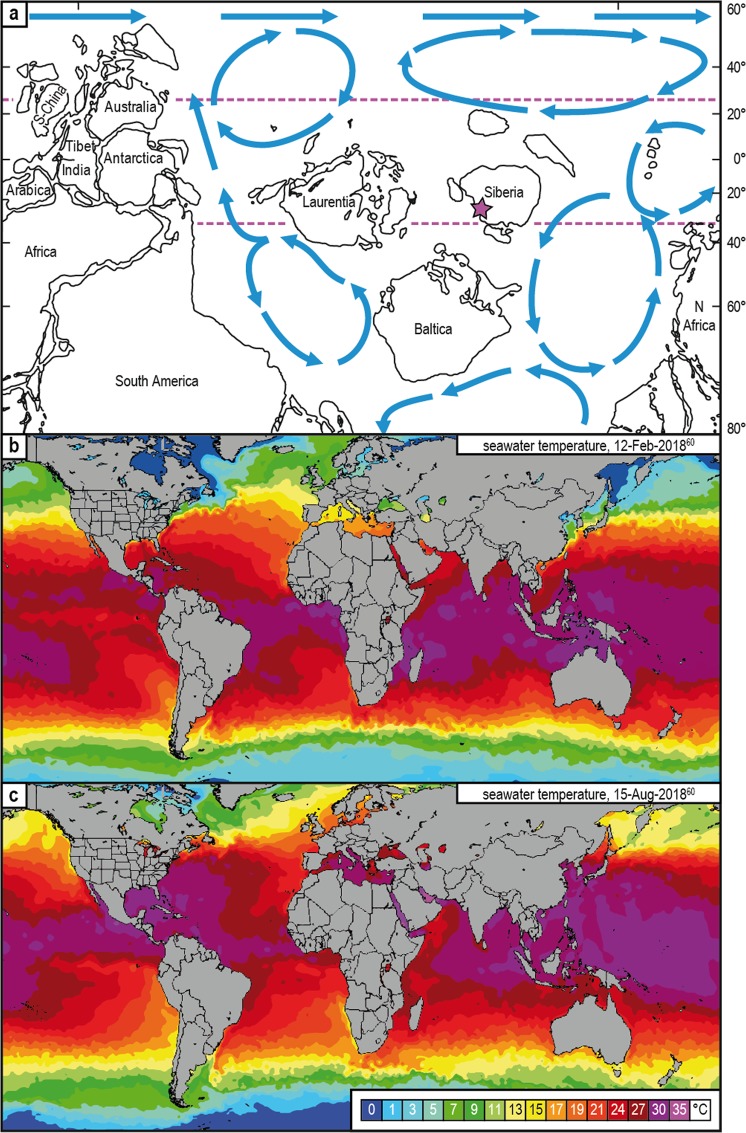
Reconstruction of the Terreneuvian earth and illustration of the recent seawater temperature. (**a**) Reconstruction of lower Cambrian paleogeography and ocean currents (blue arrows). Dotted purple lines correspond with the 35 °C isotherms of ref.^25^. The location of the sections investigated has been identified (purple star). (**b,c**) Illustration of the current seawater temperatures during austral summer (**b**)/winter (**c**) is based on the open source ref.^60^. Recent subequatorial seawater temperatures vary between 24 °C and 27 °C (austral winter) and between 27 °C and 35 °C (austral summer). Comparable mean annual temperatures could be also assumed for the Siberian carbonate shelf, located in a similar paleogeographic position during the Terreneuvian–Cambrian Series 2 interval.

During the early and middle Cambrian, Siberia was located in a subtropical position south of the equator^55^ (Fig. 4a). Most recent simulations indicate mean sea surface temperatures of more than 35 °C for this paleogeographic region with minimum and maximum values of about 33 °C (austral winter) and 40 °C (austral summer), respectively^25^. Similar data were modeled for Furongian to the middle Silurian equatorial to subequatorial oceans, with values varying between 30 and 35 °C^56^. Comparable warm temperate ocean conditions of about 30 °C with maxima up to 37 °C (or even approaching 40 °C) are not unusual in earth history. They have also been estimated for the Triassic, Middle Jurassic, and Cretaceous times^57–59^.

Average temperatures of present subequatorial ocean areas of comparable latitude to the position of Siberia in the early Cambrian vary between 24 °C (austral winter) and 35 °C (austral summer), respectively^60^ (e.g., eastern Indian ocean, Java-, Banda-, Timor-, and Arafura seas; Fig. 4b,c). Assuming an ice-free Cambrian ocean and a δ^18^O_seawater_ value of -1.0‰^21,22,24,27,29–31,33^ or -1.4‰^17,45^, our temperatures calculated from the most primary δ^18^O_phosphate_ values vary between 35 °C ± 12 °C and 41 °C ± 12 °C. They are thus clearly higher than modern annual subequatorial temperatures. They also often exceed the upper lethal temperature for modern marine species which is typically considered to be 38 °C with habitat-dependent fluctuations towards higher and lower values^61–64^. The biological tolerance limit of 38 °C can be probably applied to Cambrian life forms, even if modern restrictions may not be strictly assumed for deep geological time.

### Implications for the interpretation of the Paleozoic seawater temperature

Considering the thermal limit of 38 °C as a critical threshold, secular changes in the oxygen isotopic composition of ocean seawater are necessary to explain the presence of marine life in the Paleozoic. Based on our interpretation of the Siberian data a minimal depletion in oxygen isotopic composition of early–middle Cambrian seawater relative to today would be about -3‰. With this assumption and excluding altered and probably altered δ^18^O_phosphate_ values, calculated subtropical sea surface temperatures vary between 28 °C ± 13 °C and 32 °C ± 13 °C (Fig. 1). The minimum temperature becomes even lower (26 °C ± 13 °C) if we involve the most positive δ^18^O_phosphate_ value (17.2‰) into our calculation. These sea surface temperatures would be (1) clearly below the upper lethal limit of 38 °C of marine organisms (Fig. 1) and (2) comparable to temperatures of present subequatorial ocean areas (Fig. 4). Unexpected high temperatures calculated from the *in situ* measurements and bulk sample analyses of brachiopods^23,25^ for the Cambrian are thus most probably an artifact of diagenetic alteration. However, the possibility of larger ^18^O depletions up to -6.5‰ or even -8‰^25^ cannot be excluded completely if (1) the observed spread of data in our samples represent more than just an artifact of alteration and/or (2) other thermometer equations (e.g., Pucéat and co-authors^52^) are applied for temperature calculation.

## Methods

### Oxygen isotope analyses

For oxygen isotope (δ^18^O) analyses, brachiopod shells were mounted centrally on two-side tape fixed on a 4.5 × 4.5 cm acrylic glass (Supplementary Material). Six grains of an in house Durango apatite standard were added and both (samples and standard) were embedded in an epoxy pellet of 2.5 cm in diameter. After hardening of about 24 hours, pellets were removed from the tape and polished to a low relief in order to minimize analytical artifacts^65,66^. To facilitate navigation during SIMS analyses, a photograph image of the stub was generated using the Olympus cellSens Standard software (Supplementary Material). Prior to measurements, samples were coated with 30 nm of gold.

δ^18^O analyses were performed on a CAMECA ims1280 large-geometry ion microprobe at the Department of Geosciences of the Swedish Museum of Natural History (Nordsim facility). For analyses, a ^133^Cs^+^ ion beam with an intensity of ~3 nA was critically focused and a small raster applied to homogenize the beam profile on the sample, resulting in an analyzed volume of about 10 µm width and about 2 µm depth. For calibration, two–four analyses of the Durango apatite were performed before and after six analyses of brachiopod shells. Results are reported in ‰ relative to the V-SMOW (Vienna Standard Mean Ocean Water) standard with reproducibility generally better than ± 0.2‰ (1σ). A total of 134 measurements (96 samples and 38 standard) was performed (Supplementary Material & Supplementary Table 1). Parallel to oxygen isotope measurements each analyzing spot and surrounding shell material was imaged.

### Scanning electron microscopy and energy-dispersive X-ray spectroscopy

Scanning electron microscopy (SEM) was performed on the Zeiss Sigma 300 VP at the Department of Paleontology of the University of Cologne. We used the gold-coated stubs already utilized for δ^18^O analyses. In addition to the SEM images, energy-dispersive X-ray spectroscopy (EDS or EDXS) was applied using a X-Max^N^ 80 Silicon Drift Detector (Oxford Instruments) connected to the SEM. No identification of inconsistencies in element concentration could be shown by the SEM-EDS analyses (see Supplementary Material for selected spots).

All data generated or analyzed during this study are included in this published article (and its Supplementary Material).

## Supplementary information

Supplementary Information
