# Supplementary material for: Isotopic evidence for temperate oceans during the Cambrian Explosion
Source: Sci Rep. 2019 Apr 19;9:6330. doi: 10.1038/s41598-019-42719-4 (PMC6474879; doi:10.1038/s41598-019-42719-4)
Supplement: Supplementary file 1 — Supplementary Information [file 41598_2019_42719_MOESM1_ESM.pdf]

Isotopic evidence for tempered oceans during the Cambrian Explosion

Thomas Wotte[1*], Christian B. Skovsted[2], Martin J. Whitehouse[3], Artem Kouchinsky[2]

[1]Institut für Geologie, TU Bergakademie Freiberg, Bernhard-von-Cotta-Straße 2, D-09599 Freiberg, Germany (thomas.wotte@geo.tu-freiberg.de)

[2]Department of Palaeobiology, Swedish Museum of Natural History, Box 50007, SE-104 05 Stockholm, Sweden

[3]Department of Geosciences, Swedish Museum of Natural History, Box 50007, SE-104 05 Stockholm, Sweden

Supplementary Table 1. $\delta^{18}O_{phosphate}$ values from samples and the Durango apatite standard with reproducibility generally better than ± 0.2‰ (1 σ), Sea surface temperatures (Temp.) were calculated using the equation of Lécuyer and co-authors[44] assuming different $\delta^{18}O_{seawater}$ signatures

Sample ID	Sample ID in the sections	Stratigraphy	$\delta^{18}O_{phosphate}$ (‰, V-SMOW)	± ‰ (1 σ)	$\delta^{18}O_{seawater}$ = -1.0‰		$\delta^{18}O_{seawater}$ = -1.4‰		$\delta^{18}O_{seawater}$ = -3.0‰		$\delta^{18}O_{seawater}$ = -6.5‰		Diagenetic alteration
					Temp. (°C)	± °C	Temp. (°C)	± °C	Temp. (°C)	± °C	Temp. (°C)	± °C	
Dur_mt1599_@044			9.1	0.2									
Dur_mt1599_@045			9.4	0.2									
Dur_mt1599_@046			9.4	0.2									
Dur_mt1599_@047			9.3	0.2									
1599-16-01	96B-1/9	Amgan	13.8	0.2	51	11	49	12	42	12	26	13	yes
1599-16-02	96B-1/9	Amgan	12.7	0.2	56	11	54	11	47	12	31	13	yes
1599-16-03	96B-1/9	Amgan	12.2	0.2	58	11	56	11	49	12	33	12	yes
1599-16-04	96B-1/9	Amgan	10.5	0.2	66	11	64	11	57	11	41	12	yes
1599-16-05	96B-1/9	Amgan	10.6	0.2	65	11	63	11	56	11	40	12	yes
1599-18-01	96-1/11.2	Toyonian	14.9	0.2	46	12	44	12	37	12	21	13	yes
Dur_mt1599_@048			9.2	0.2									
Dur_mt1599_@049			9.0	0.2									
1599-18-02	96-1/11.2	Toyonian	15.5	0.2	43	12	41	12	34	12	18	13	yes
1599-18-03	96-1/11.2	Toyonian	15.4	0.2	44	12	42	12	35	12	19	13	yes
1599-18-04	96-1/11.2	Toyonian	14.9	0.2	46	12	44	12	37	12	21	13	yes
1599-18-05	96-1/11.2	Toyonian	14.5	0.2	47	12	46	12	38	12	23	13	yes
1599-18-06	96-1/11.2	Toyonian	15.6	0.2	42	12	41	12	33	12	18	13	yes
1599-18-07	96-1/11.2	Toyonian	15.4	0.2	44	12	42	12	35	12	19	13	yes
Dur_mt1599_@050			8.9	0.2									
Dur_mt1599_@051			8.9	0.2									
1599-20-01	96-1/11.2	Toyonian	15.3	0.2	44	12	42	12	35	12	19	13	yes
1599-20-02	96-1/11.2	Toyonian	16.2	0.2	40	12	38	12	31	13	15	14	probably
1599-20-03	96-1/11.2	Toyonian	16.3	0.2	39	12	38	12	30	13	15	14	probably

1599-20-04	96-1/11.2	Toyonian	16.1	0.2	41	12	39	12	32	13	16	14	probably
1599-20-05	96-1/11.2	Toyonian	16.5	0.2	38	12	37	12	29	13	14	14	probably
1599-20-06	96-1/11.2	Toyonian	16.0	0.2	41	12	39	12	32	13	16	14	probably
Dur_mt1599_@052			9.2	0.2									
Dur_mt1599_@053			9.2	0.2									
1599-20-07	96-1/11.2	Toyonian	15.9	0.2	41	12	40	12	32	13	17	14	probably
1599-26-01	96-6/66.2	Atdabanian	17.2	0.2	35	12	34	12	26	13	11	14	probably
1599-26-02	96-6/66.2	Atdabanian	16.7	0.2	38	12	36	12	29	13	13	14	probably
1599-26-03	96-6/66.2	Atdabanian	15.8	0.2	42	12	40	12	33	13	17	14	probably
1599-26-04	96-6/66.2	Atdabanian	16.2	0.2	40	12	38	12	31	13	15	14	probably
1599-26-05	96-6/66.2	Atdabanian	16.4	0.2	39	12	37	12	30	13	14	14	no
Dur_mt1599_@055			9.2	0.2									
Dur_mt1599_@056			8.9	0.2									
1599-26-06	96-6/66.2	Atdabanian	16.3	0.2	39	12	38	12	30	13	15	14	no
1599-26-07	96-6/66.2	Atdabanian	16.2	0.2	40	12	38	12	31	13	15	14	no
1599-26-08	96-6/66.2	Atdabanian	16.1	0.2	40	12	39	12	31	13	16	14	no
1599-26-09	96-6/66.2	Atdabanian	16.0	0.2	41	12	39	12	32	13	16	14	no
1599-27-01	96-6/66.2	Atdabanian	16.8	0.2	37	12	35	12	28	13	12	14	no
1599-27-02	96-6/66.2	Atdabanian	16.3	0.2	40	12	38	12	31	13	15	14	no
Dur_mt1599_@057			8.8	0.2									
Dur_mt1599_@058			9.1	0.2									
1599-27-03	96-6/66.2	Atdabanian	16.7	0.2	38	12	36	12	29	13	13	14	no
1599-27-04	96-6/66.2	Atdabanian	16.1	0.2	41	12	39	12	32	13	16	14	probably
1599-27-05	96-6/66.2	Atdabanian	15.8	0.2	42	12	40	12	33	13	17	14	probably
1599-27-06	96-6/66.2	Atdabanian	15.9	0.2	41	12	39	12	32	13	17	14	probably
1599-27-07	96-6/66.2	Atdabanian	16.8	0.2	37	12	36	12	28	13	13	14	probably
1599-28-01	96-6/66.2	Atdabanian	16.7	0.2	38	12	36	12	29	13	13	14	no
Dur_mt1599_@059			8.9	0.2									
Dur_mt1599_@060			9.1	0.2									
1599-28-02	96-6/66.2	Atdabanian	16.3	0.2	39	12	38	12	30	13	15	14	no
1599-28-03	96-6/66.2	Atdabanian	16.0	0.2	41	12	39	12	32	13	16	14	probably
1599-28-04	96-6/66.2	Atdabanian	16.2	0.2	40	12	38	12	31	13	15	14	no

1599-28-05	96-6/66.2	Atdabanian	14.9	0.2	46	12	44	12	37	12	21	13	yes
1599-28-06	96-6/66.2	Atdabanian	14.2	0.2	49	12	47	12	40	12	24	13	yes
1599-28-07	96-6/66.2	Atdabanian	16.2	0.2	40	12	38	12	31	13	15	14	no
Dur_mt1599_@061			8.8	0.2									
Dur_mt1599_@063			9.1	0.2									
1599-28-08	96-6/66.2	Atdabanian	16.2	0.2	40	12	38	12	31	13	15	14	no
1599-28-09	96-6/66.2	Atdabanian	16.4	0.2	39	12	37	12	30	13	15	14	no
1599-28-10	96-6/66.2	Atdabanian	16.0	0.2	41	12	39	12	32	13	16	14	probably
1599-28-11	96-6/66.2	Atdabanian	16.5	0.2	38	12	37	12	29	13	14	14	no
1599-28-12	96-6/66.2	Atdabanian	16.7	0.2	38	12	36	12	29	13	13	14	no
1599-33-01	96-7/27.5	Atdabanian	11.8	0.2	60	11	58	11	51	11	35	12	yes
Dur_mt1599_@064			8.9	0.2									
Dur_mt1599_@065			8.9	0.2									
1599-33-02	96-7/27.5	Atdabanian	11.9	0.2	59	11	58	11	50	11	35	12	yes
1599-33-03	96-7/27.5	Atdabanian	12.5	0.2	56	11	55	11	47	12	32	13	yes
1599-34-01	96-7/55.8	Atdabanian	16.8	0.2	37	12	36	12	28	13	13	14	probably
1599-34-02	96-7/55.8	Atdabanian	16.8	0.2	37	12	35	12	28	13	12	14	probably
1599-34-03	96-7/55.8	Atdabanian	16.3	0.2	40	12	38	12	31	13	15	14	probably
1599-34-04	96-7/55.8	Atdabanian	16.3	0.2	40	12	38	12	31	13	15	14	probably
Dur_mt1599_@066			8.9	0.2									
Dur_mt1599_@067			9.1	0.2									
1599-34-05	96-7/55.8	Atdabanian	16.5	0.2	38	12	37	12	29	13	14	14	probably
1599-38-01	96-7/70	Botoman	13.6	0.2	52	11	50	12	43	12	27	13	yes
1599-38-02	96-7/70	Botoman	14.7	0.2	47	12	45	12	38	12	22	13	yes
1599-38-03	96-7/70	Botoman	10.5	0.2	65	11	64	11	56	11	41	12	yes
1599-38-04	96-7/70	Botoman	13.4	0.2	53	11	51	11	44	12	28	13	yes
1599-38-05	96-7/70	Botoman	9.9	0.2	68	11	67	11	59	11	44	12	yes
Dur_mt1599_@068			8.9	0.2									
Dur_mt1599_@069			9.0	0.2									
1599-38-06	96-7/70	Botoman	14.8	0.2	46	12	45	12	37	12	22	13	yes
1599-38-07	96-7/70	Botoman	16.0	0.2	41	12	39	12	32	13	16	14	probably
1599-38-08	96-7/70	Botoman	15.6	0.2	43	12	41	12	34	12	18	13	probably

1599-38-09	96-7/70	Botoman	15.4	0.2	43	12	42	12	34	12	19	13	yes
1599-39-01	96-7/70	Botoman	11.3	0.2	62	11	60	11	53	11	37	12	yes
1599-39-02	96-7/70	Botoman	10.1	0.2	67	11	66	11	58	11	43	12	yes
Dur_mt1599_@070			9.0	0.2									
Dur_mt1599_@071			8.8	0.2									
1599-39-03	96-7/70	Botoman	11.0	0.2	63	11	62	11	54	11	39	12	yes
1599-39-04	96-7/70	Botoman	12.6	0.2	56	11	54	11	47	12	31	13	yes
1599-39-05	96-7/70	Botoman	11.5	0.2	61	11	59	11	52	11	37	12	yes
1599-39-06	96-7/70	Botoman	12.0	0.2	59	11	57	11	50	12	34	12	yes
1599-39-07	96-7/70	Botoman	11.3	0.2	62	11	60	11	53	11	37	12	yes
1599-39-08	96-7/70	Botoman	15.9	0.2	41	12	39	12	32	13	16	14	probably
Dur_mt1599_@072			9.1	0.2									
Dur_mt1599_@073			8.9	0.2									
1599-39-09	96-7/70	Botoman	15.6	0.2	43	12	41	12	34	12	18	13	probably
1599-39-10	96-7/70	Botoman	16.2	0.2	40	12	38	12	31	13	15	14	probably
1599-39-11	96-7/70	Botoman	15.9	0.2	41	12	39	12	32	13	16	14	probably
1599-42-01	96B7/17	Tommotian	14.3	0.2	49	12	47	12	40	12	24	13	yes
1599-50-01	96-8/7.5	Toyonian	16.2	0.2	40	12	38	12	31	13	15	14	no
1599-50-02	96-8/7.5	Toyonian	16.3	0.2	40	12	38	12	31	13	15	14	no
Dur_mt1599_@074			9.1	0.2									
Dur_mt1599_@075			9.2	0.2									
1599-50-03	96-8/7.5	Toyonian	16.2	0.2	40	12	38	12	31	13	15	14	probably
1599-50-04	96-8/7.5	Toyonian	16.2	0.2	40	12	38	12	31	13	15	14	no
1599-50-05	96-8/7.5	Toyonian	15.8	0.2	42	12	40	12	33	12	17	14	probably
1599-50-06	96-8/7.5	Toyonian	16.6	0.2	38	12	36	12	29	13	13	14	no
1599-50-07	96-8/7.5	Toyonian	16.8	0.2	37	12	35	12	28	13	12	14	no
1599-50-08	96-8/7.5	Toyonian	16.6	0.2	38	12	37	12	29	13	14	14	no
Dur_mt1599_@076			9.2	0.2									
Dur_mt1599_@077			9.3	0.2									
1599-50-09	96-8/7.5	Toyonian	16.5	0.2	39	12	37	12	30	13	14	14	probably
1599-50-10	96-8/7.5	Toyonian	16.6	0.2	38	12	36	12	29	13	13	14	no
1599-50-11	96-8/7.5	Toyonian	16.4	0.2	39	12	37	12	30	13	14	14	no

1599-50-12	96-8/7.5	Toyonian	15.7	0.2	42	12	40	12	33	12	17	14	probably
1599-50-13	96-8/7.5	Toyonian	16.1	0.2	41	12	39	12	32	13	16	14	no
1599-50-14	96-8/7.5	Toyonian	16.6	0.2	38	12	36	12	29	13	14	14	no
Dur_mt1599_@078			9.0	0.2									
Dur_mt1599_@079			9.2	0.2									
1599-60-01	96-8/23.1	Amgan	16.4	0.2	39	12	37	12	30	13	14	14	no
1599-60-02	96-8/23.1	Amgan	16.3	0.2	40	12	38	12	31	13	15	14	no
1599-60-03	96-8/23.1	Amgan	16.0	0.2	41	12	39	12	32	13	16	14	probably
1599-60-04	96-8/23.1	Amgan	16.3	0.2	40	12	38	12	31	13	15	14	no
1599-60-05	96-8/23.1	Amgan	16.5	0.2	38	12	37	12	29	13	14	14	no
1599-60-06	96-8/23.1	Amgan	16.3	0.2	40	12	38	12	31	13	15	14	probably
Dur_mt1599_@080			9.1	0.2									
Dur_mt1599_@081			9.0	0.2									
Dur_mt1599_@082			9.1	0.2									
Dur_mt1599_@083			9.0	0.2									

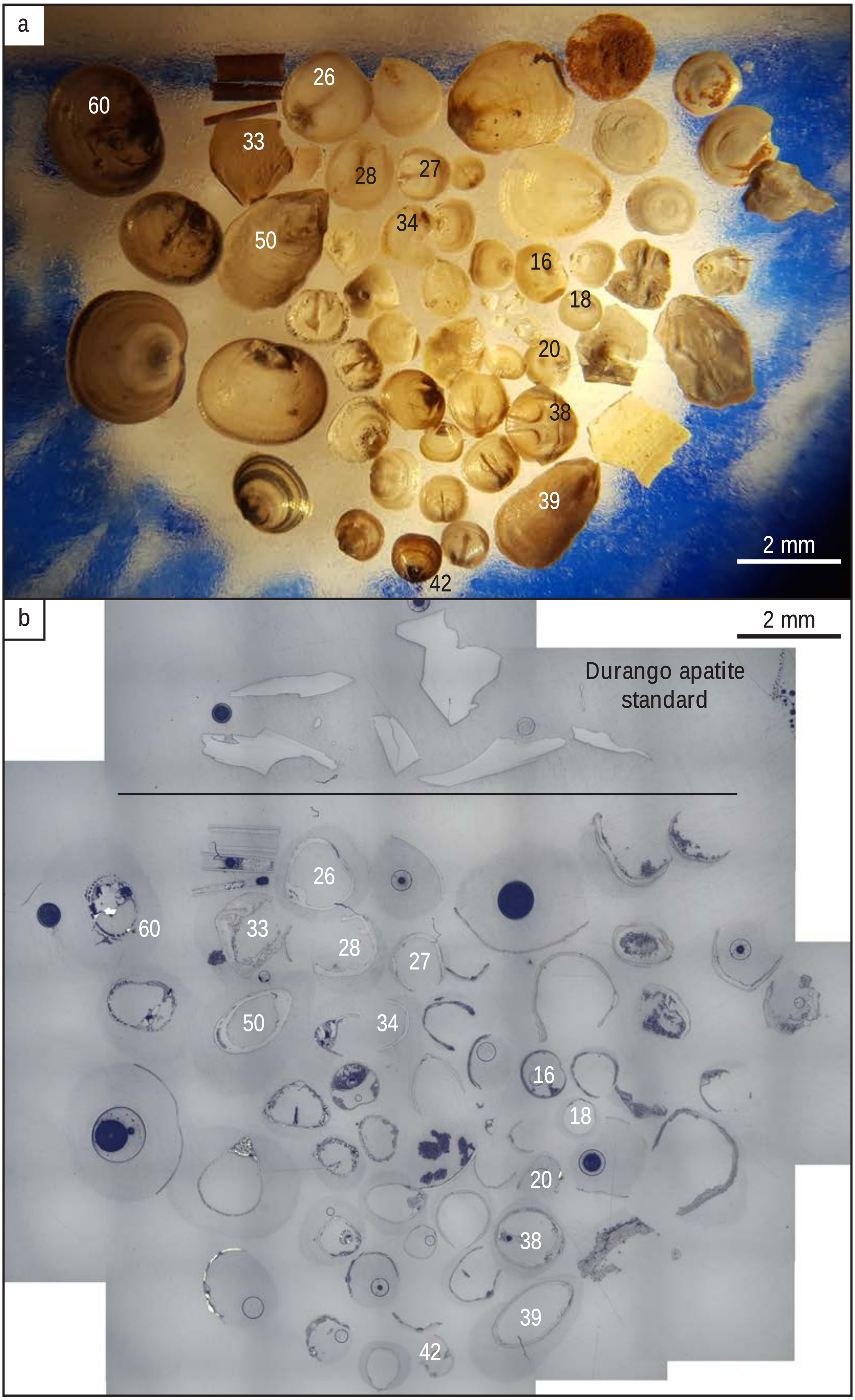

Supplementary Material 1. Images of mounted brachiopod shells and the polished stub 1599 including six grains of the Durango apatite standard. (a) Image of the brachiopod shells from stub 1599. Shells were mounted on a two-side tape fixed on acrylic glass. Numbers of samples reported herein are included. (b) Image of the polished stub 1599 generated with the Olympus cellSens Standard software.

Supplementary Material 2. Reflected light optical- and SEM images showing locations of spots analyzed. Reflected light optical images already indicate recrystallized individual shell portions, characterized by considerably darker and inhomogeneous appearance. This first order identification is confirmed by SEM images showing the brachiopod shell ultrastructure in detail. Shell material characterized by recrystallization, pores, laminae, or secondary fissures corresponds (even if not at all) with considerably lower $\delta^{18}O_{phosphate}$ values, thus interpreted as most probably representing diagenetic alteration. Dense crystalline shell material without indication for recrystallization and alteration is characterized by generally more positive $\delta^{18}O_{phosphate}$ values, thus appear to represent the most primary $\delta^{18}O_{seawater}$ signature.

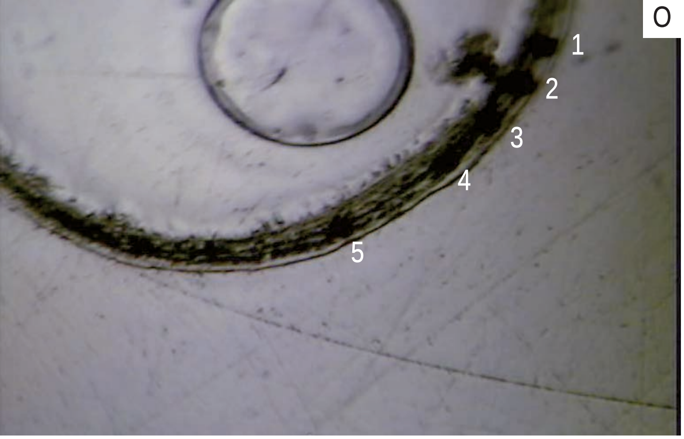

O Reflected light optical image showing locations of spots analyzed.

1–5 SEM microphotographs of the different analyzing spots. Various stages of recrystallization indicate a diagenetic alteration of the shell material and therewith most probably of the $\delta^{18}O$ isotopes. Progressive recrystallization corresponds with a successive lowering of the $\delta^{18}O$ signature, showing more positive values in less altered (13.8‰ at spot 1) and more negative values in strongly recrystallized shell material (10.5‰ and 10.6‰ at spots 4 and 5, respectively).

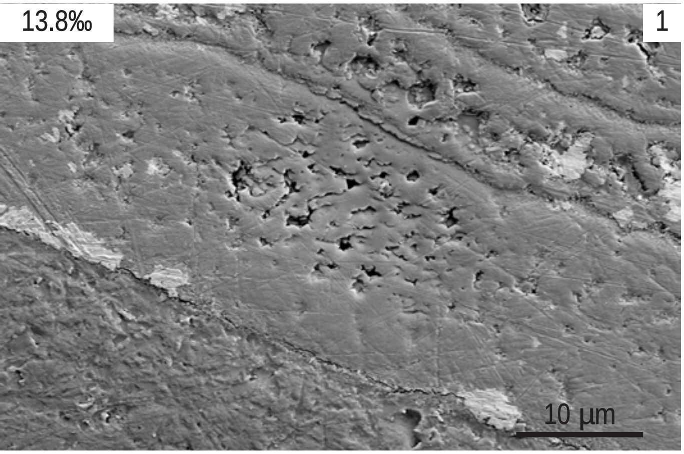

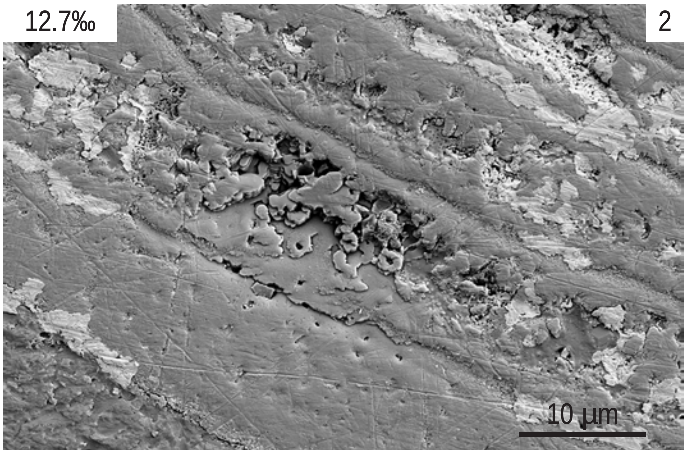

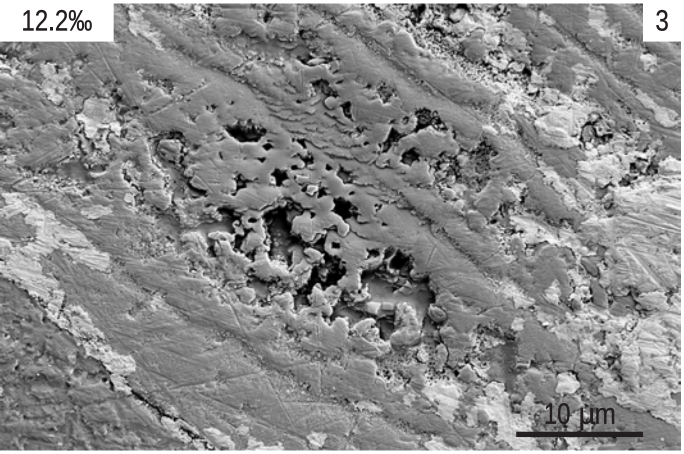

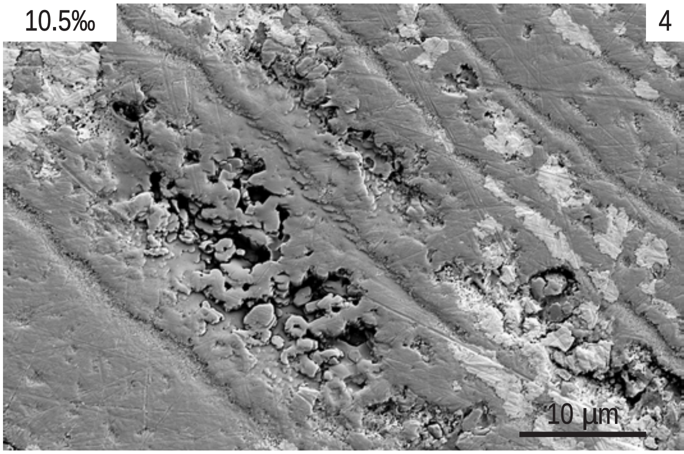

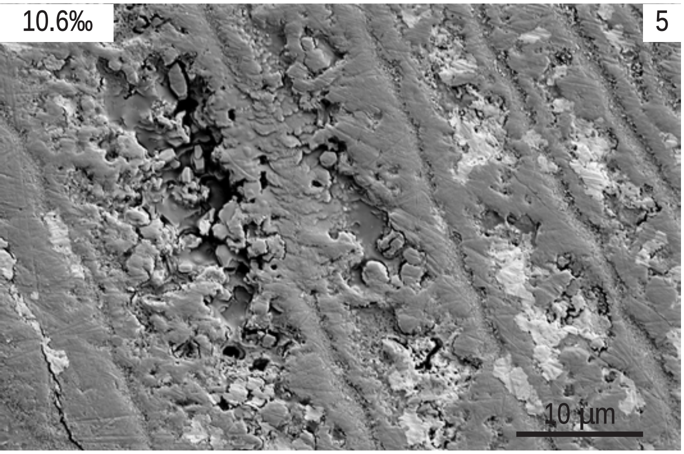

sample 1599-18

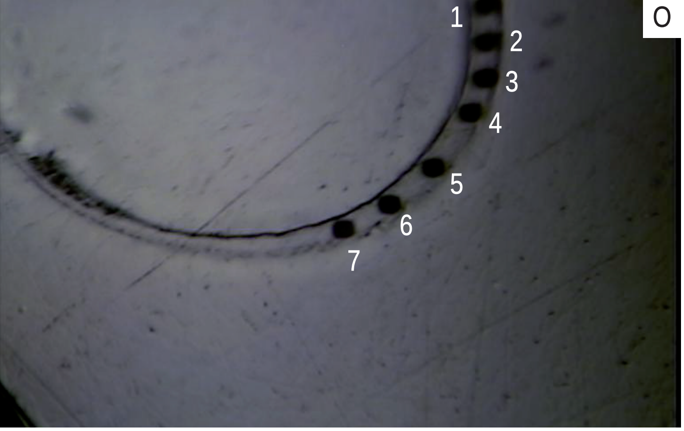

O Reflected light optical images showing locations of spots analyzed.

1–7 SEM microphotographs of the different analyzing spots. The porous shell material seems to be an ideal pathway for fluid migration and therewith for diagenetic alteration. This process is reflected by more negative $\delta^{18}O$ values corresponding with enhanced temperatures. Fissures potentiate the alteration process and its influence on the $\delta^{18}O$ signal (spot 5; 14.5‰).

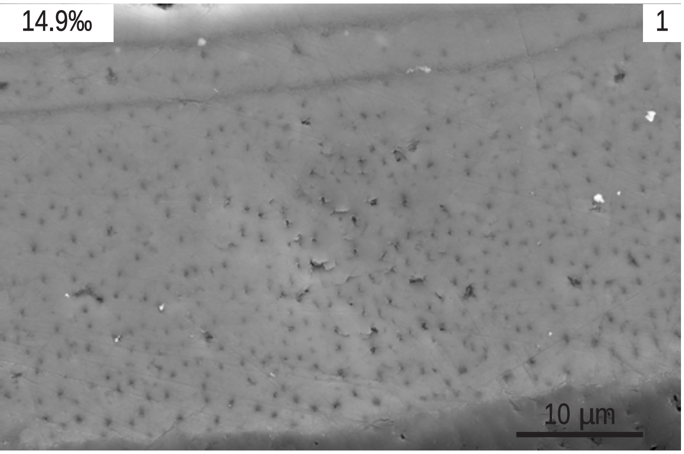

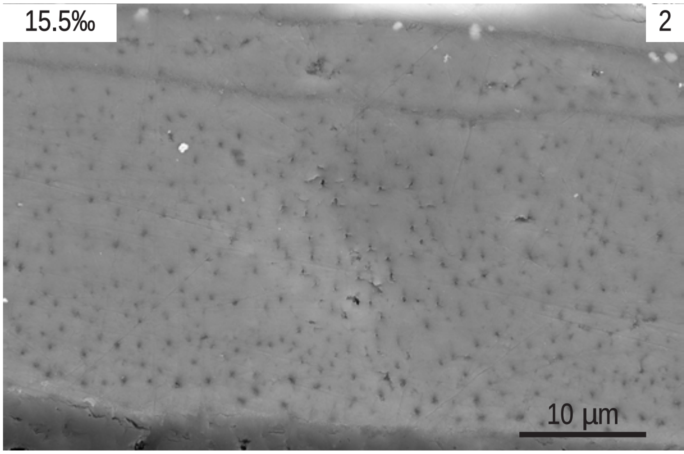

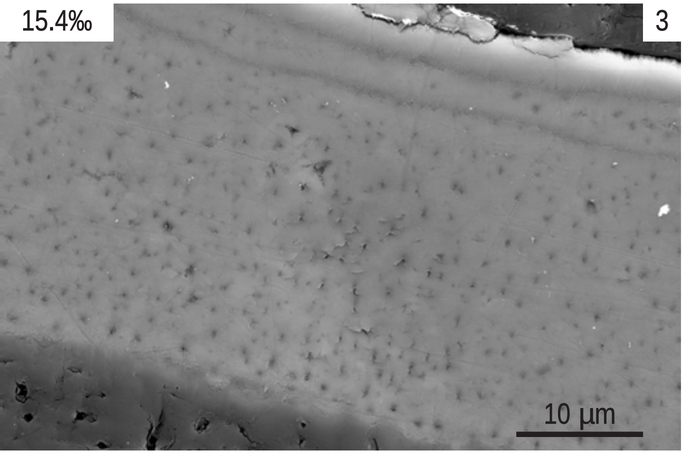

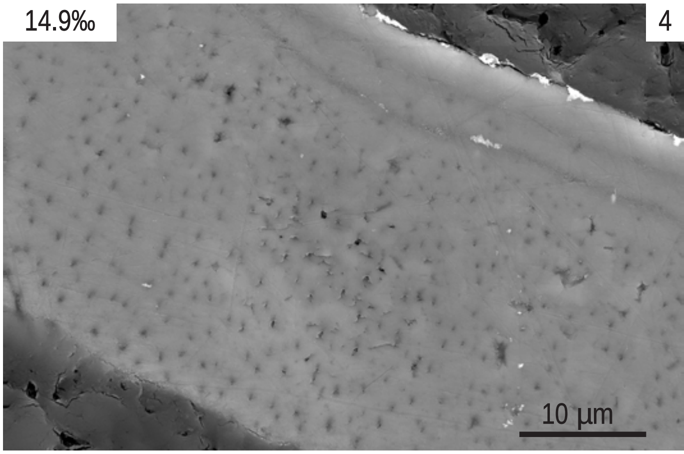

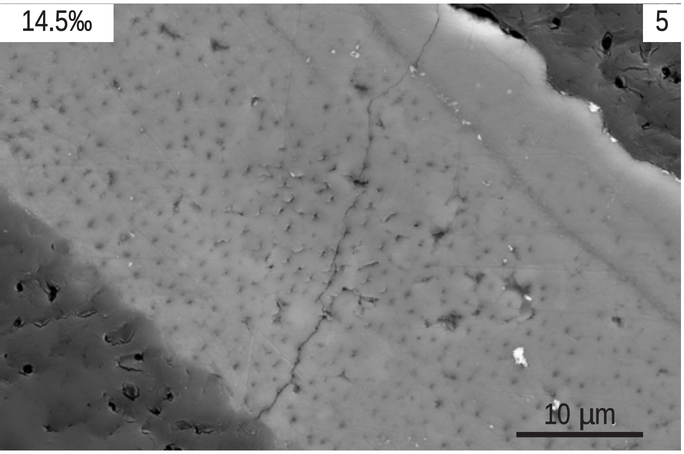

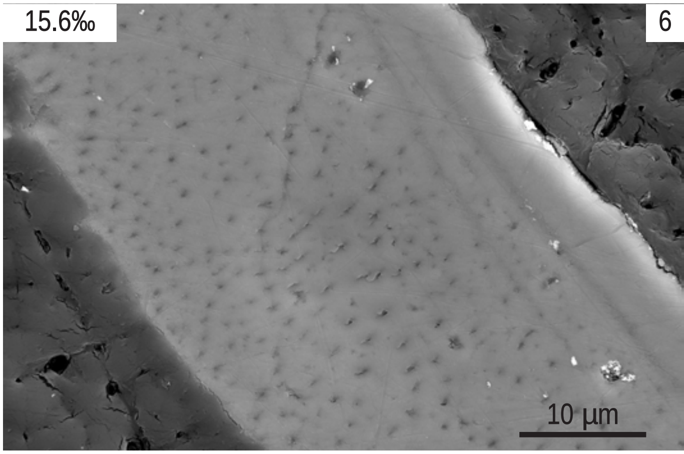

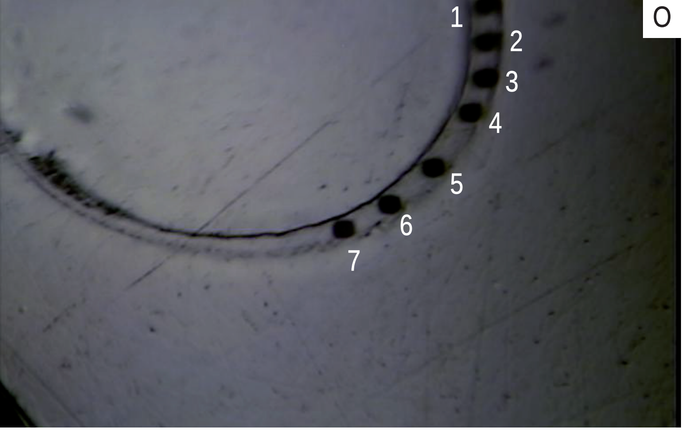

O Reflected light optical images showing locations of spots analyzed.

1–7 SEM microphotographs of the different analyzing spots. The porous shell material seems to be an ideal pathway for fluid migration and therewith for diagenetic alteration. This process is reflected by more negative $\delta^{18}O$ values corresponding with enhanced temperatures. Fissures potentiate the alteration process and its influence on the $\delta^{18}O$ signal (spot 5; 14.5‰).

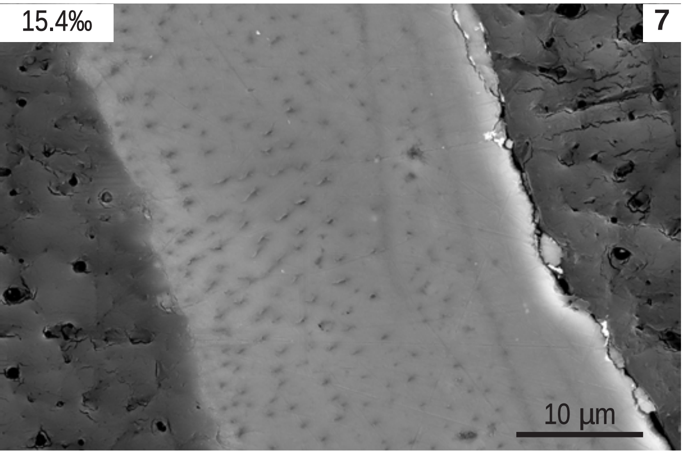

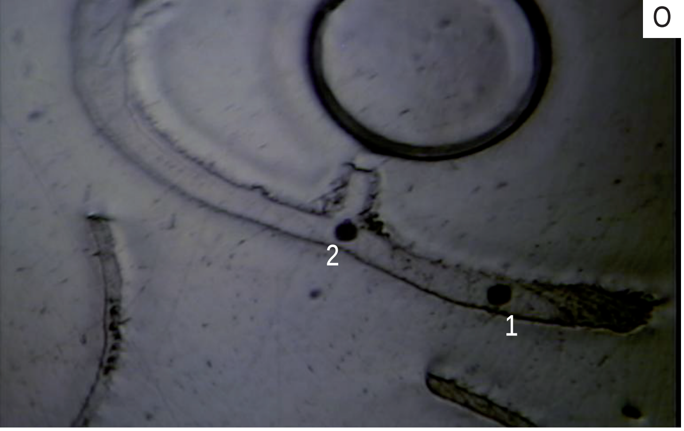

O Reflected light optical images showing locations of spots analyzed.

1–7 SEM microphotographs of the different analyzing spots covering laminated and recrystallized to porous shell material. Spotted parts showing laminae and/or huge pores corresponding with more negative $\delta^{18}O$ values (15.3‰, 16.0‰, and 15.9‰ at spots 1, 6, and 7). Progressive recrystallization, recognizable by the transition from dense crystalline towards coarse crystalline shell parts (spots 2–4) corresponds with a continuous decrease in the $\delta^{18}O$ signature.

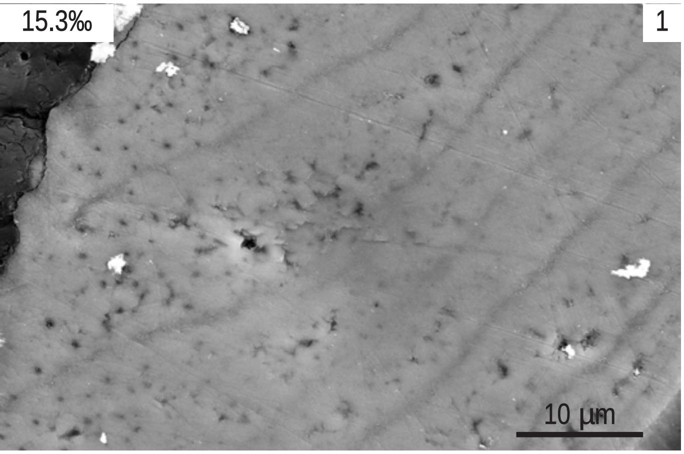

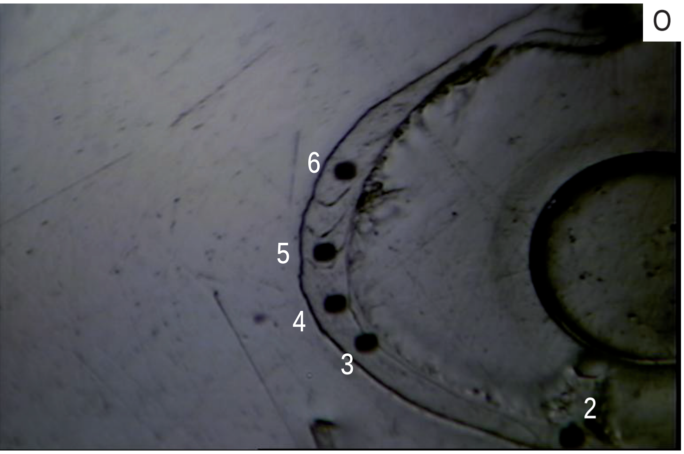

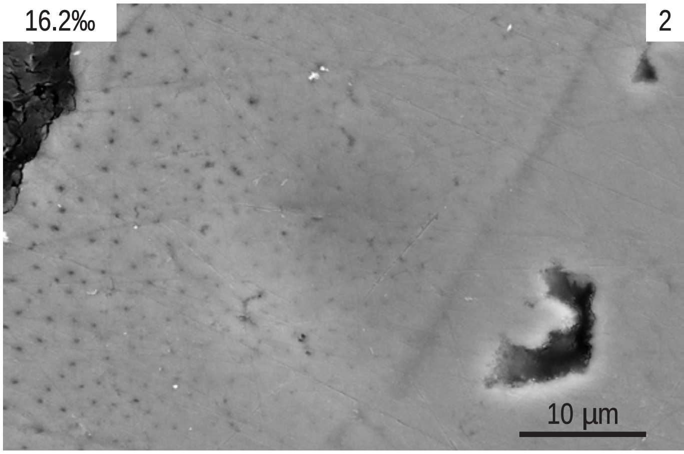

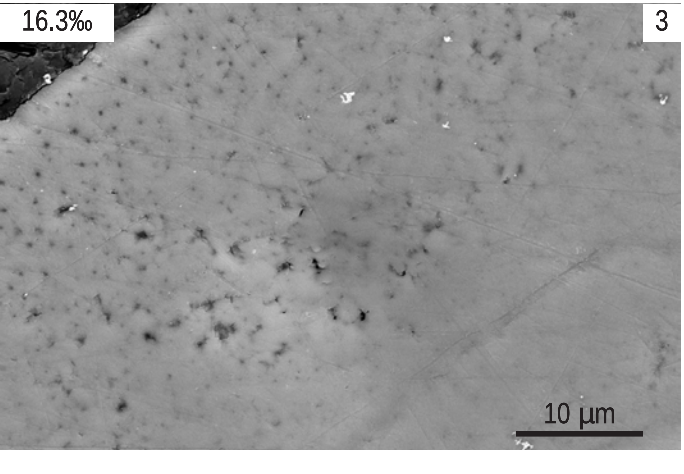

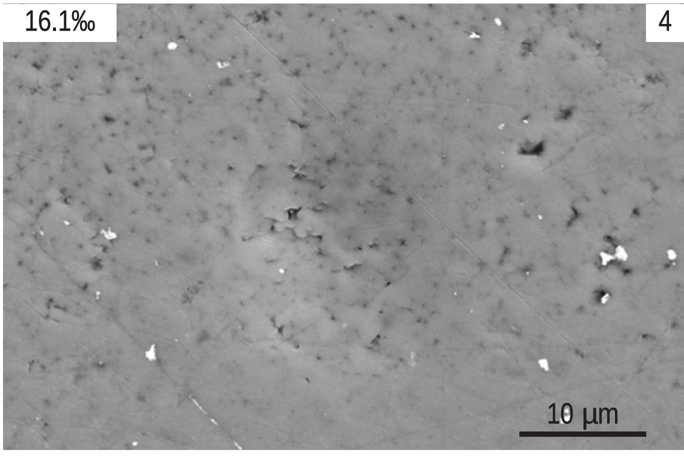

sample 1599-20

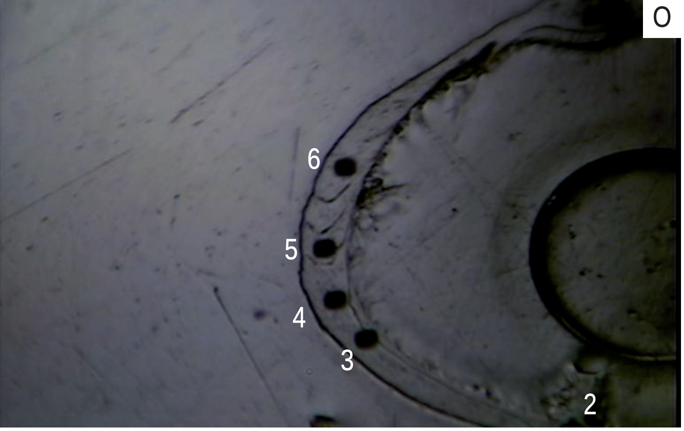

O Reflected light optical images showing locations of spots analyzed.

1–7 SEM microphotographs of the different analyzing spots covering laminated and recrystallized to porous shell material. Spotted parts showing laminae and/or huge pores corresponding with more negative $\delta^{18}O$ values (15.3‰, 16.0‰, and 15.9‰ at spots 1, 6, and 7). Progressive recrystallization, recognizable by the transition from dense crystalline towards coarse crystalline shell parts (spots 2–4) corresponds with a continuous decrease in the $\delta^{18}O$ signature.

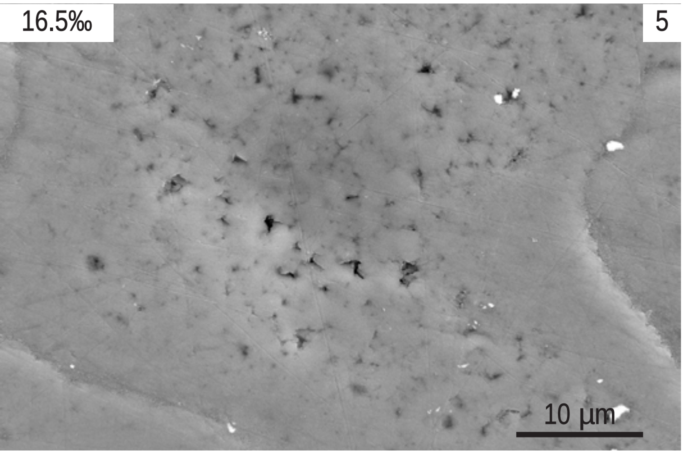

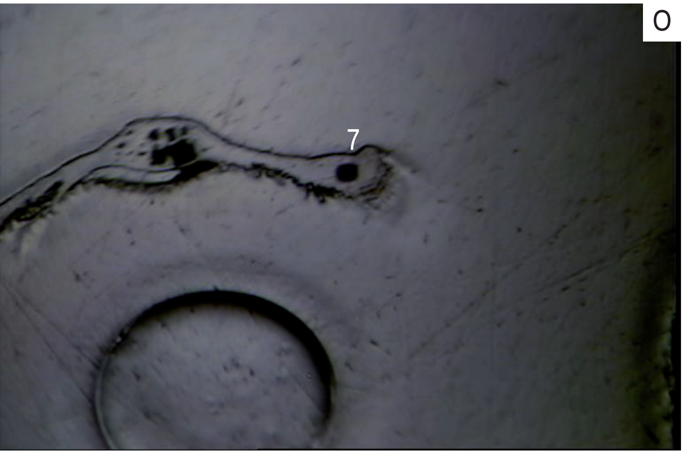

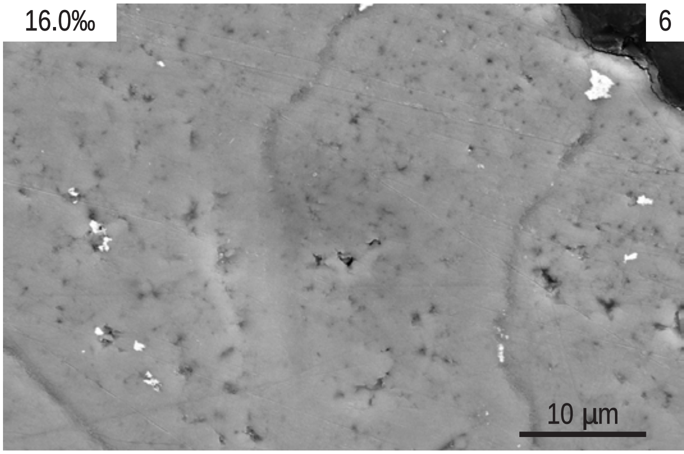

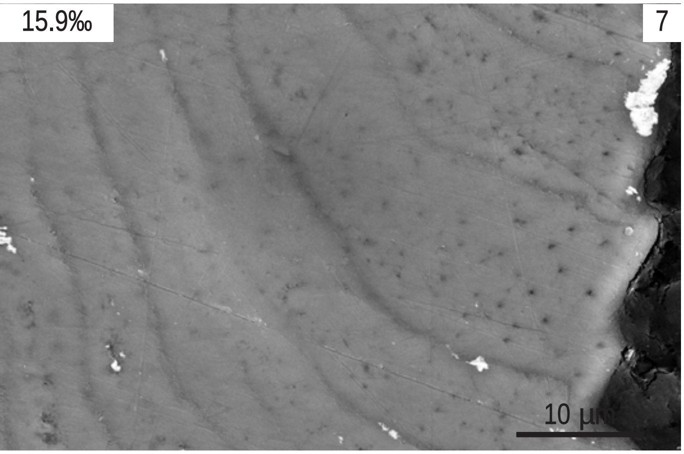

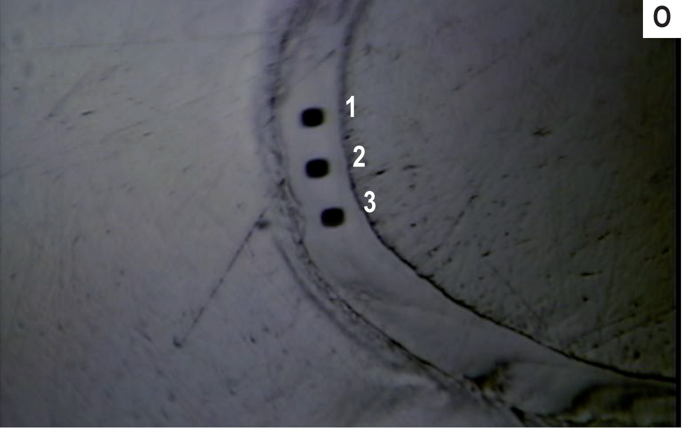

O Reflected light optical images showing locations of spots analyzed.

1–9 SEM microphotographs identify dense (spots 4–9) to fine crystalline shell material (spots 1–3). Even if spots 1–4 show the most positive $\delta^{18}O$ values (except spot 3), alteration cannot be excluded due to initial recrystallization and fissures. Shell material of spots 5–9 is considered to represent the most primary structure.

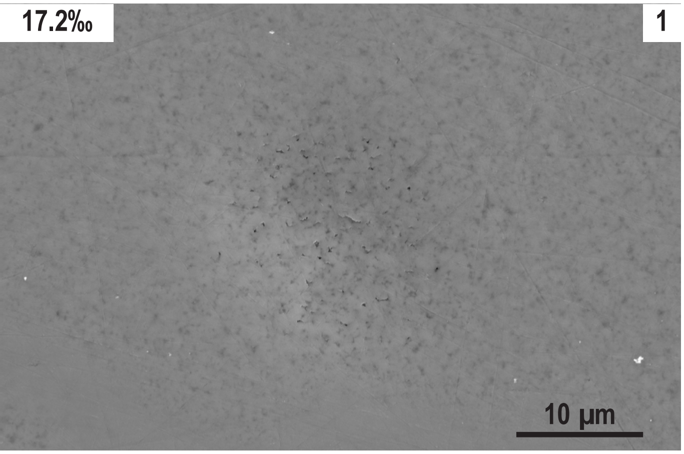

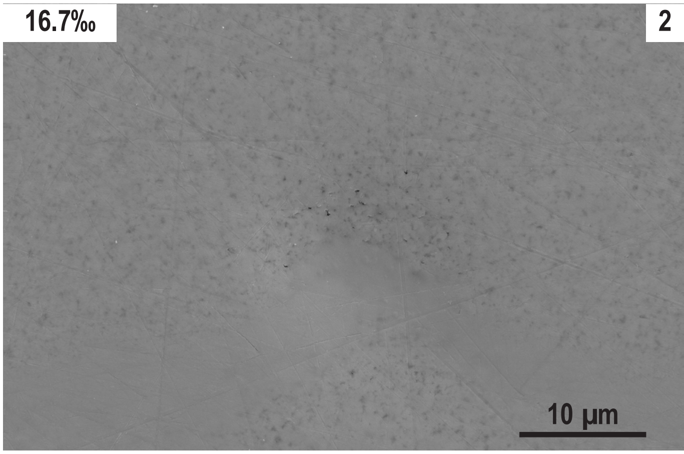

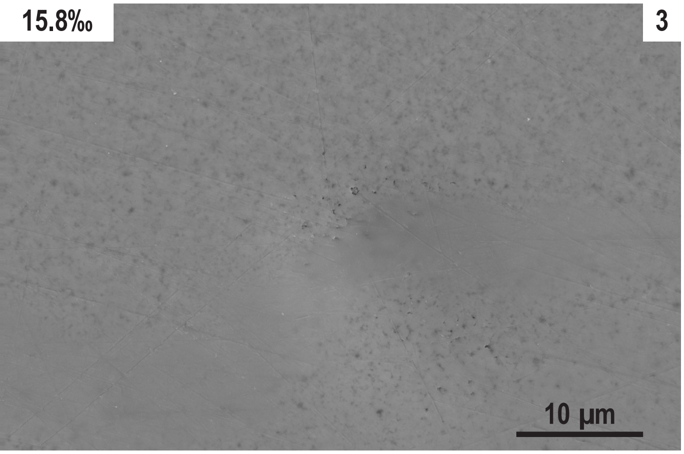

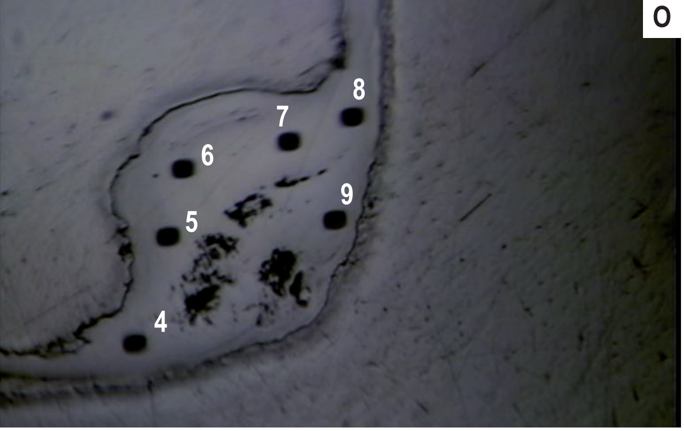

O Reflected light optical images showing locations of spots analyzed.

1–9 SEM microphotographs identify dense (spots 4–9) to fine crystalline shell material (spots 1–3). Even if spots 1–4 show the most positive δ^{18}O values (except spot 3), alteration cannot be excluded due to initial recrystallization and fissures. Shell material of spots 5–9 is considered to represent the most primary structure.

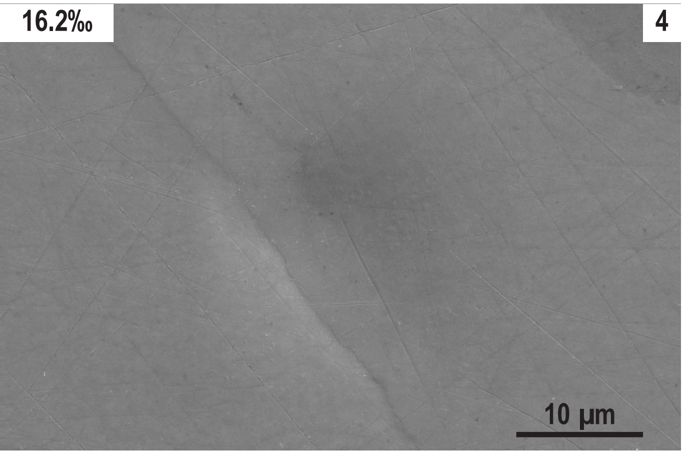

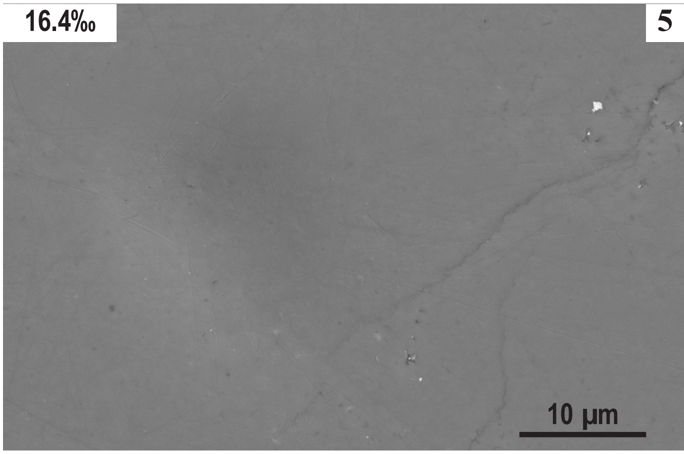

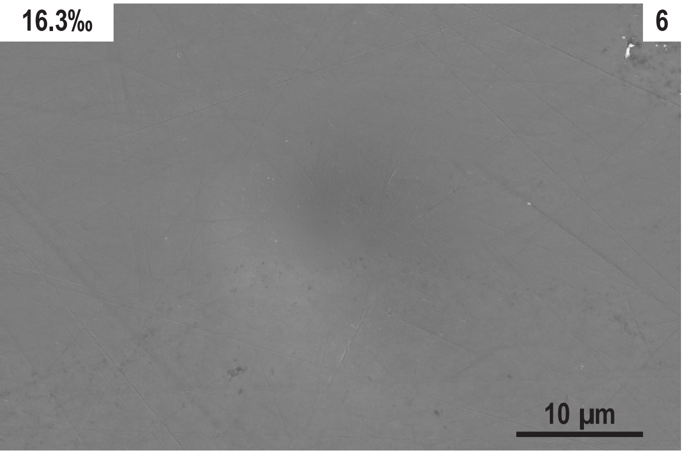

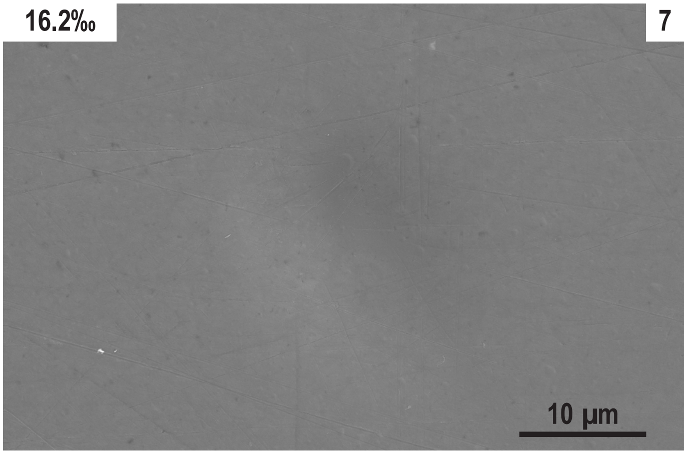

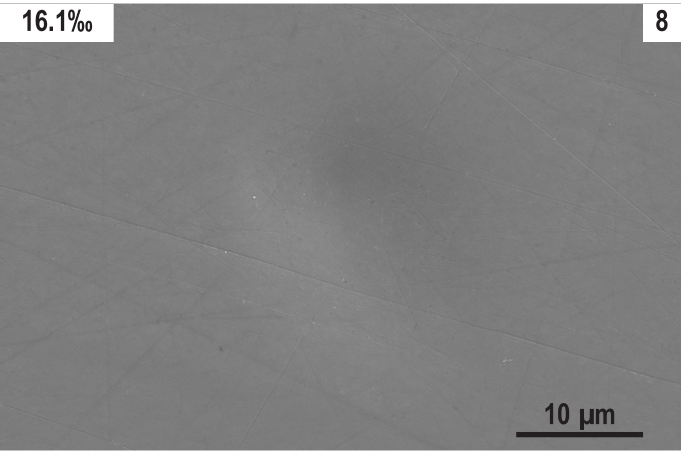

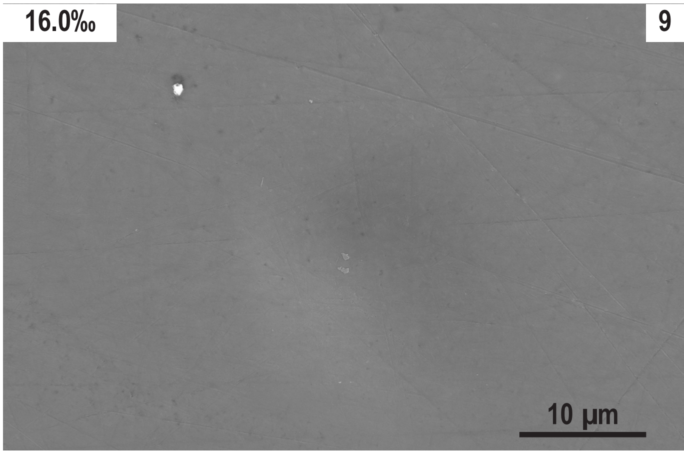

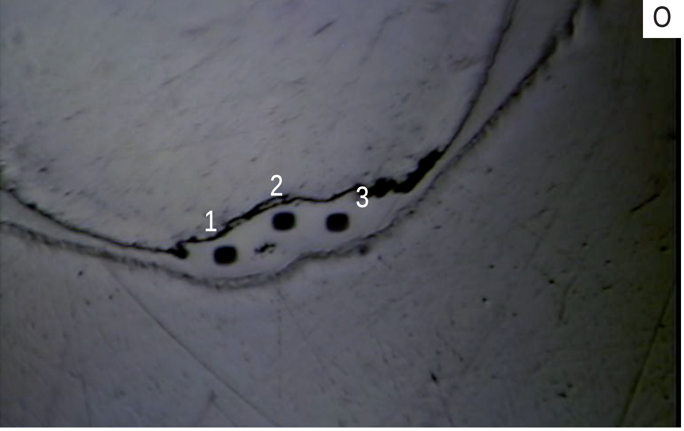

O Reflected light optical images showing locations of spots analyzed.

1–7 SEM microphotographs identify dense to fine crystalline shell material, most probably excluding significant diagenetic alteration. Coarser crystalline, partly porous shell portions (spots 4–7) probably indicate partial recrystallization and correspond with slightly more positive $\delta^{18}O$ values.

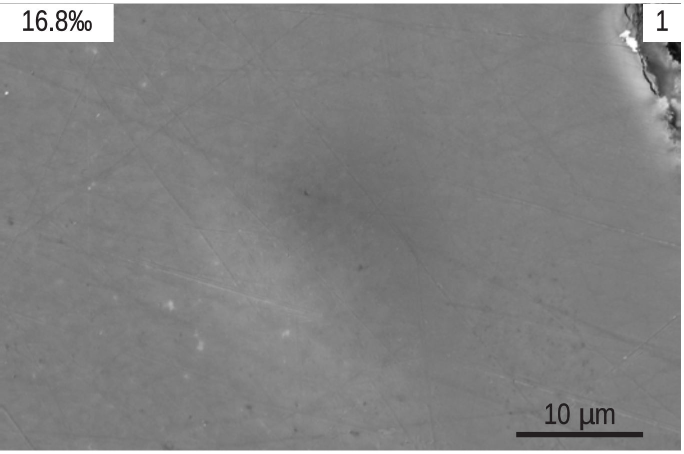

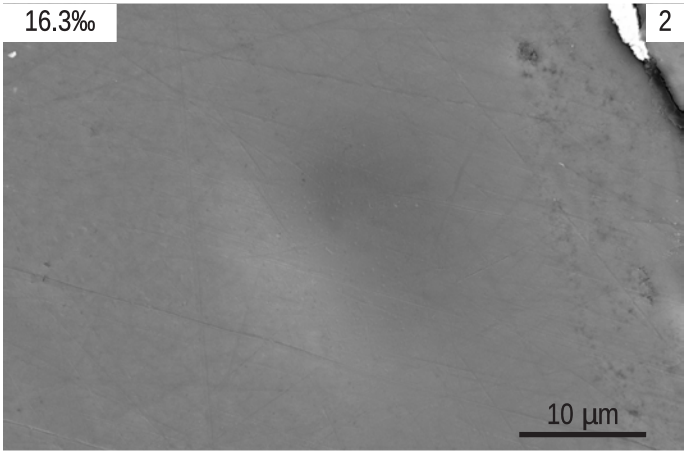

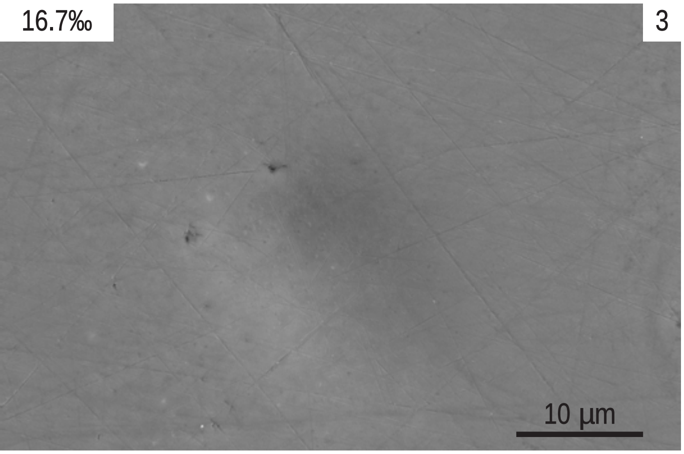

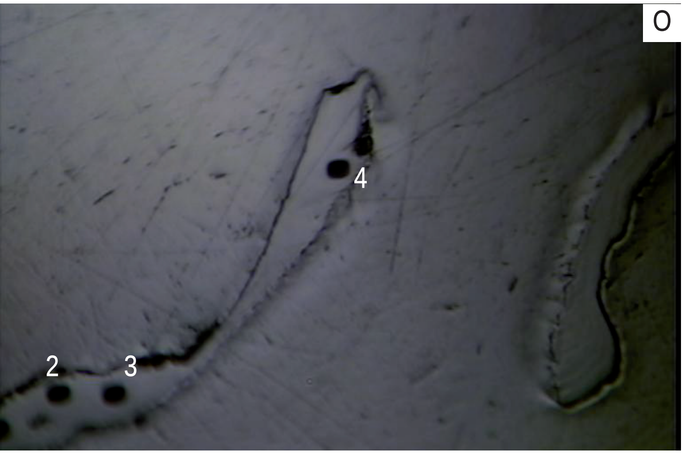

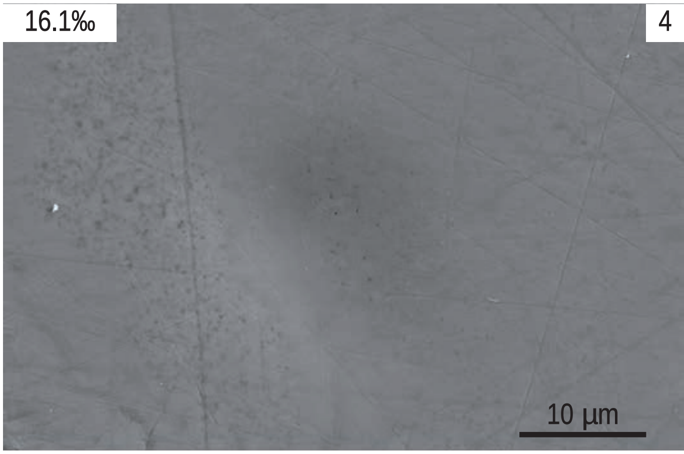

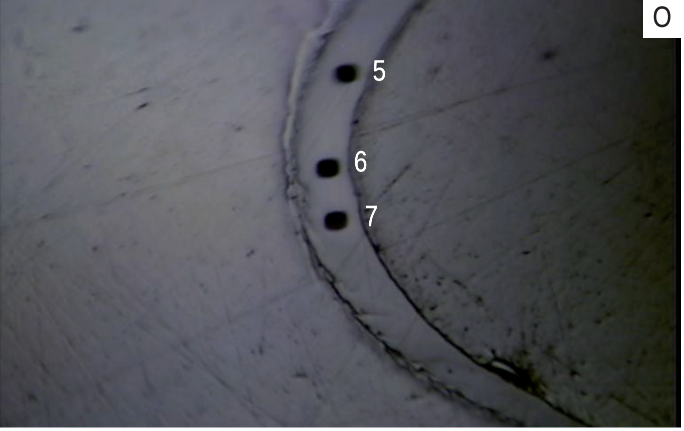

O Reflected light optical images showing locations of spots analyzed.

1–7 SEM microphotographs identify dense to fine crystalline shell material, most probably excluding significant diagenetic alteration. Coarser crystalline, partly porous shell portions (spots 4–7) probably indicate partial recrystallization and correspond with slightly more positive $\delta^{18}O$ values.

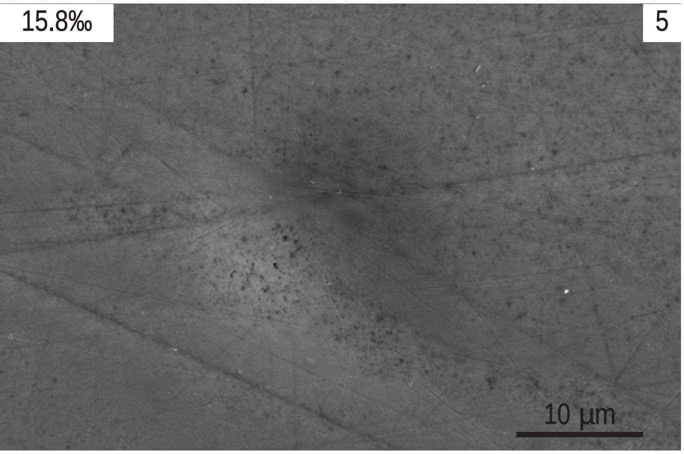

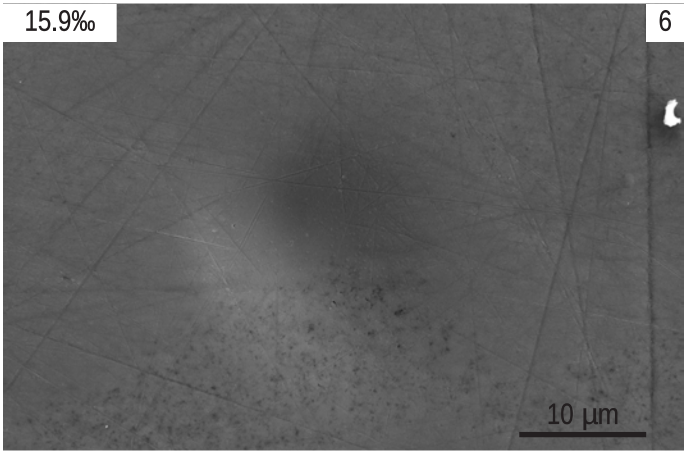

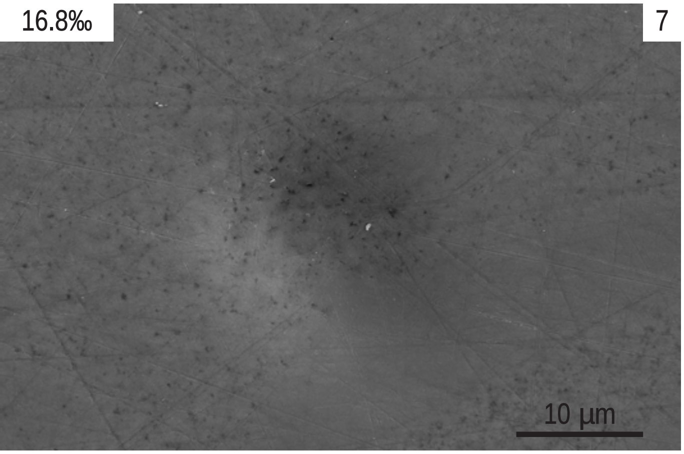

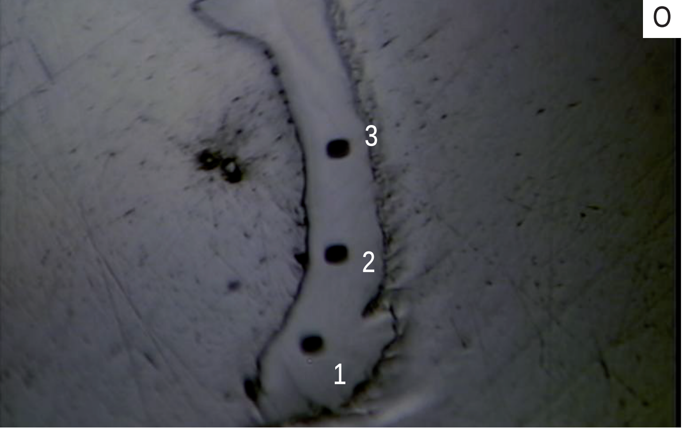

O Reflected light optical images showing locations of spots analyzed.

1–12 SEM images show dense shell material without any indication of recrystallization (spots 1, 2, 4, 7, 8, 9, 11, 12). It is characterized by the lowest $\delta^{18}O$ values. A slightly more negative $\delta^{18}O$ value at spot 3 (16.0‰) is probably affected by nearby fracture. Extremely low $\delta^{18}O$ values (spots 5 & 6) correspond with recrystallized shell portions.

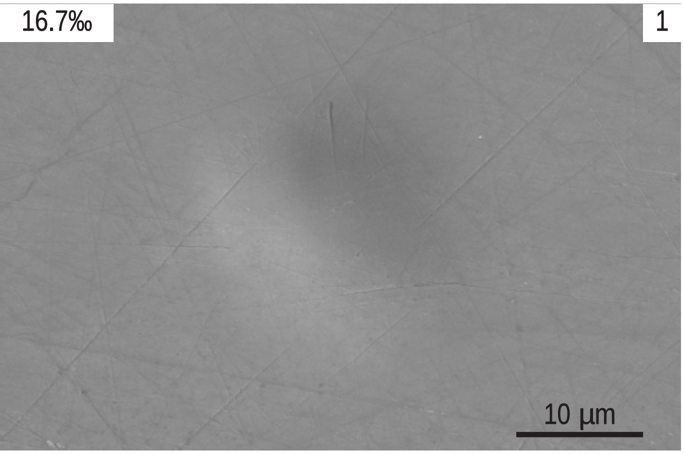

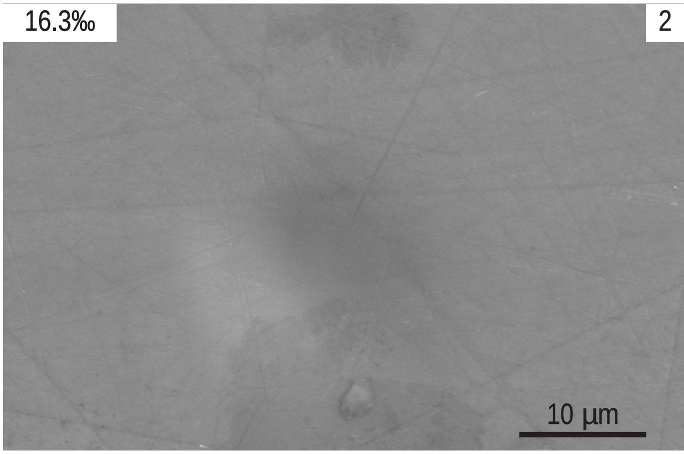

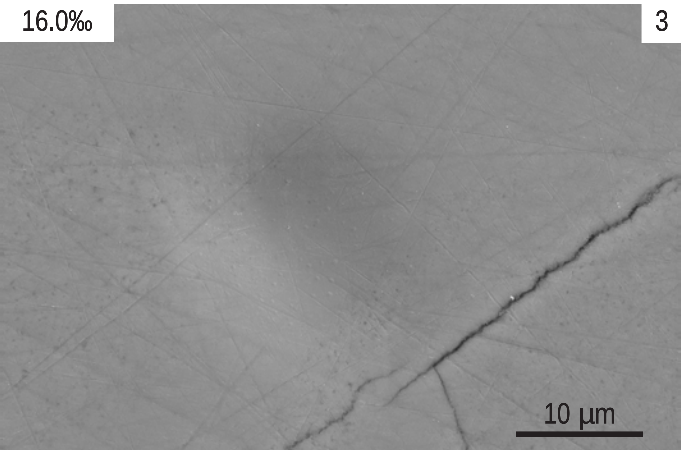

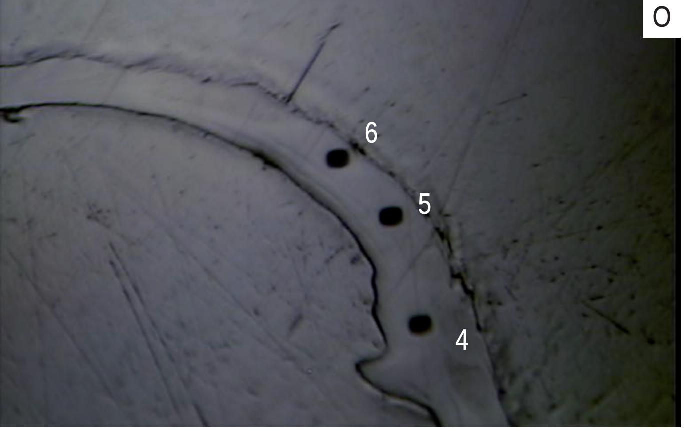

O Reflected light optical images showing locations of spots analyzed.

1–12 SEM images show dense shell material without any indication of recrystallization (spots 1, 2, 4, 7, 8, 9, 11, 12). It is characterized by the lowest $\delta^{18}O$ values. A slightly more negative $\delta^{18}O$ value at spot 3 (16.0‰) is probably affected by nearby fracture. Extremely low $\delta^{18}O$ values (spots 5 & 6) correspond with recrystallized shell portions.

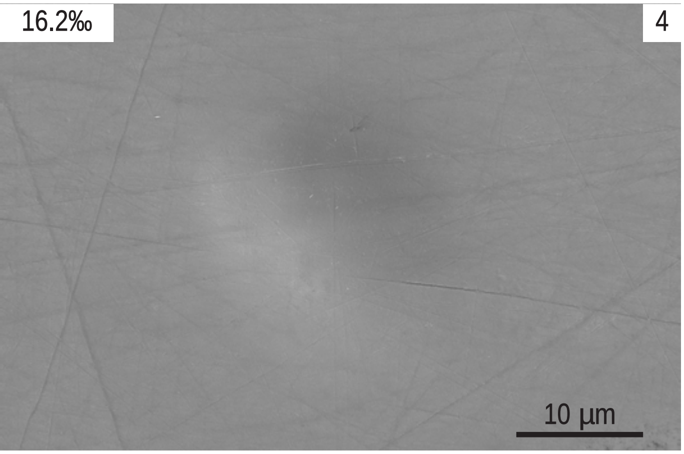

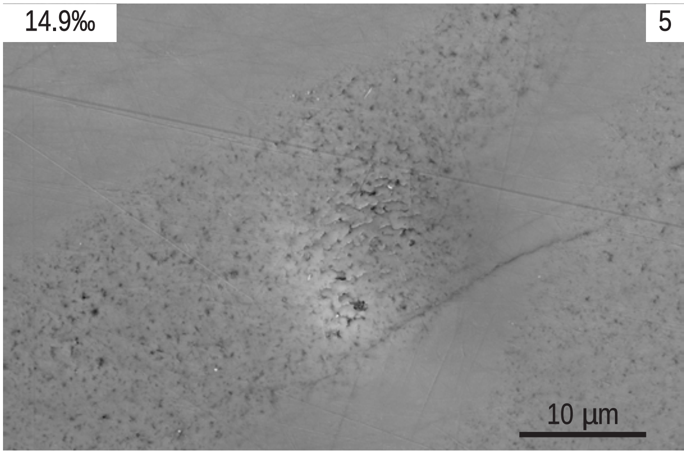

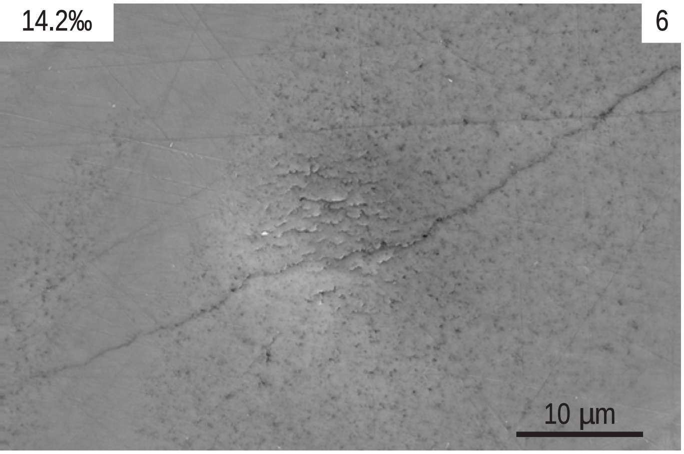

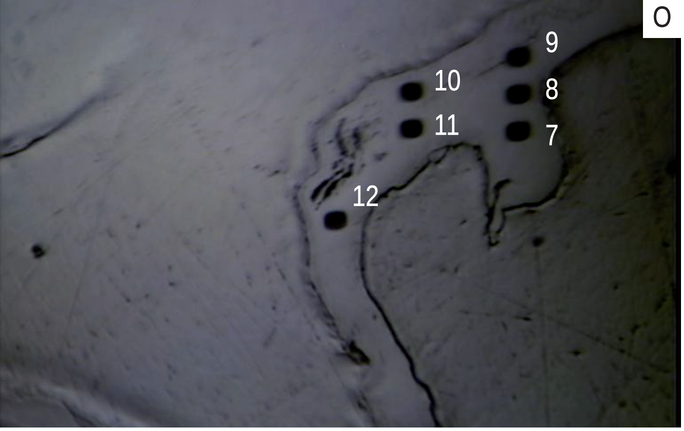

O Reflected light optical images showing locations of spots analyzed.

1–12 SEM images show dense shell material without any indication of recrystallization (spots 1, 2, 4, 7, 8, 9, 11, 12). It is characterized by the lowest $\delta^{18}O$ values. A slightly more negative $\delta^{18}O$ value at spot 3 (16.0‰) is probably affected by nearby fracture. Extremely low $\delta^{18}O$ values (spots 5 & 6) correspond with recrystallized shell portions.

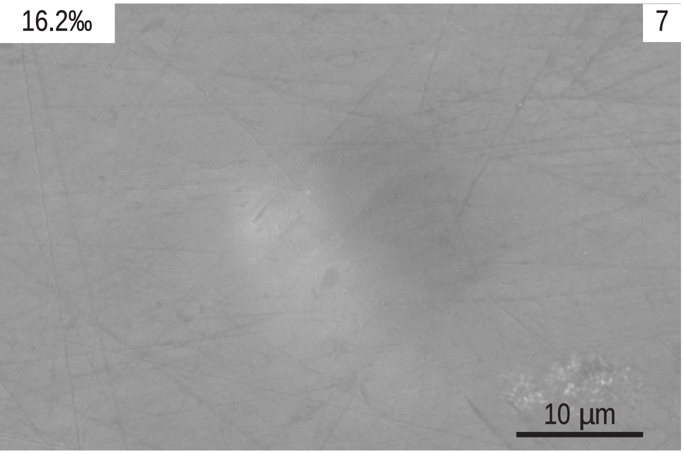

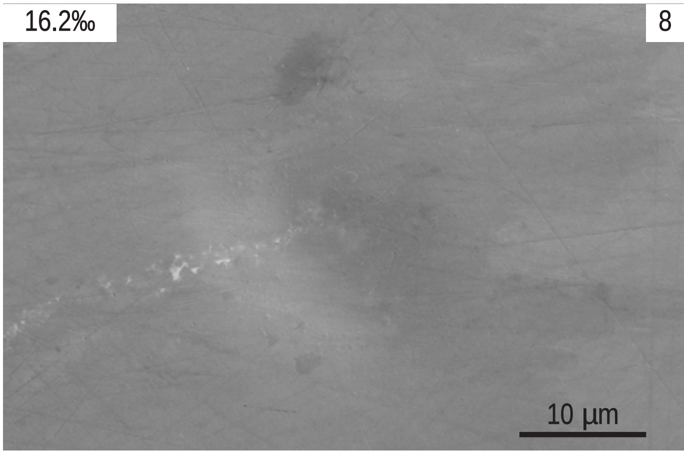

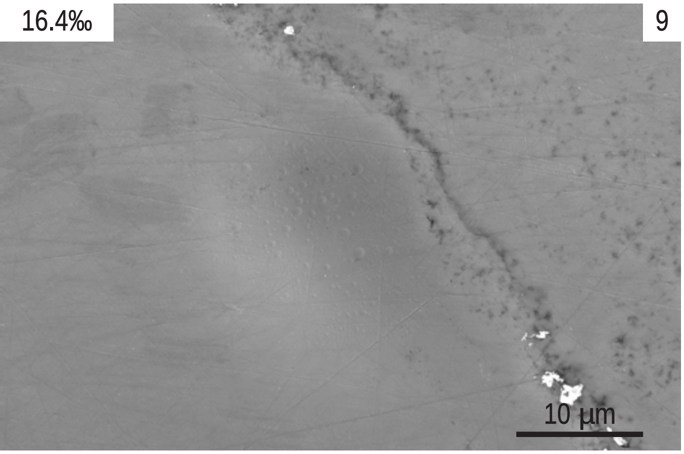

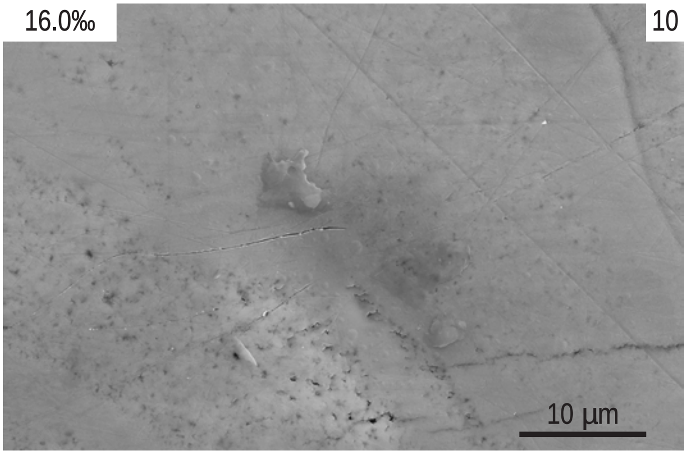

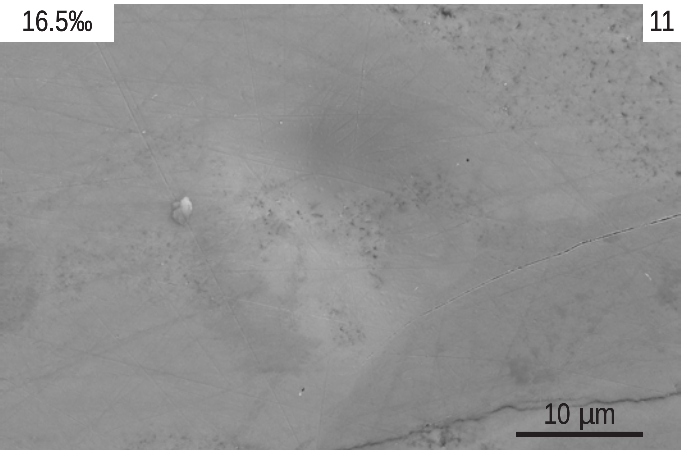

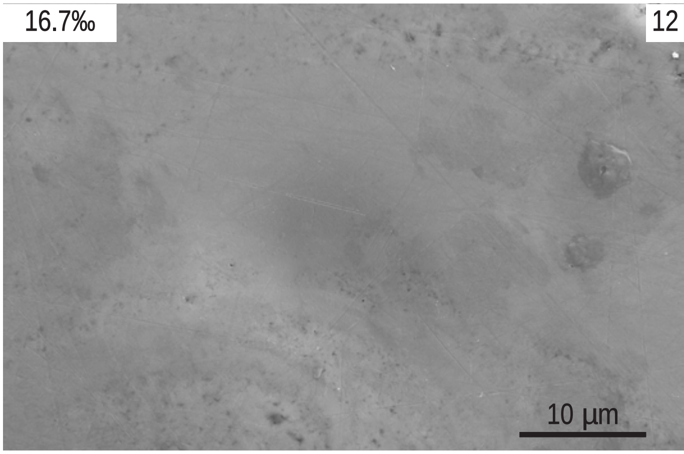

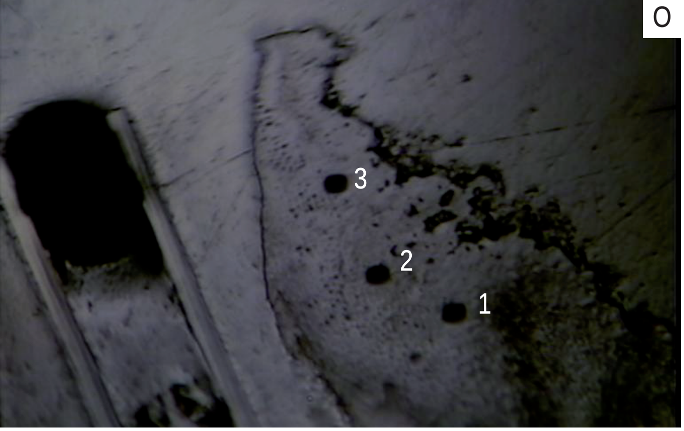

O Reflected light optical image showing locations of spots analyzed.

1–3 SEM microphotographs show recrystallized shell material. Fissures occur (spots 2 & 3). Based on these observations, diagenetic alteration of the $\delta^{18}O$ values seems to be obvious.

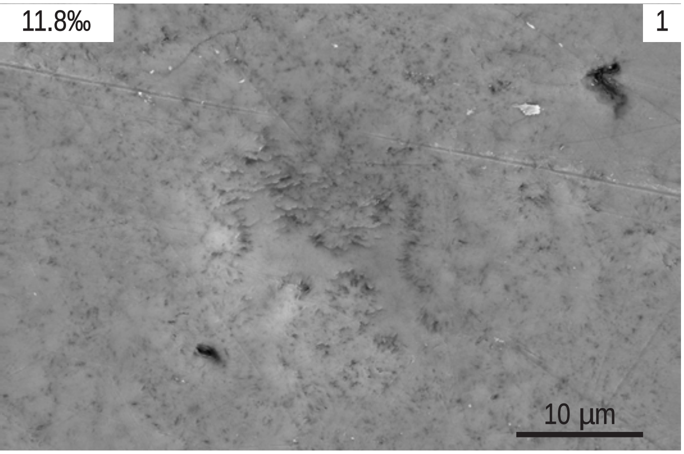

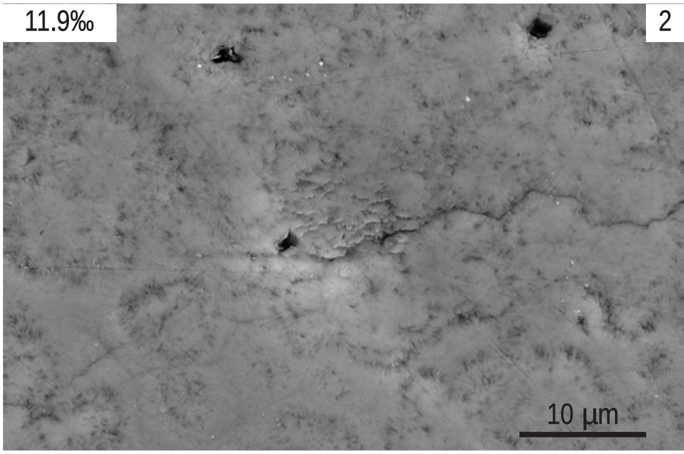

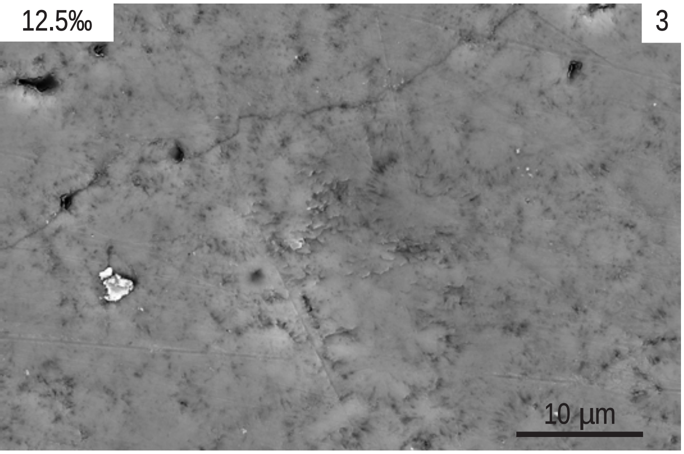

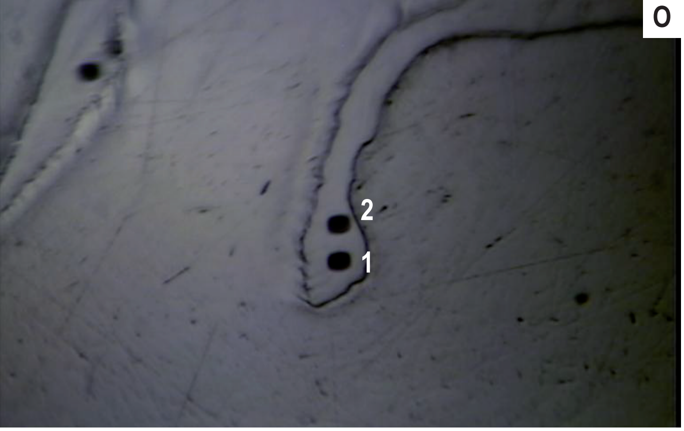

O Reflected light optical images showing locations of spots analyzed.

1–5 SEM microphotographs show dense to fine crystalline shell material. $\delta^{18}O$ values show no obvious indication for alteration. Coarser crystalline shell portions indicate partial recrystallization. Thus, alteration of the $\delta^{18}O$ signatures cannot be excluded.

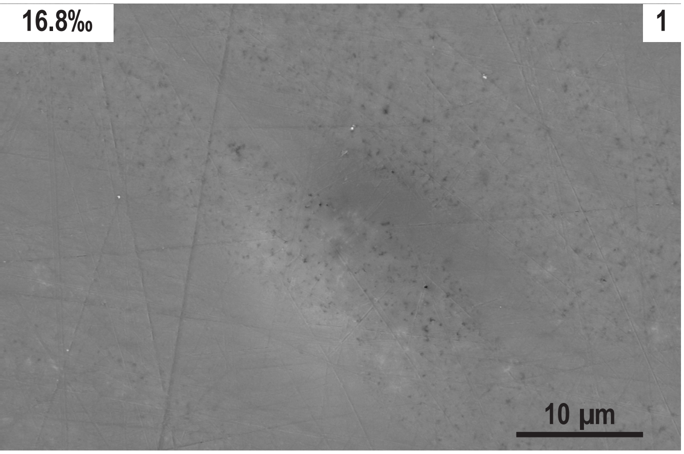

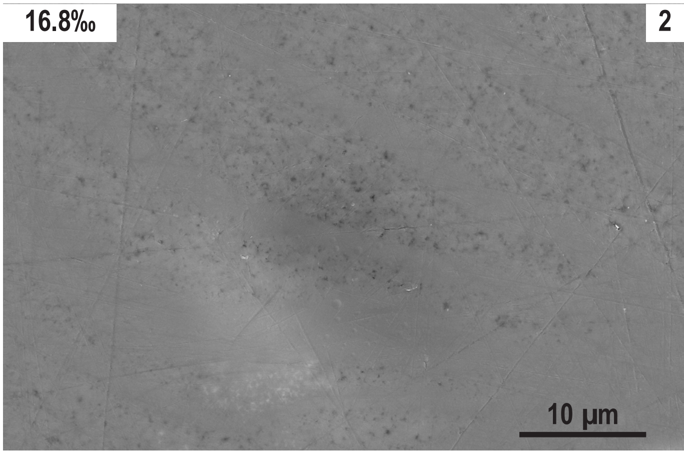

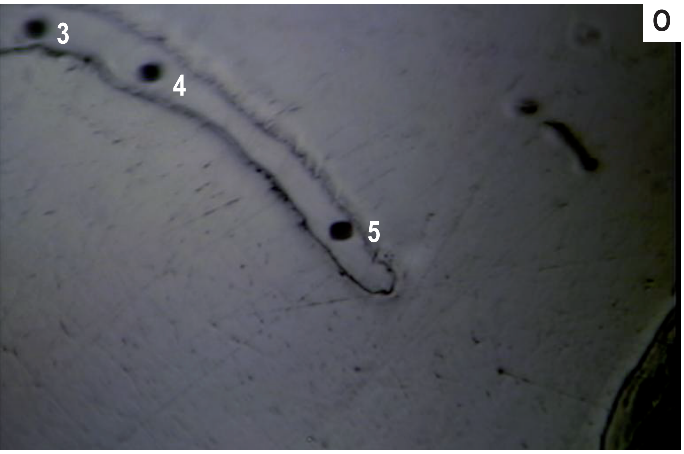

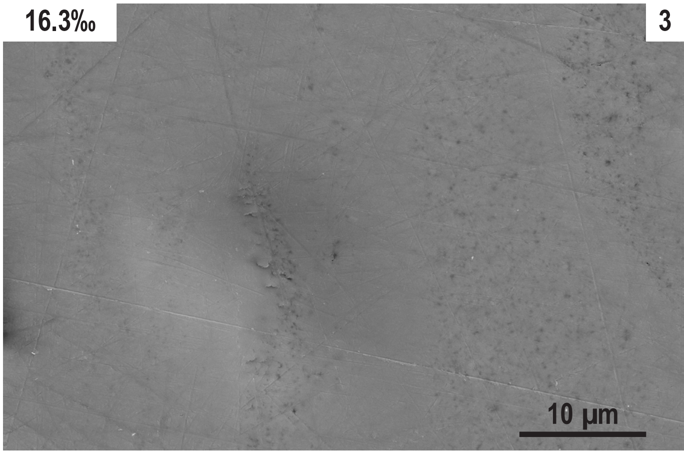

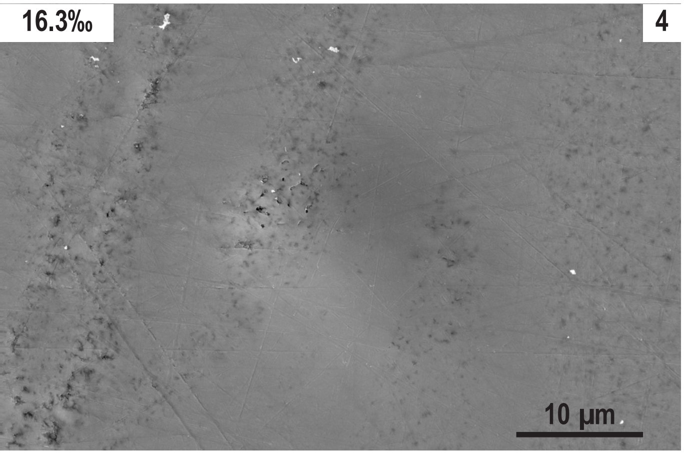

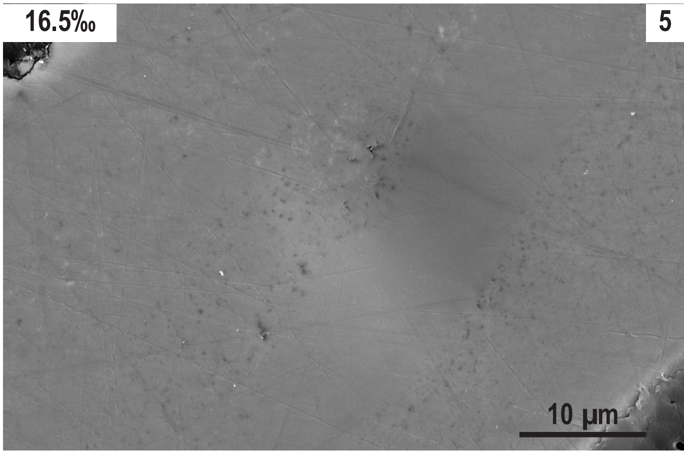

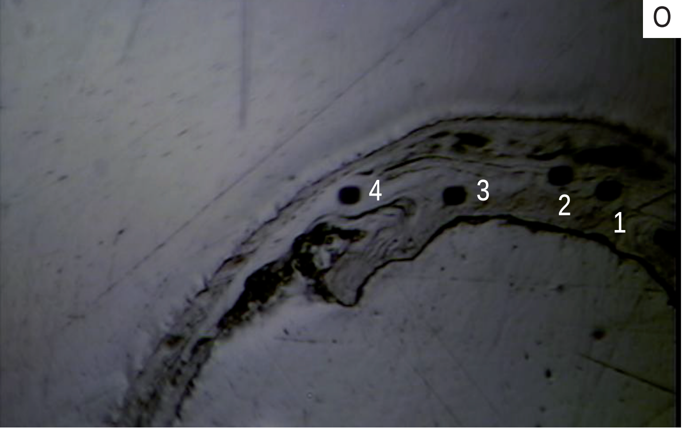

O Reflected light optical images showing locations of spots analyzed.

1–9 SEM microphotographs of laminated, recrystallized (and often porous) shell material, partly with fissures. Recrystallization and fissures make diagenetic alteration of the primary $\delta^{18}O$ signatures most probable. There are also indications for initial recrystallization processes at spots 7 and 8, thus would explain more negative $\delta^{18}O$ values.

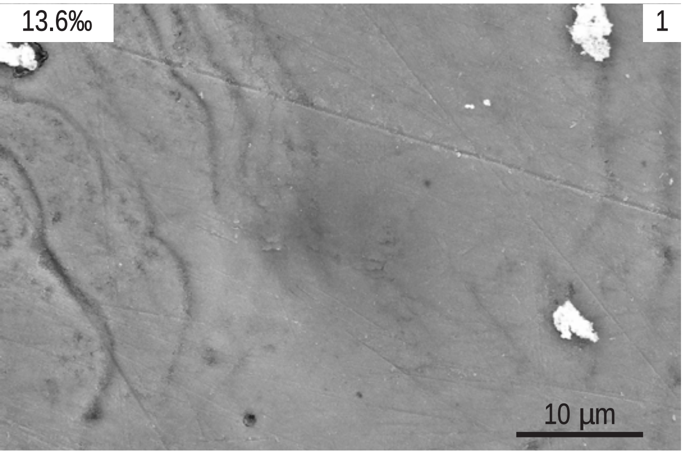

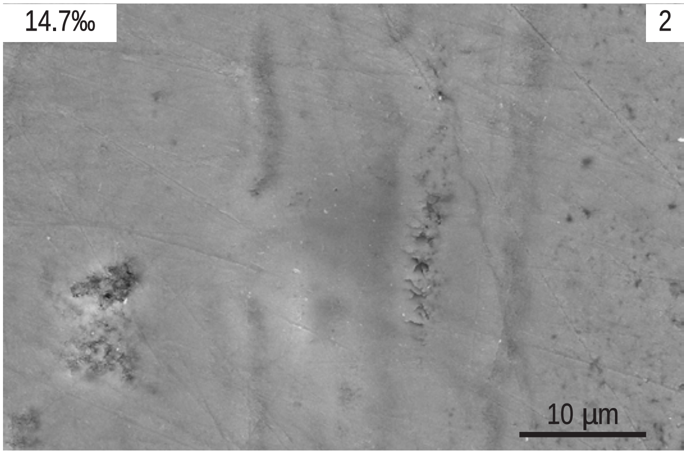

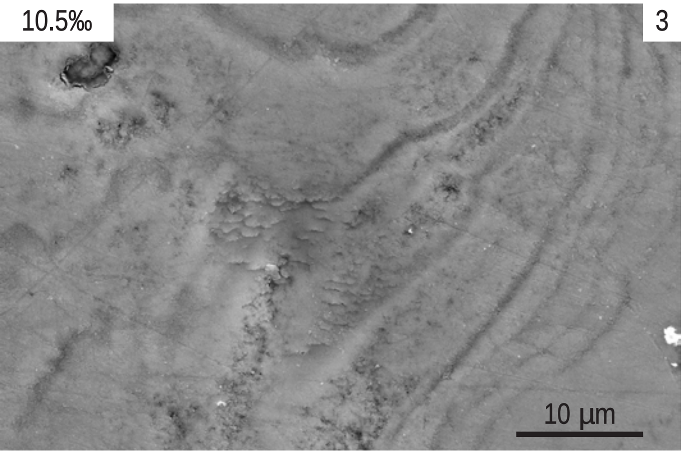

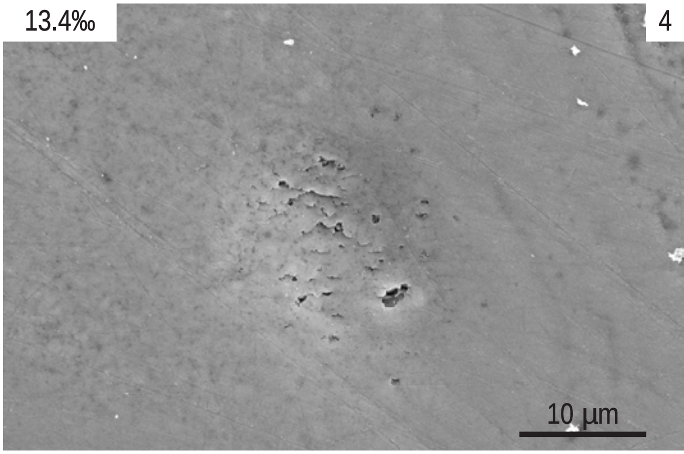

sample 1599-38

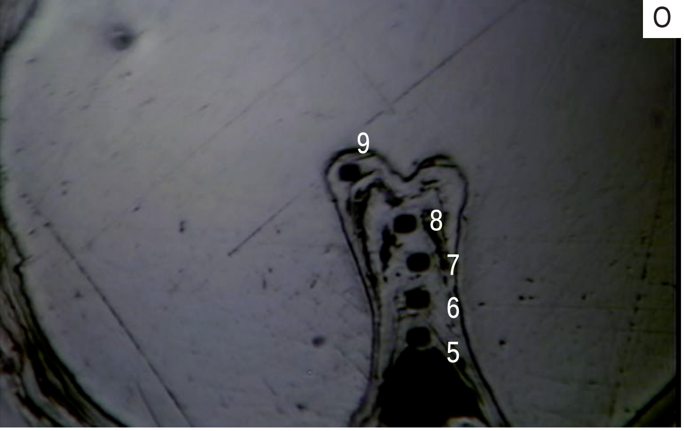

O Reflected light optical images showing locations of spots analyzed.

1–9 SEM microphotographs of laminated, recrystallized (and often porous) shell material, partly with fissures. Recrystallization and fissures make diagenetic alteration of the primary $\delta^{18}O$ signatures most probable. There are also indications for initial recrystallization processes at spots 7 and 8, thus would explain more negative $\delta^{18}O$ values.

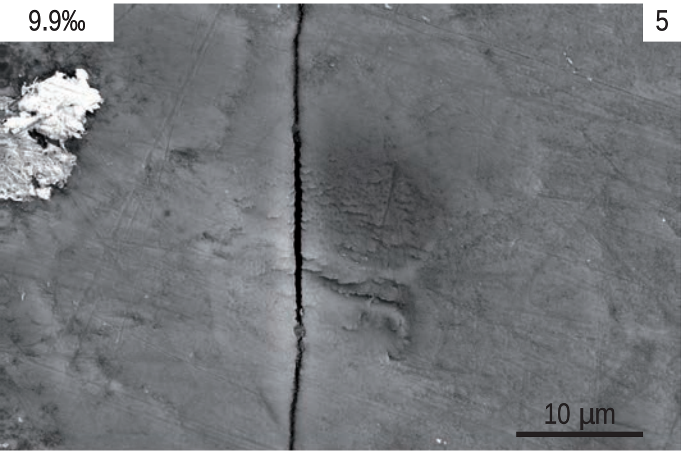

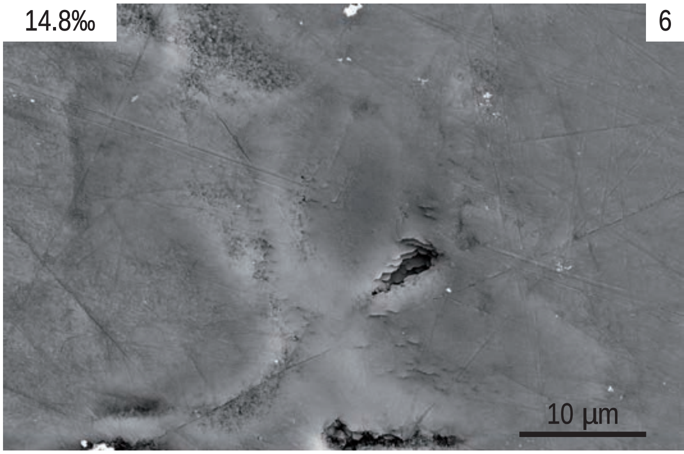

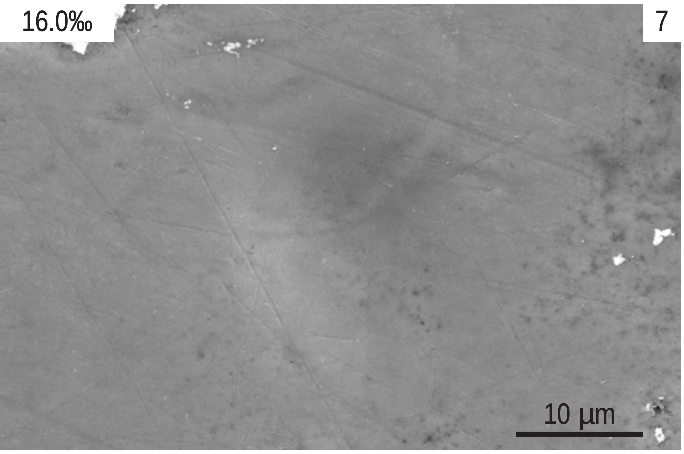

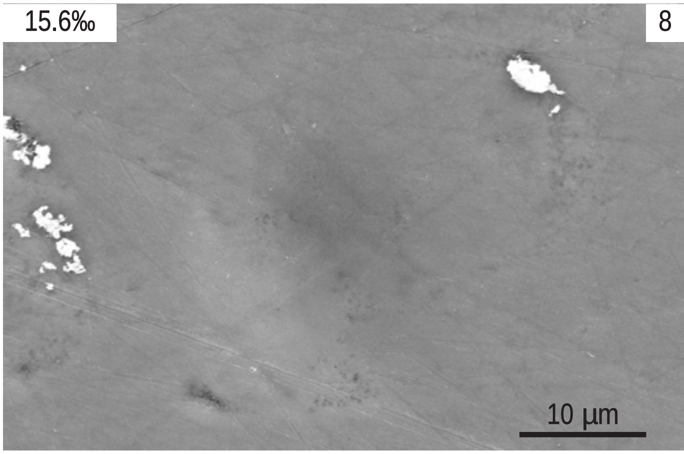

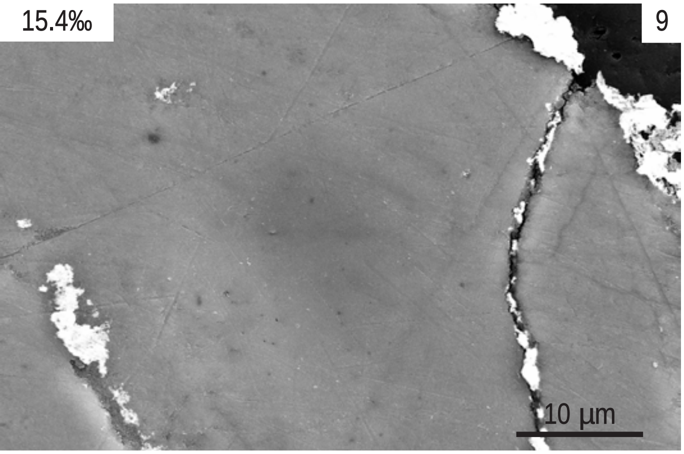

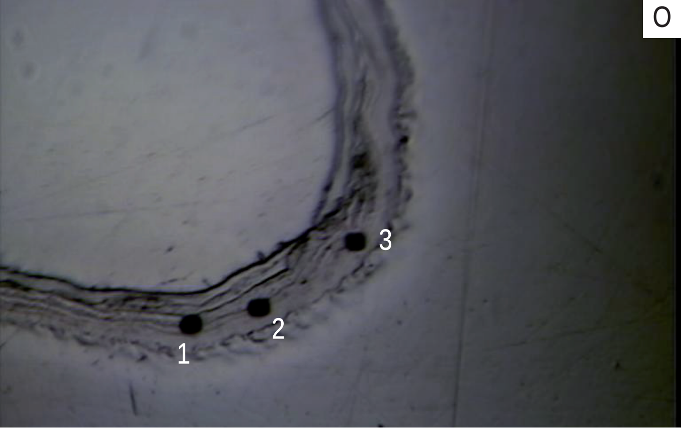

O Reflected light optical images showing locations of spots analyzed.

1–11 SEM microphotographs show recrystallized shell material, patly with fissures. Alteration of the $\delta^{18}O$ values obtained from such portions (spots 1–7) seems to be obvious. Spots 8–11 are located within dense–fine crystalline shell material. Initial recrystallization could be observed at these spots, most probably explains the slightly more negative $\delta^{18}O$ values.

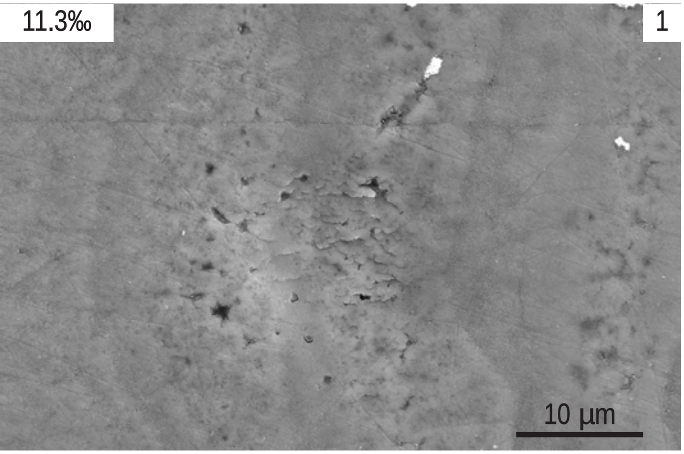

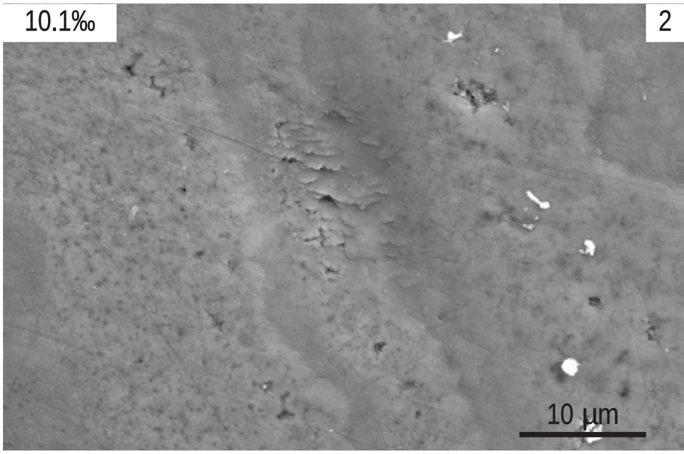

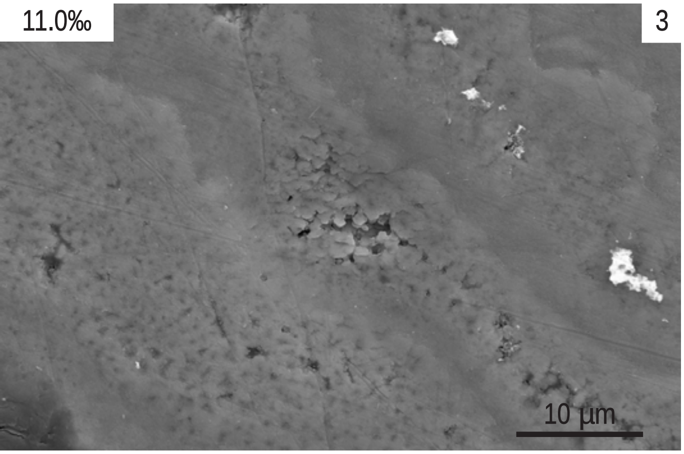

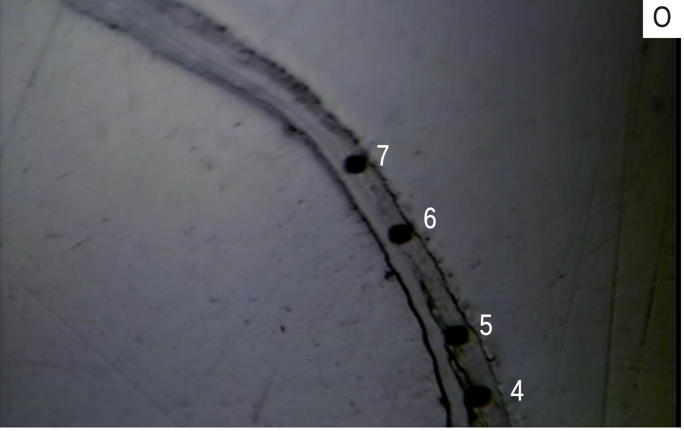

O Reflected light optical images showing locations of spots analyzed.

1–11 SEM microphotographs show recrystallized shell material, patly with fissures. Alteration of the δ18O values obtained from such portions (spots 1–7) seems to be obvious. Spots 8–11 are located within dense–fine crystalline shell material. Initial recrystallization could be observed at these spots, most probably explains the slightly more negative δ18O values.

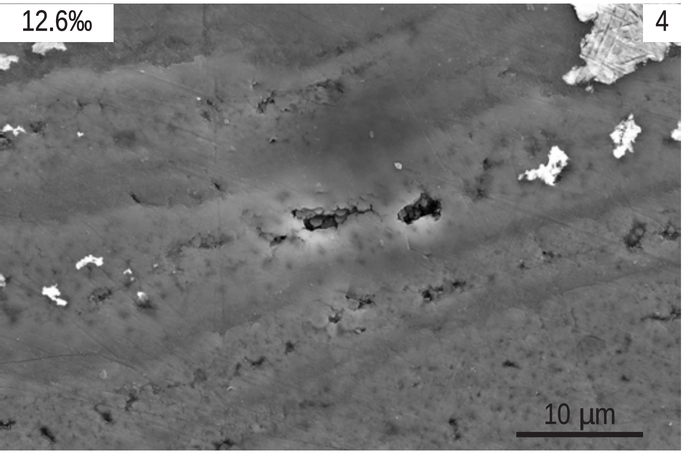

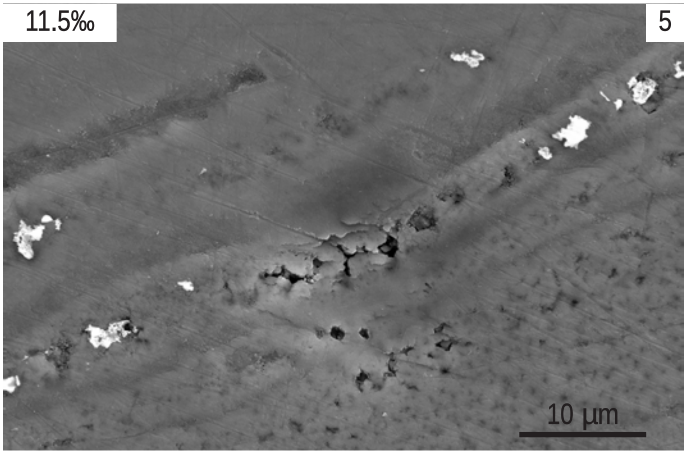

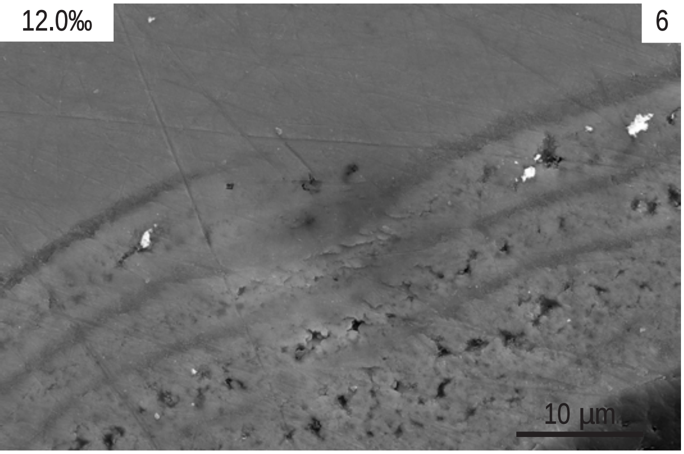

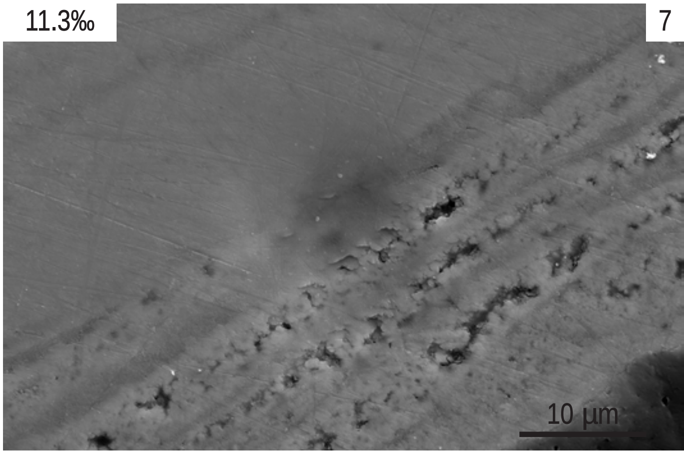

sample 1599-39

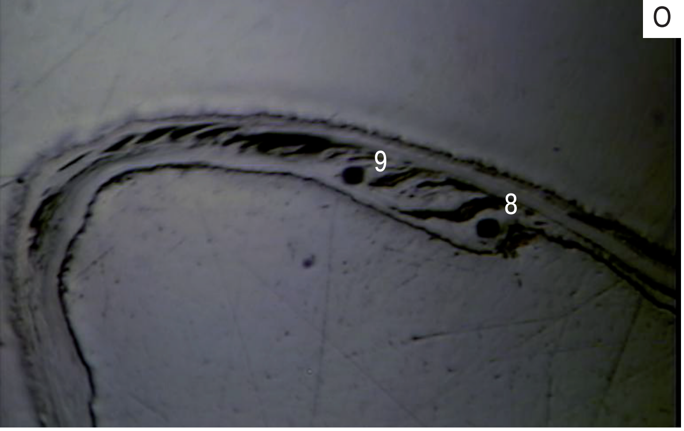

O Reflected light optical images showing locations of spots analyzed.

1–11 SEM microphotographs show recrystallized shell material, patly with fissures. Alteration of the $\delta^{18}O$ values obtained from such portions (spots 1–7) seems to be obvious. Spots 8–11 are located within dense–fine crystalline shell material. Initial recrystallization could be observed at these spots, most probably explains the slightly more negative $\delta^{18}O$ values.

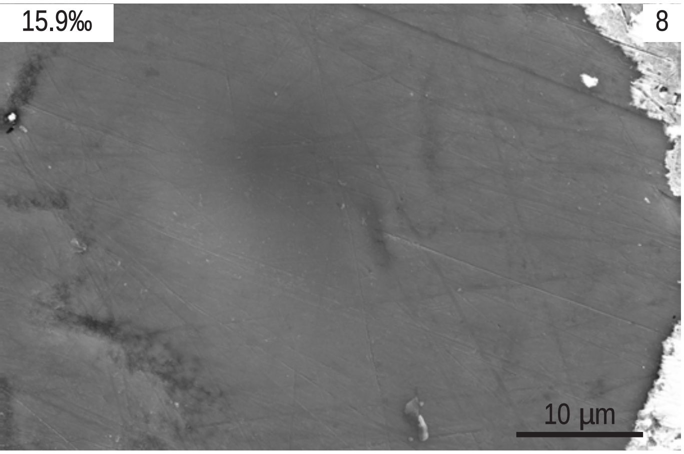

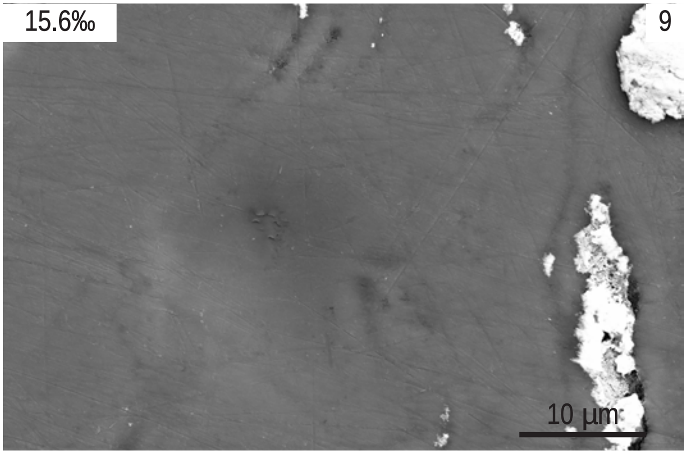

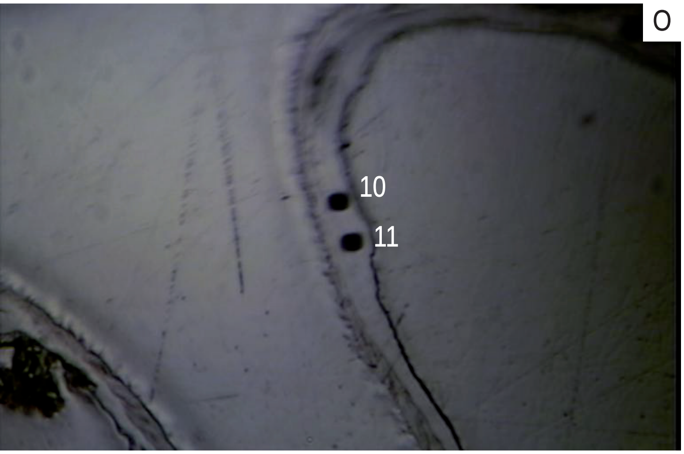

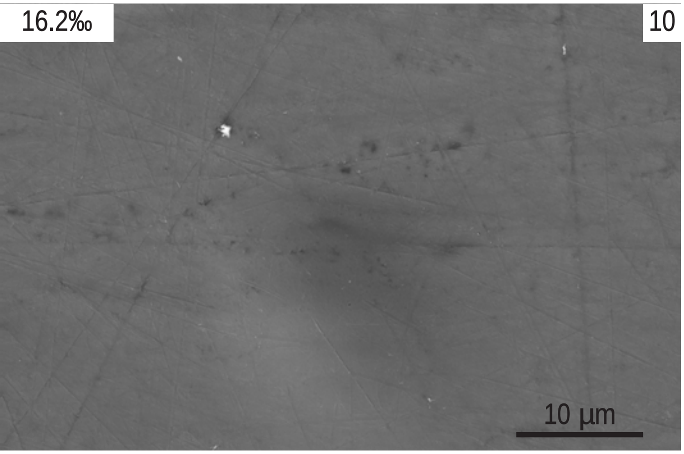

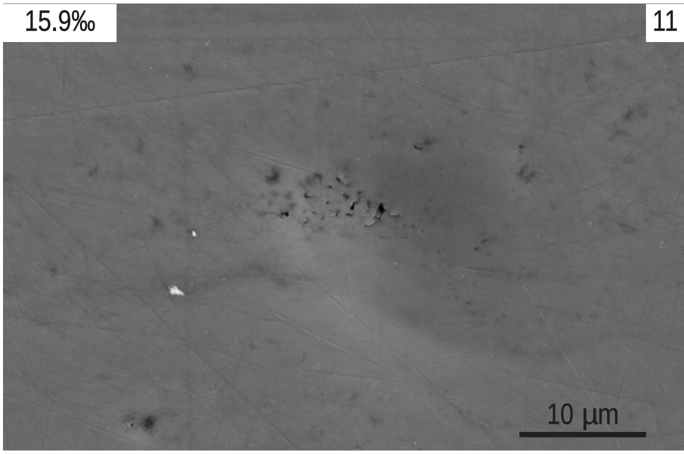

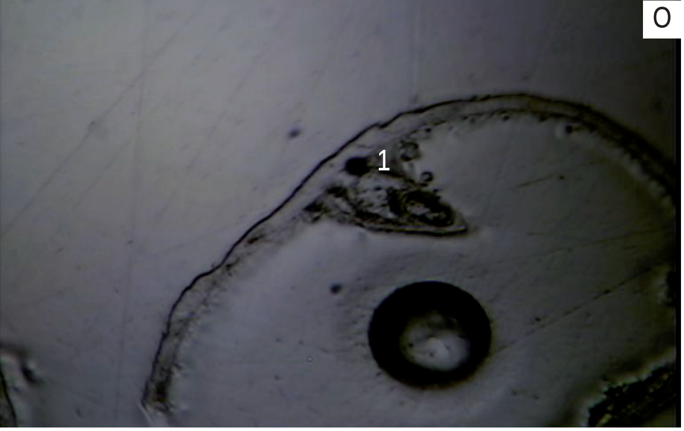

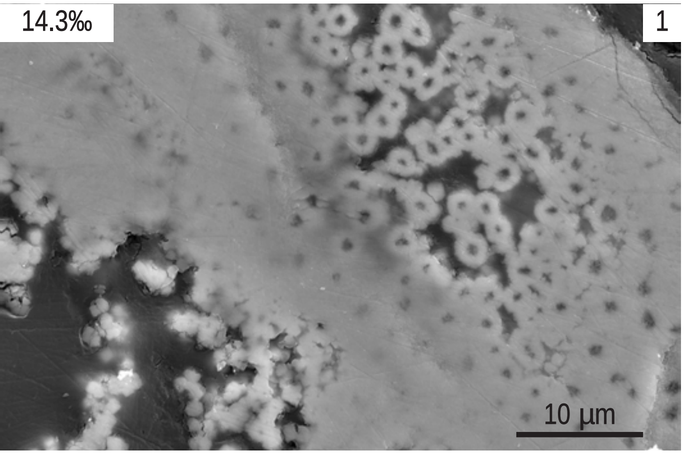

O Reflected light optical image showing the location of the analyzed spot.

1 SEM microphotograph shows recrystallized shell material. An alteration of the $\delta^{18}O$ values seems to be obvious.

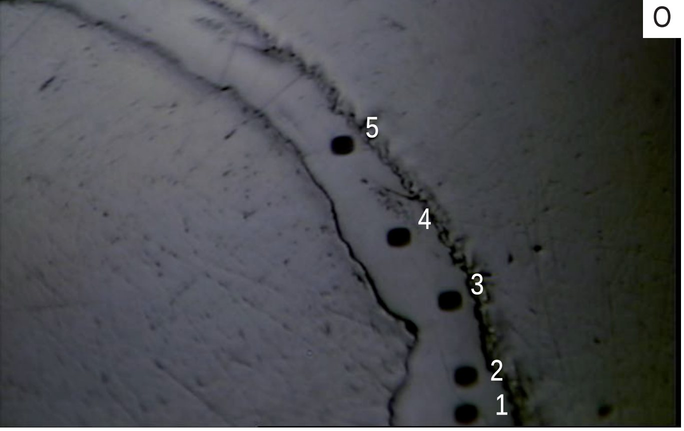

O Reflected light optical images showing locations of spots analyzed.

1–14 SEM microphotographs show dense crystalline shell material, mostly without any indication of recrystallization. Slightly lighter $\delta^{18}O$ values at spots 5, 9, and 12 (15.8‰, 16.5‰, and 15.7‰) most probably reflect an impact of a fissure (spot 5) and initial recrystallization (spots 9 & 12). Fissures and pores are potential pathways for fluid migration.

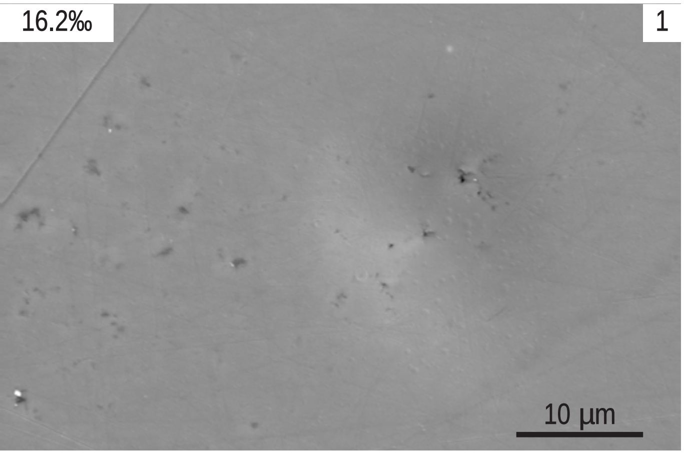

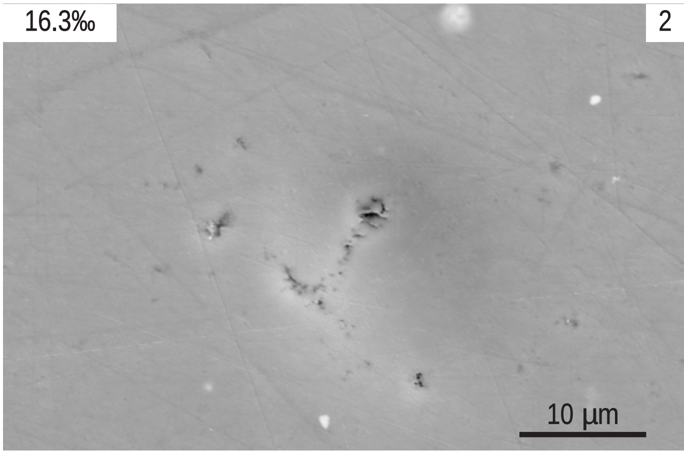

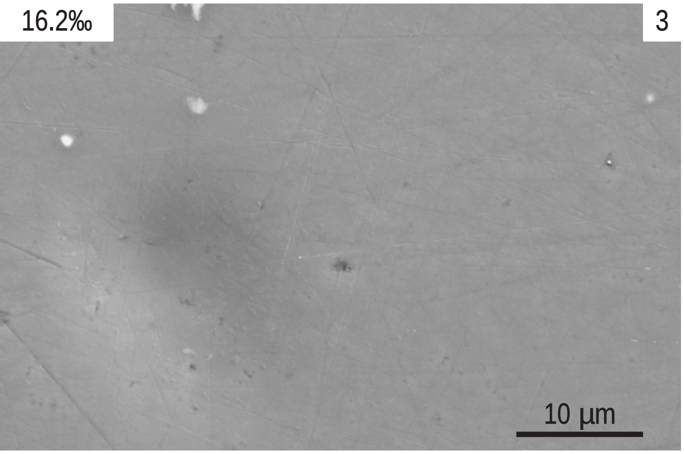

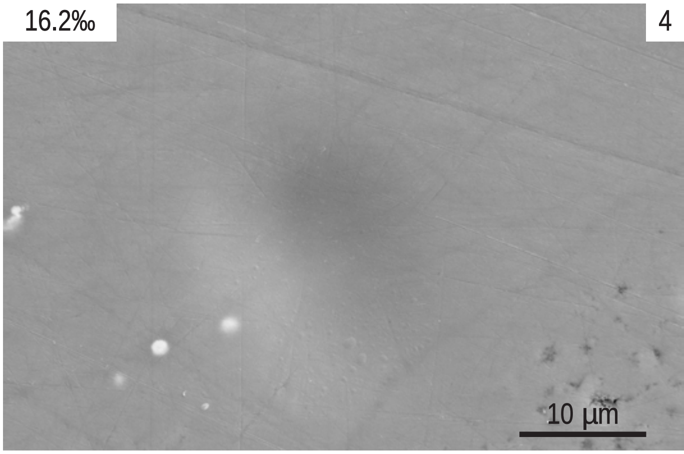

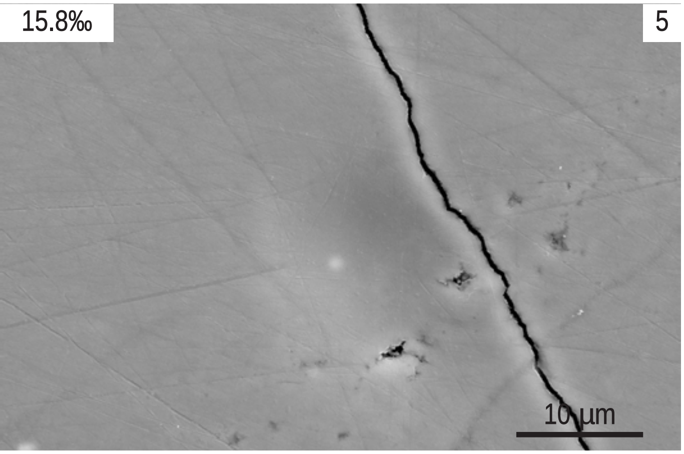

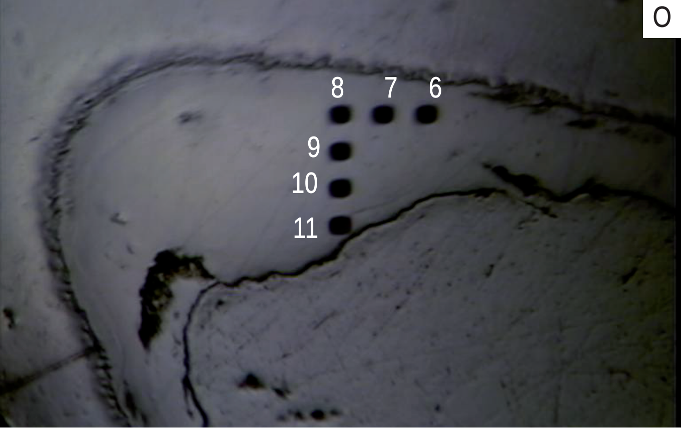

O Reflected light optical images showing locations of spots analyzed.

1–14 SEM microphotographs show dense crystalline shell material, mostly without any indication of recrystallization. Slightly lighter $\delta^{18}O$ values at spots 5, 9, and 12 (15.8‰, 16.5‰, and 15.7‰) most probably reflect an impact of a fissure (spot 5) and initial recrystallization (spots 9 & 12). Fissures and pores are potential pathways for fluid migration.

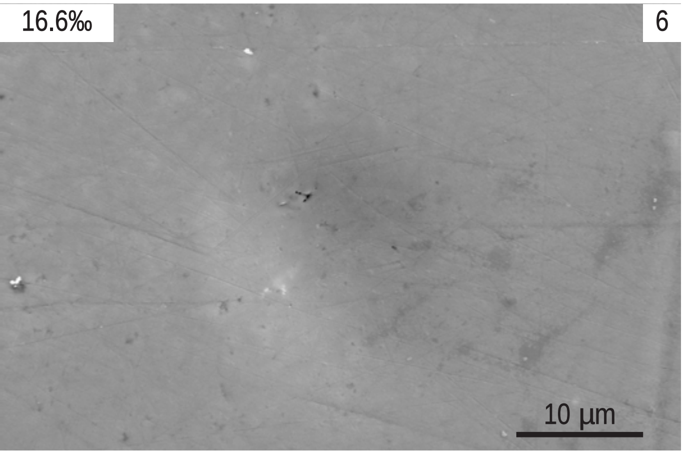

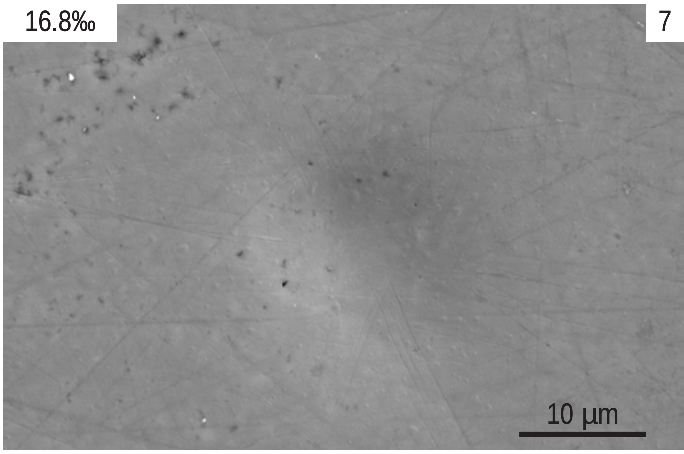

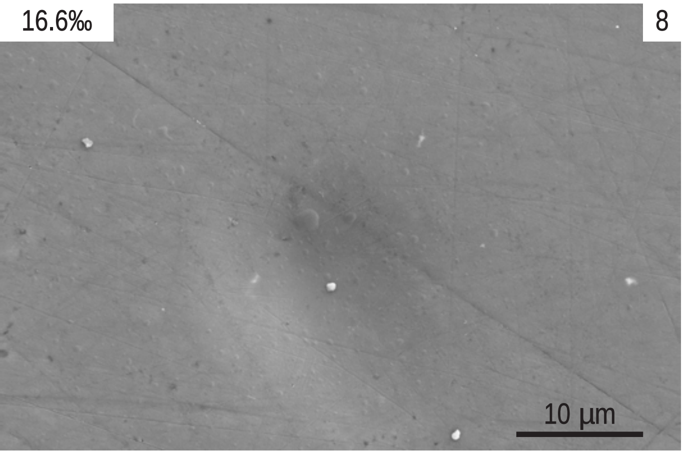

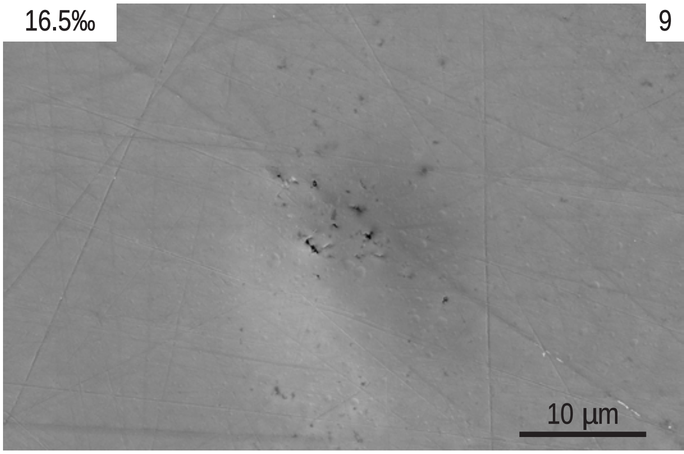

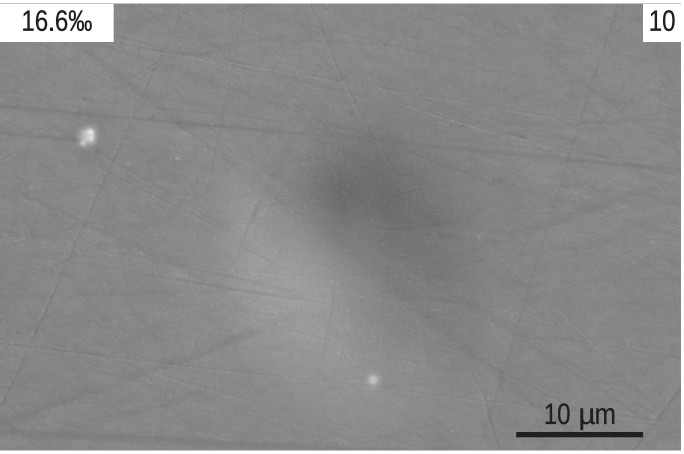

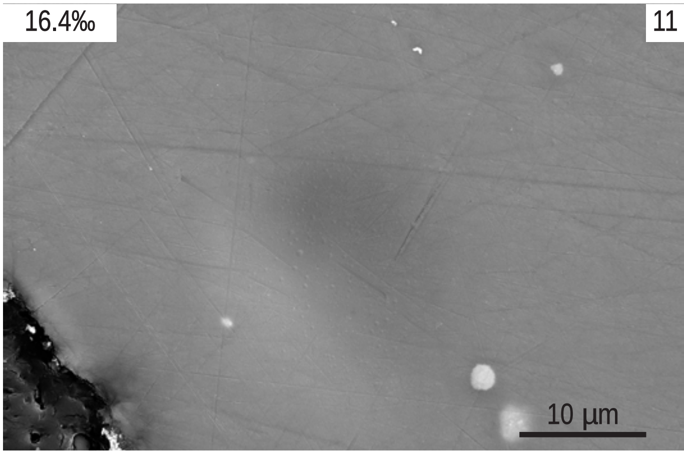

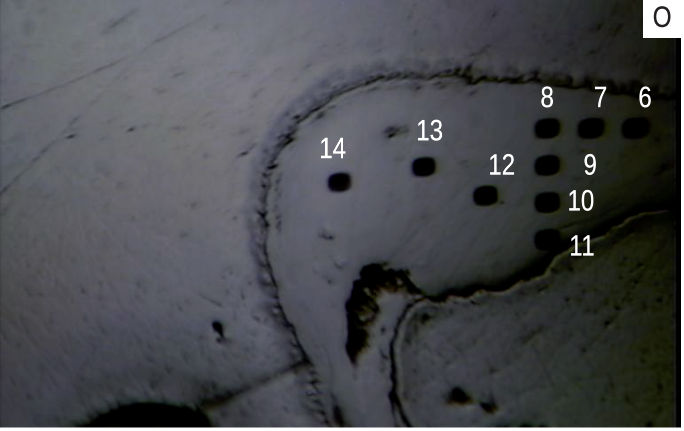

O Reflected light optical images showing locations of spots analyzed.

1–14 SEM microphotographs show dense crystalline shell material, mostly without any indication of recrystallization. Slightly lighter δ^{18}O values at spots 5, 9, and 12 (15.8‰, 16.5‰, and 15.7‰) most probably reflect an impact of a fissure (spot 5) and initial recrystallization (spots 9 & 12). Fissures and pores are potential pathways for fluid migration.

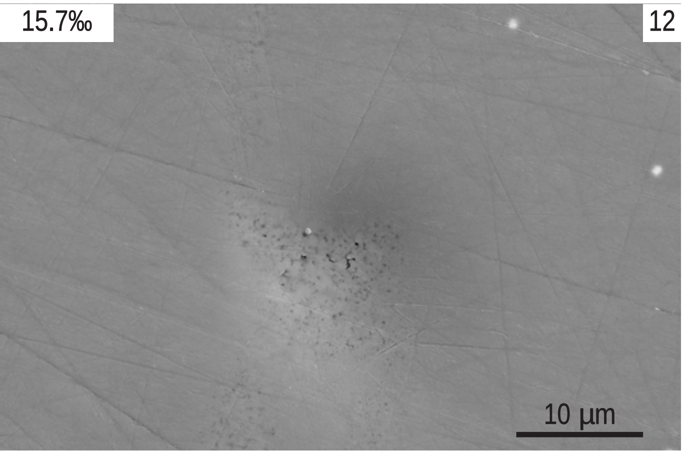

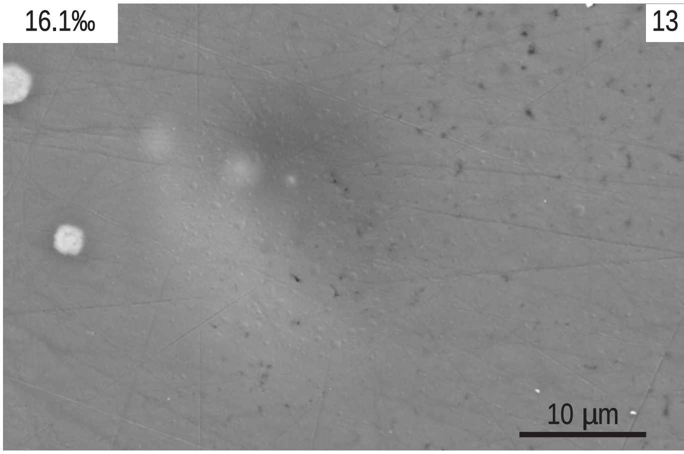

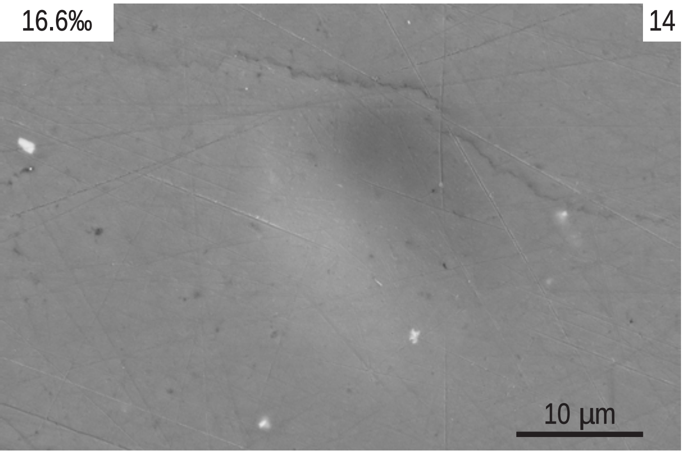

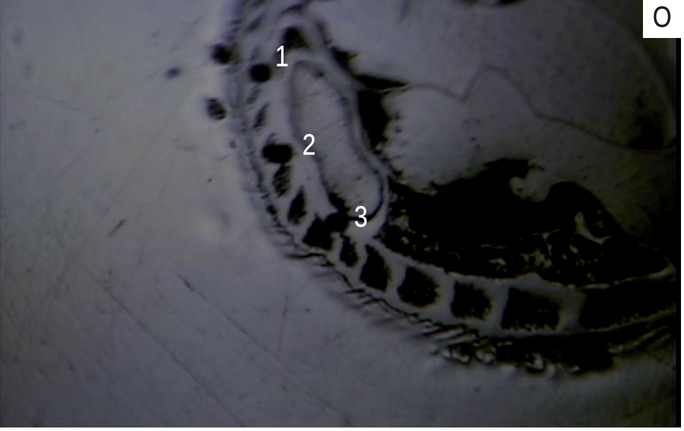

O Reflected light optical images showing locations of spots analyzed.

1–6 SEM microphotographs show dense crystalline shell material surrounded by recrystallized parts. Initial recrystallization at spot 3 is consistent with a slightly more negative $\delta^{18}O$ value.

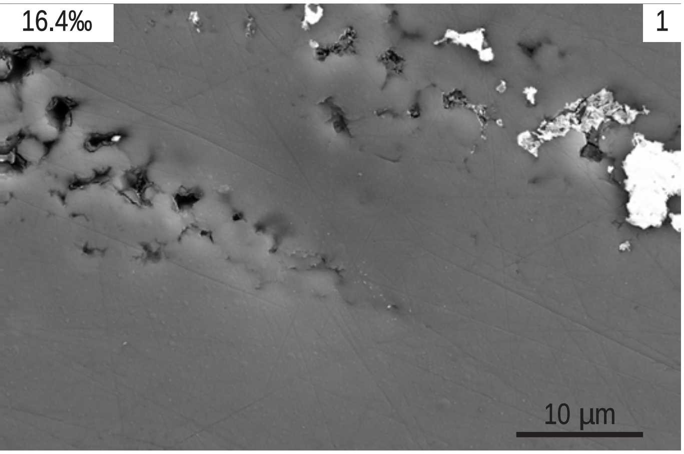

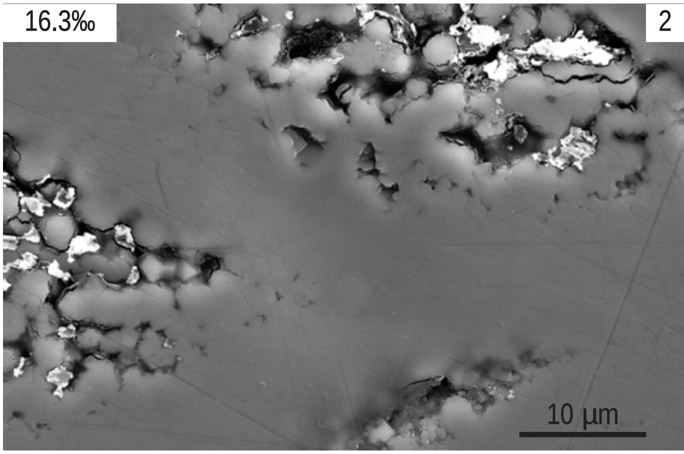

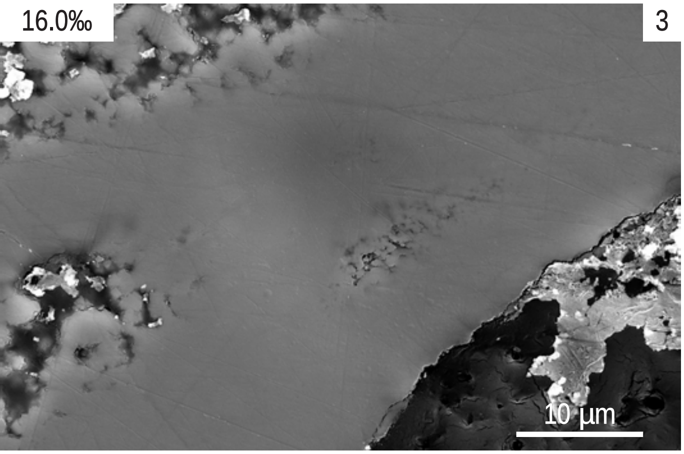

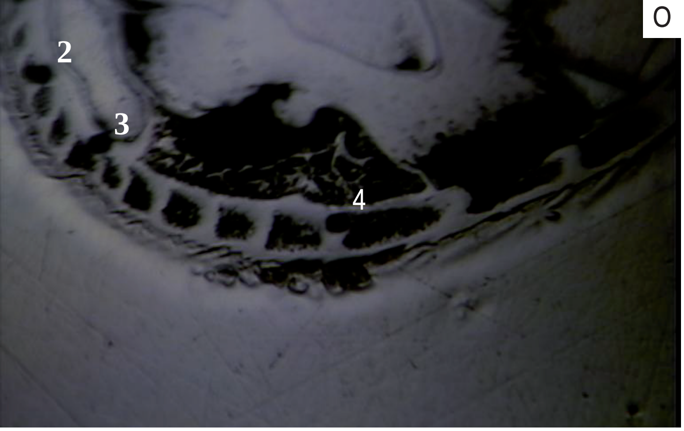

O Reflected light optical images showing locations of spots analyzed.

1–6 SEM microphotographs show dense crystalline shell material surrounded by recrystallized parts. Initial recrystallization at spot 3 is consistent with a slightly more negative $\delta^{18}O$ value.

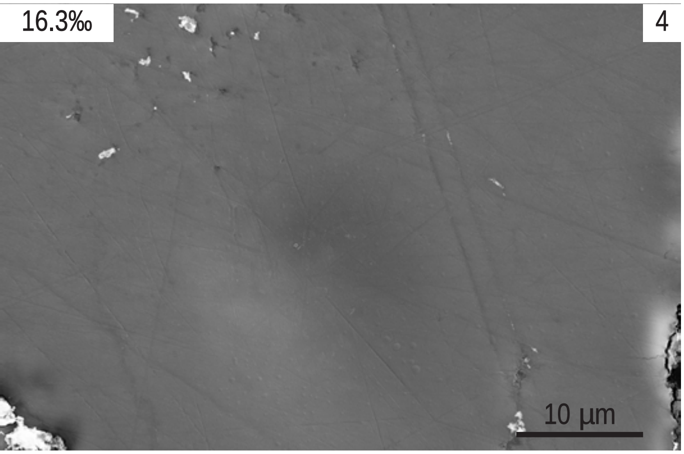

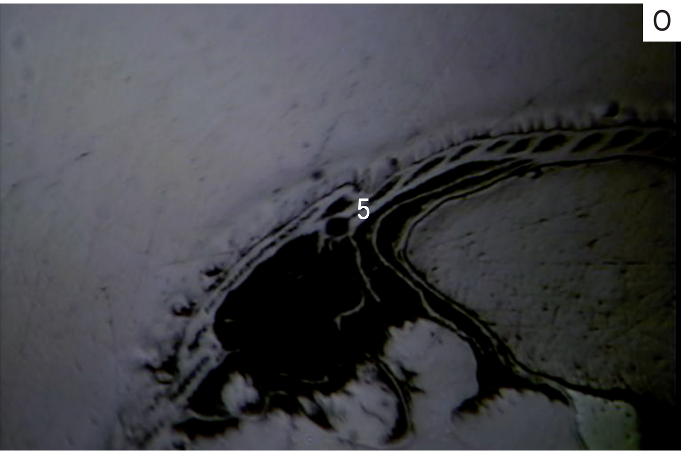

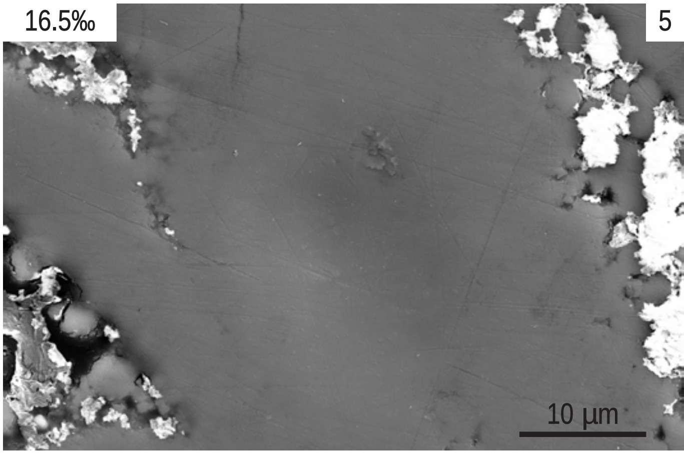

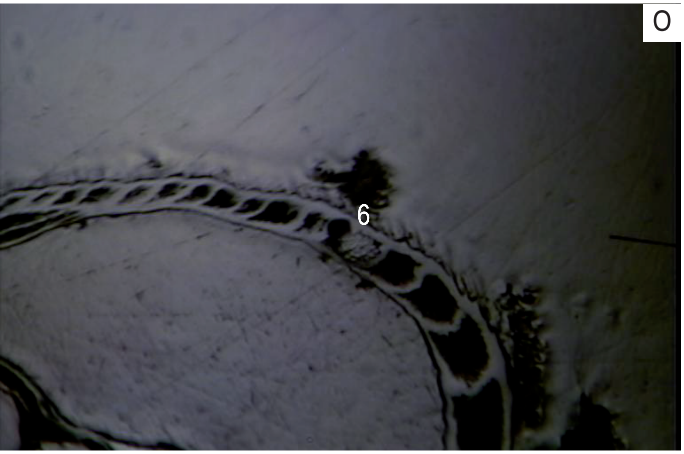

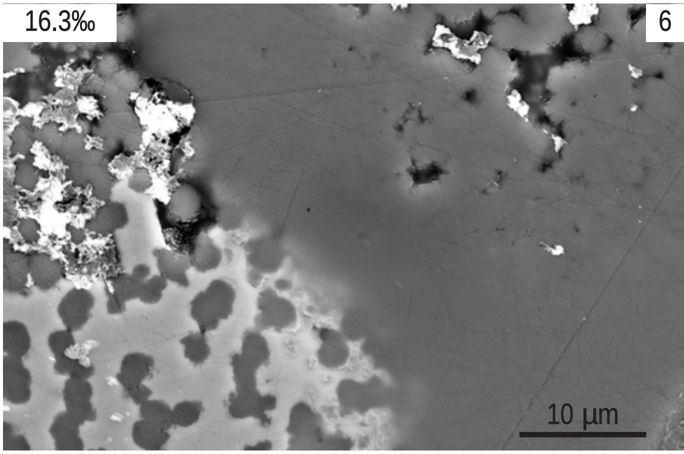

Supplementary Material 3. SEM- and SEM-EDS microphotographs of selected spots. No indication for changes in element concentrations could be observed.

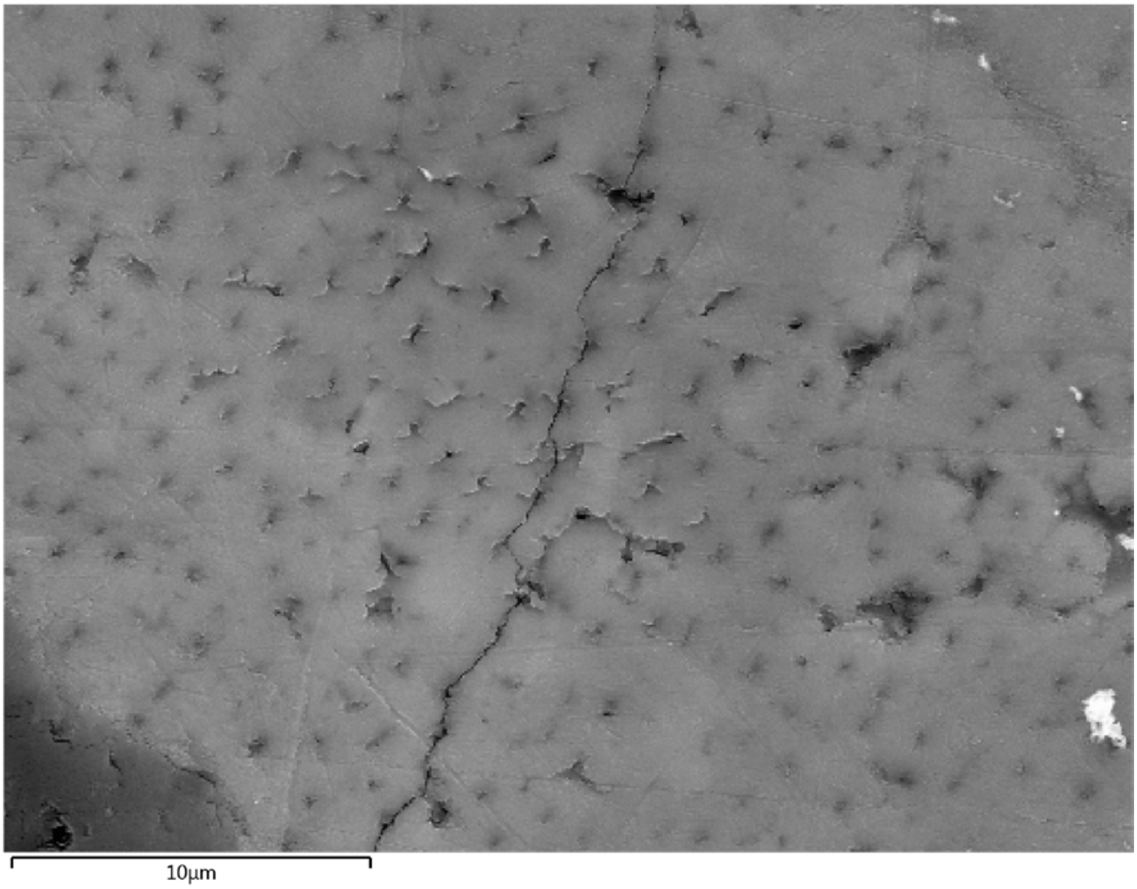

SEM image

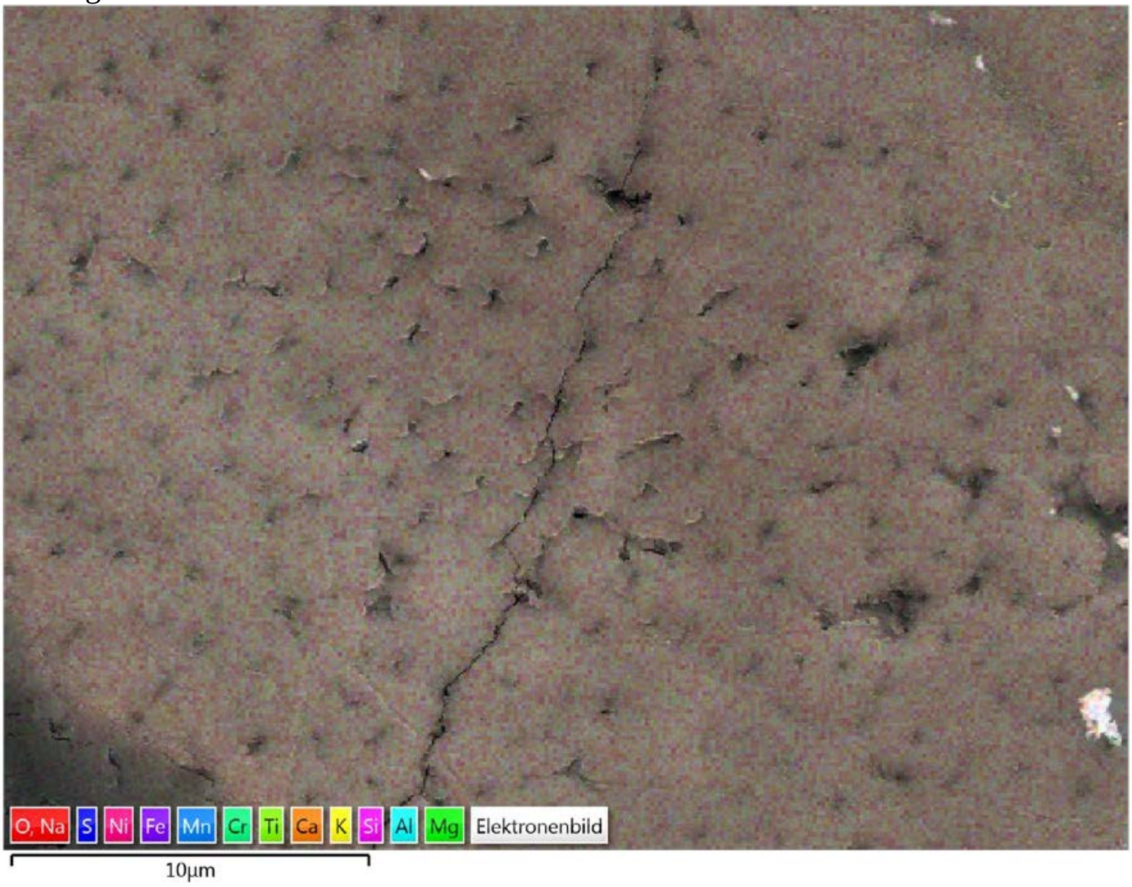

Overlay SEM-EDS image

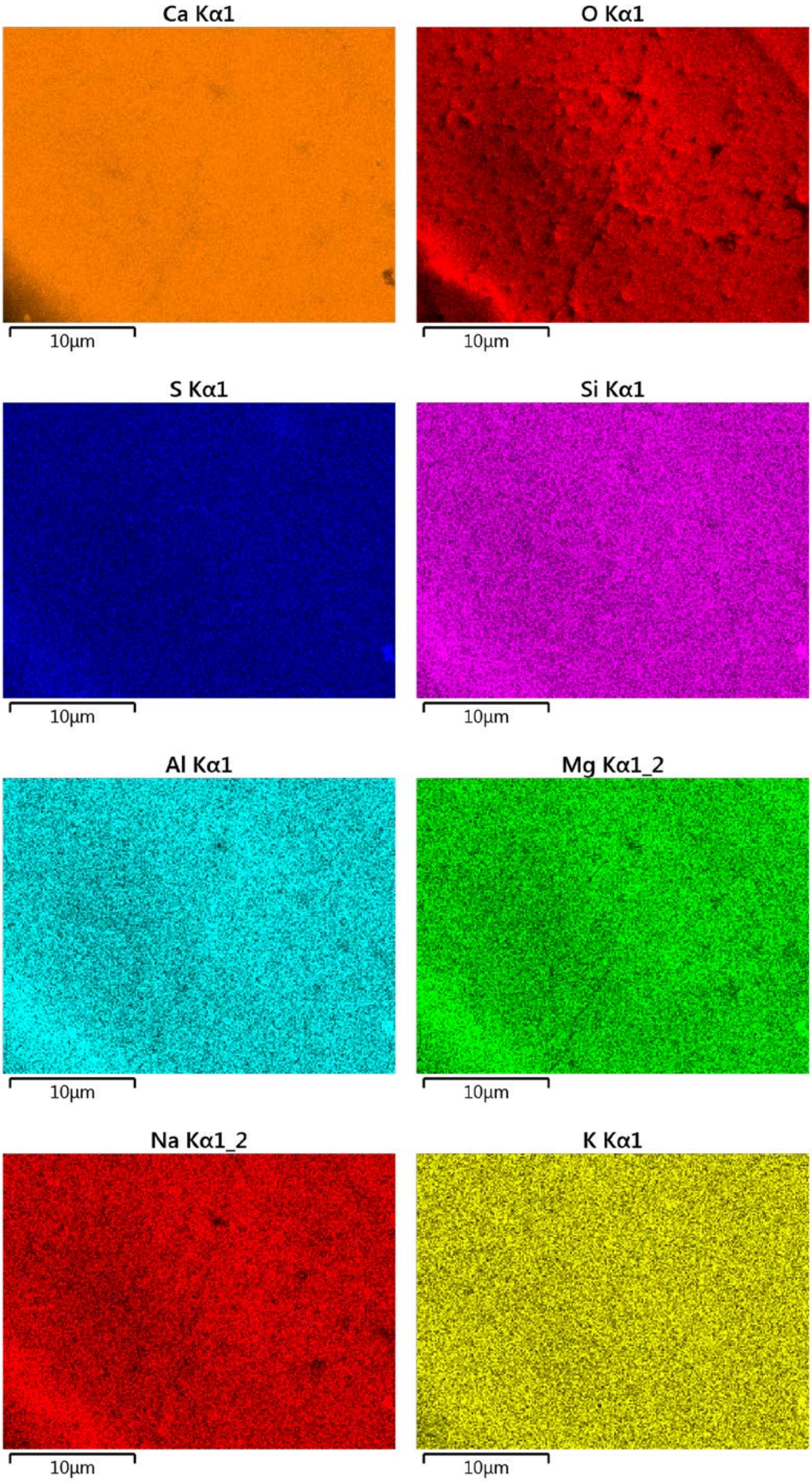
Ca Kα1
10μm
O Kα1
10μm
S Kα1
10μm
Si Kα1
10μm
Al Kα1
10μm
Mg Kα1_2
10μm
Na Kα1_2
10μm
K Kα1
10μm

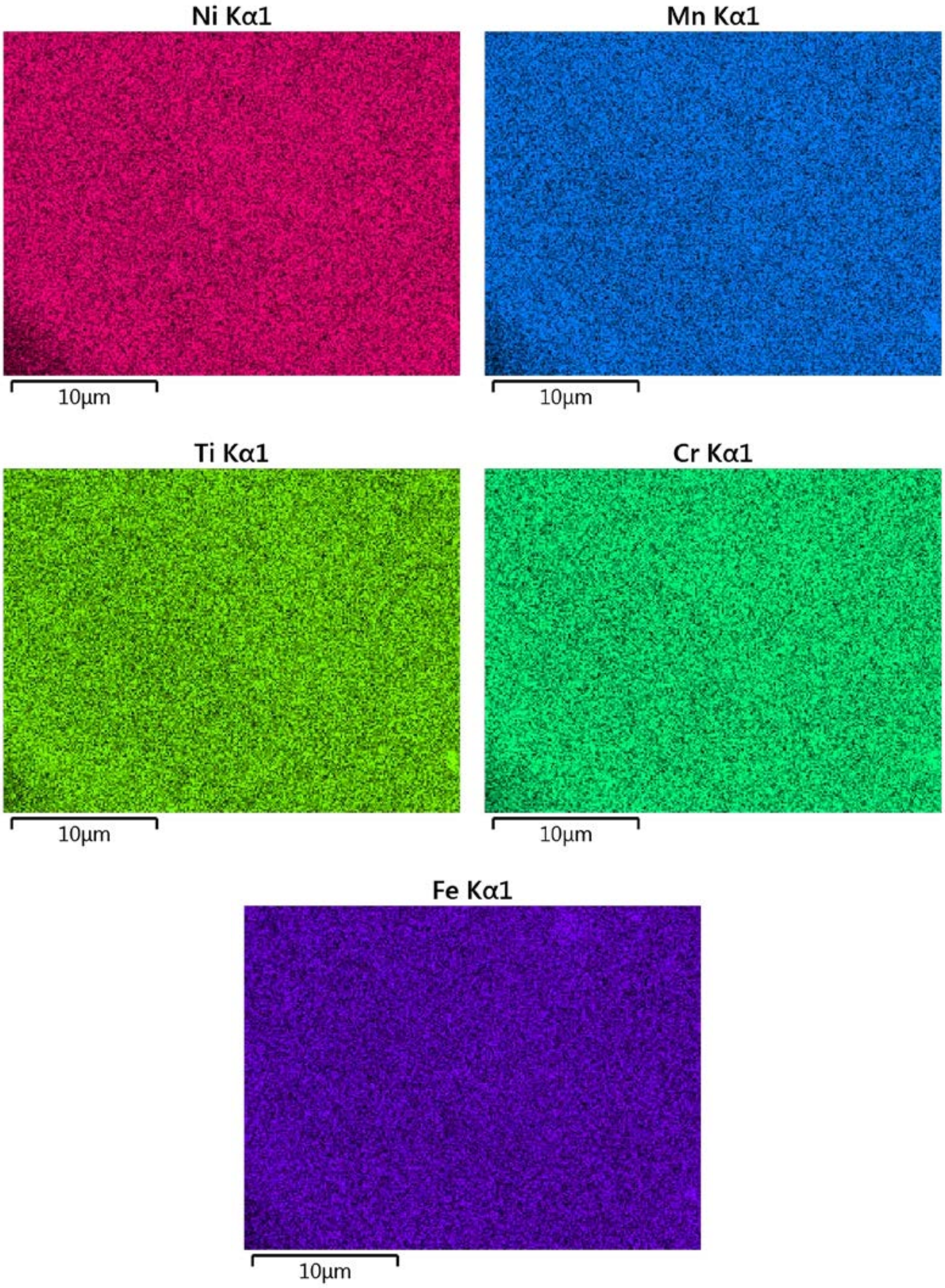
Ni Kα1
10μm
Mn Kα1
10μm
Ti Kα1
10μm
Cr Kα1
10μm
Fe Kα1
10μm

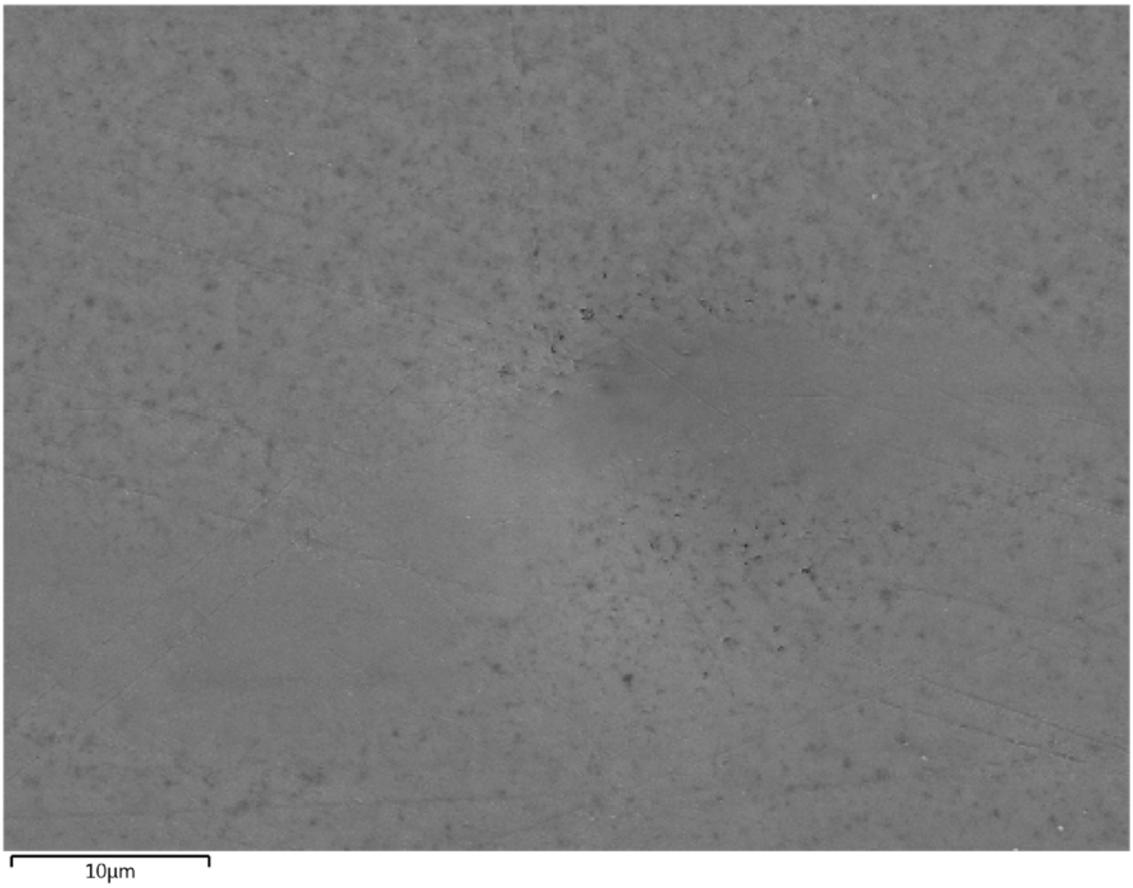

SEM image

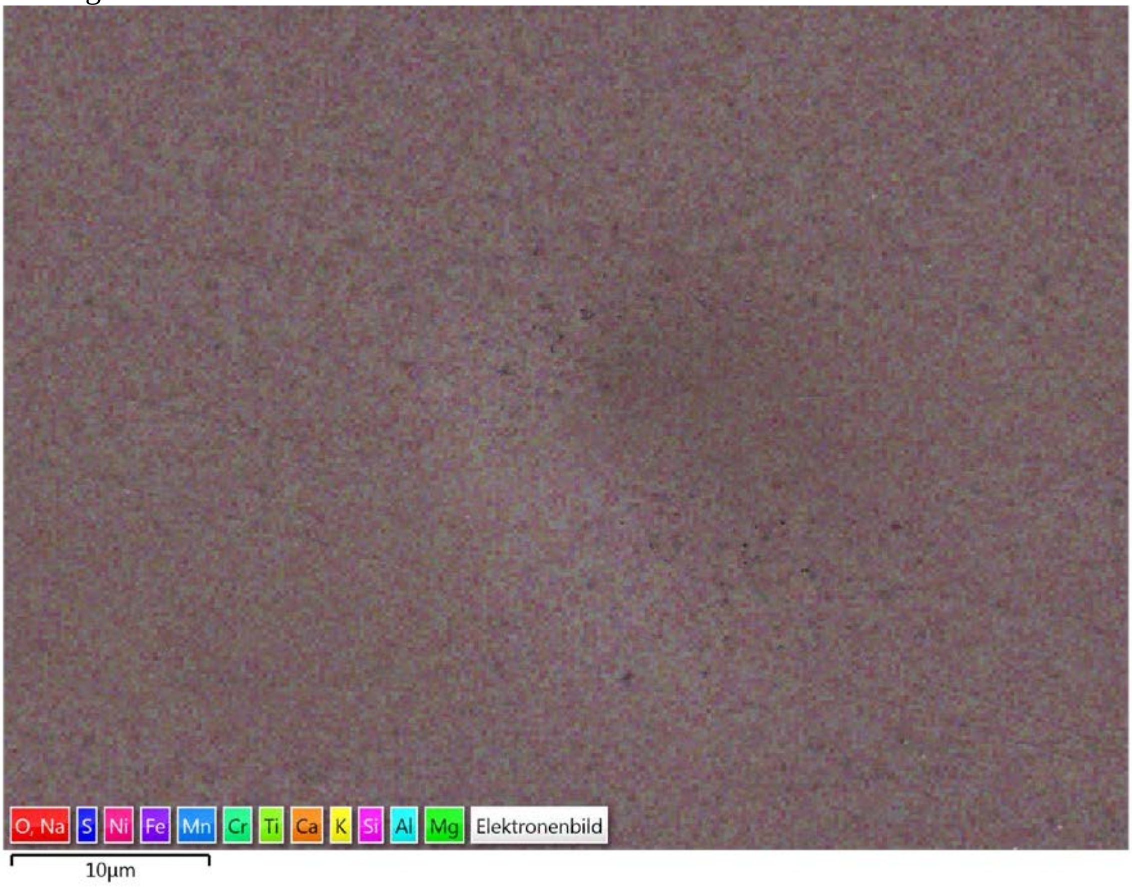

Overlay SEM-EDS image

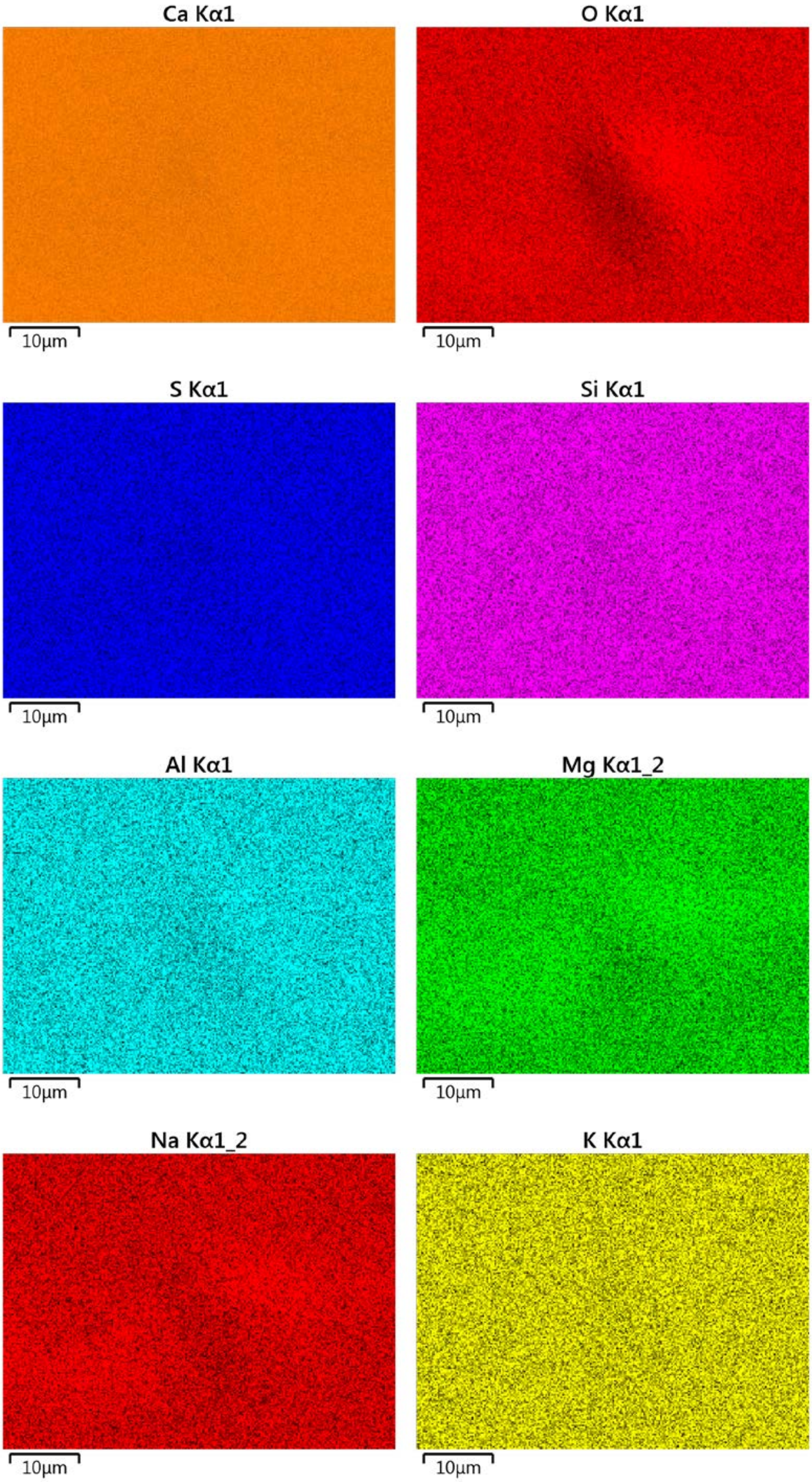
Ca Kα1
10μm
O Kα1
10μm
S Kα1
10μm
Si Kα1
10μm
Al Kα1
10μm
Mg Kα1_2
10μm
Na Kα1_2
10μm
K Kα1
10μm

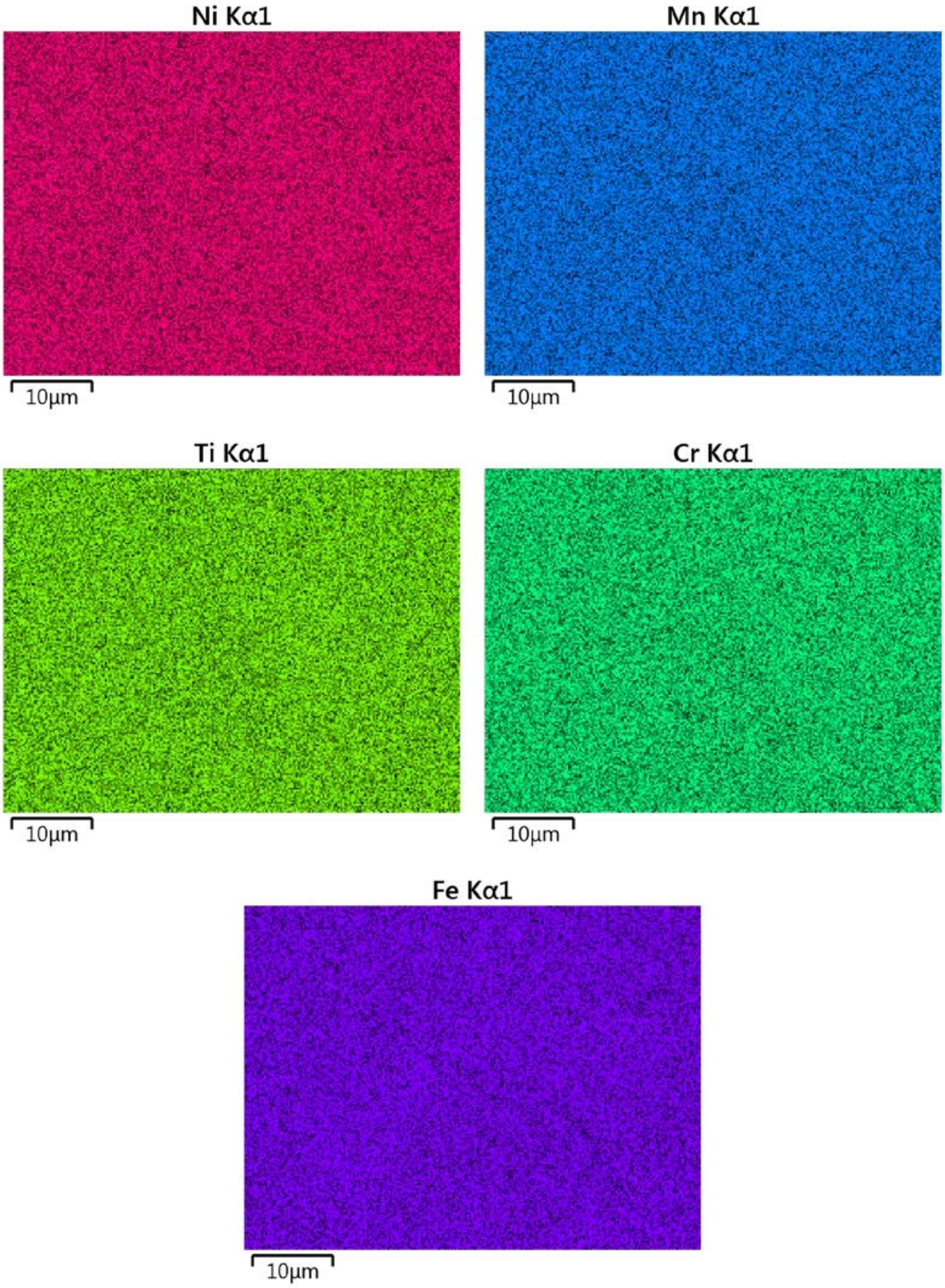
Ni Kα1
10μm
Mn Kα1
10μm
Ti Kα1
10μm
Cr Kα1
10μm
Fe Kα1
10μm

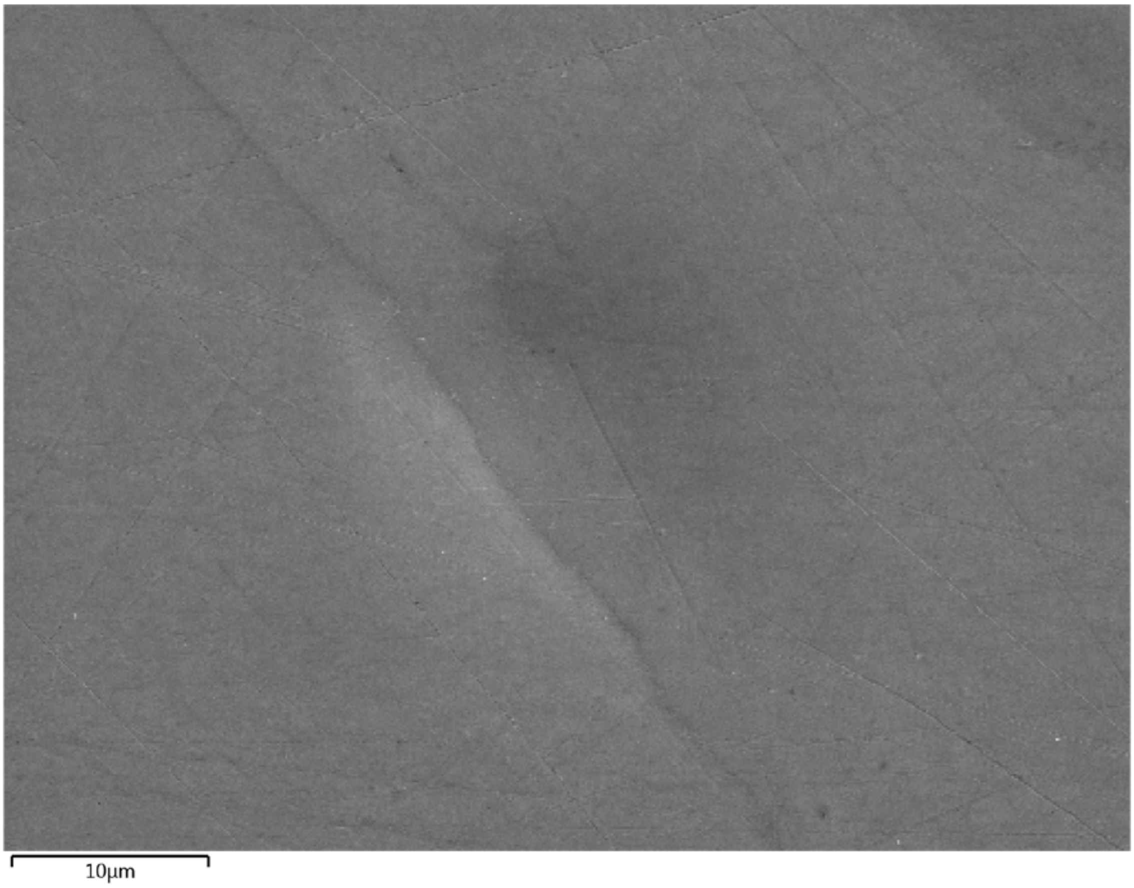

SEM image

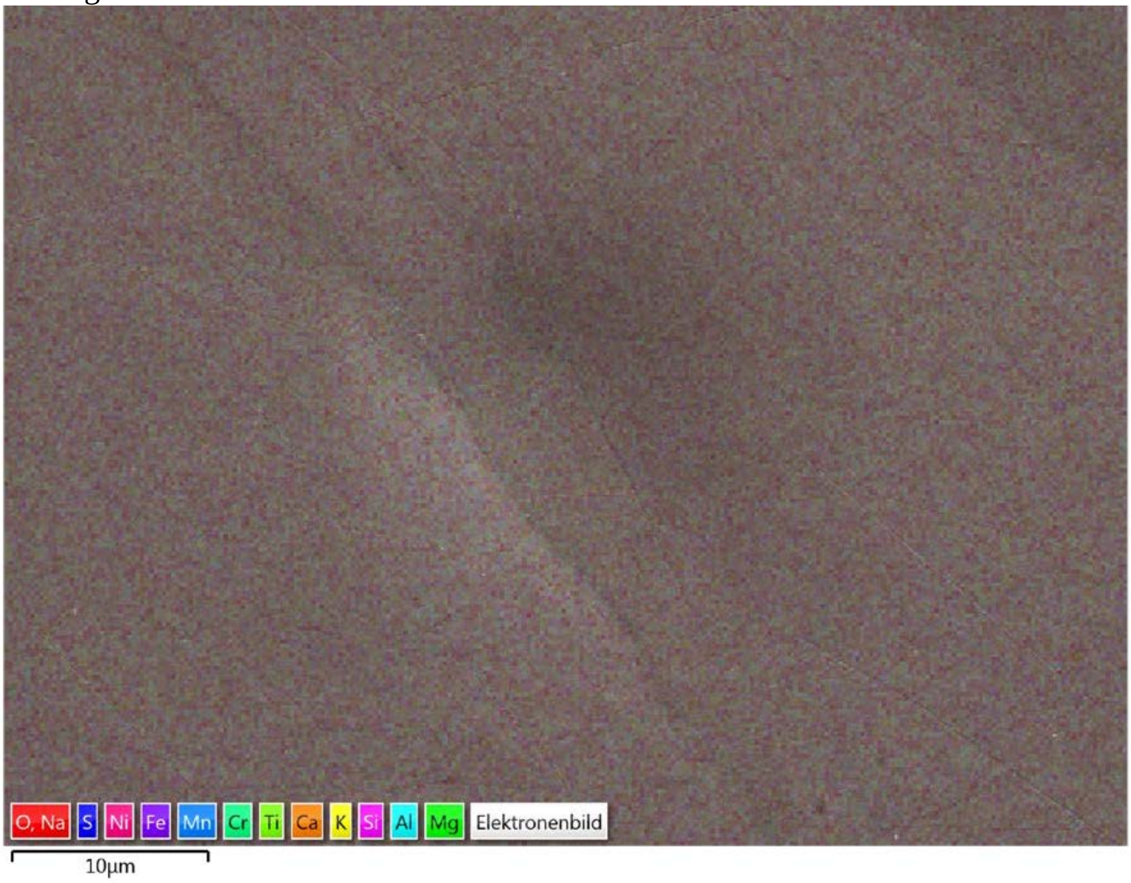

Overlay SEM-EDS image

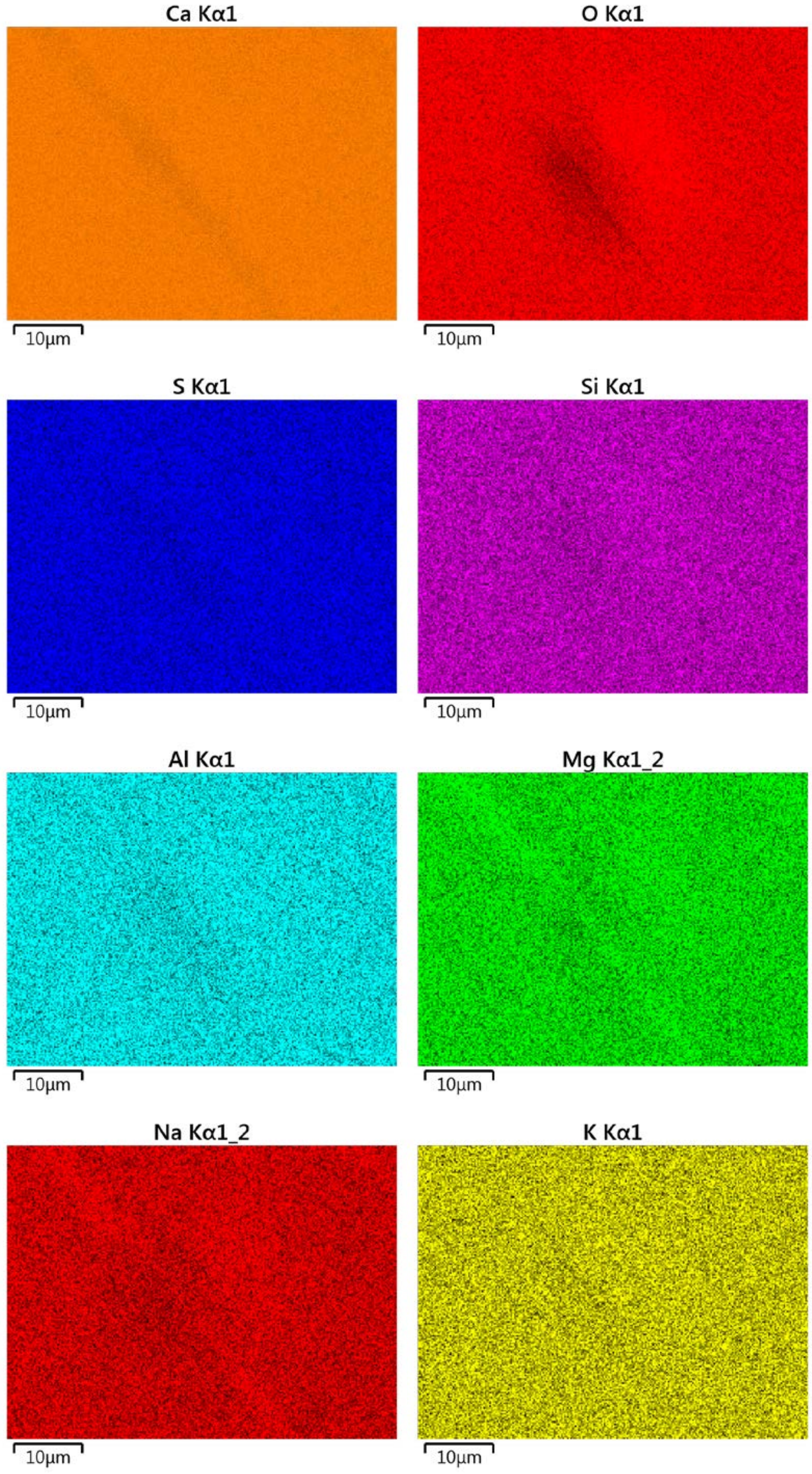
Ca Kα1
10μm
O Kα1
10μm
S Kα1
10μm
Si Kα1
10μm
Al Kα1
10μm
Mg Kα1_2
10μm
Na Kα1_2
10μm
K Kα1
10μm

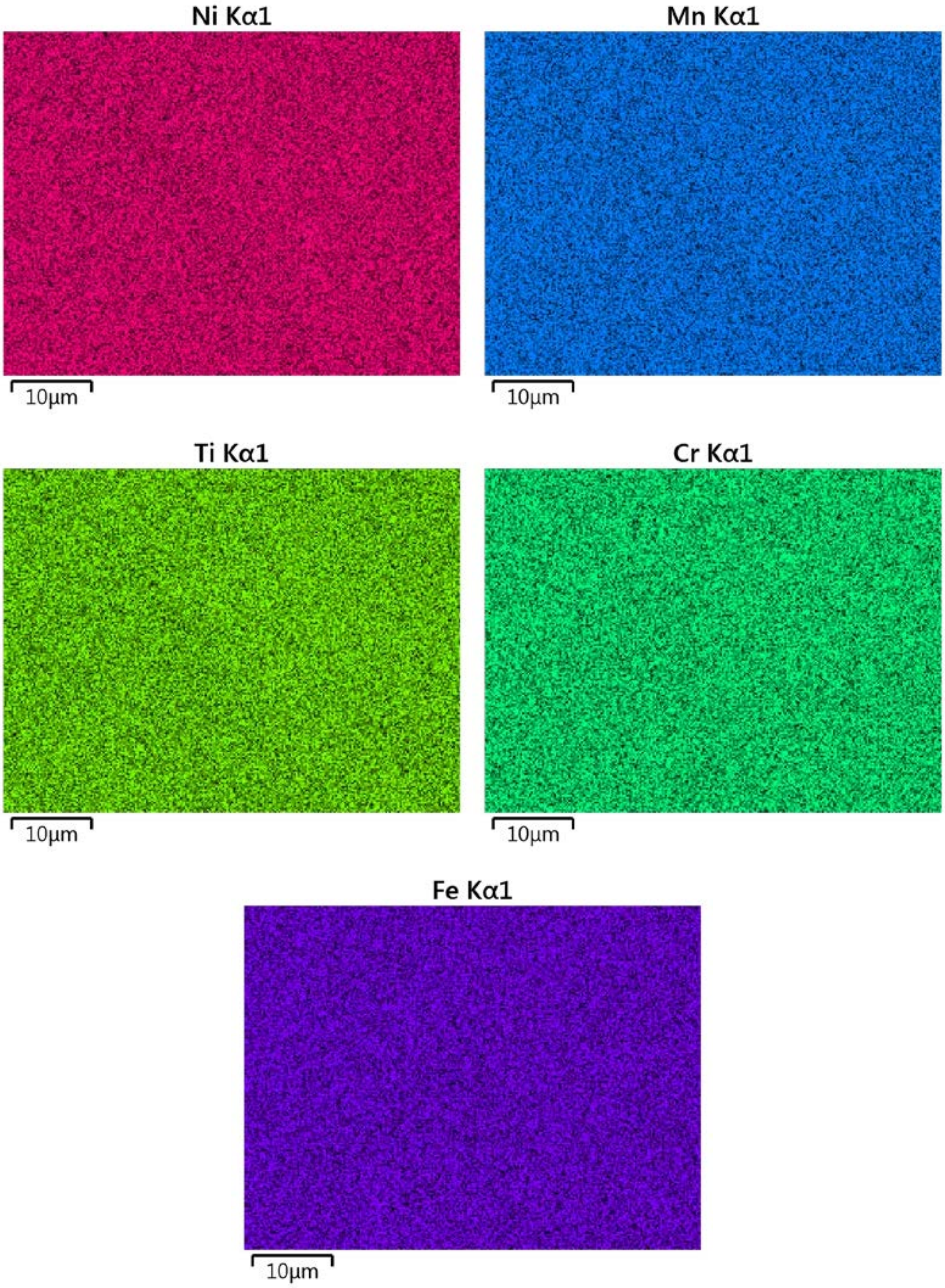
Ni Kα1
Mn Kα1
10μm
10μm
Ti Kα1
Cr Kα1
10μm
10μm
Fe Kα1
10μm

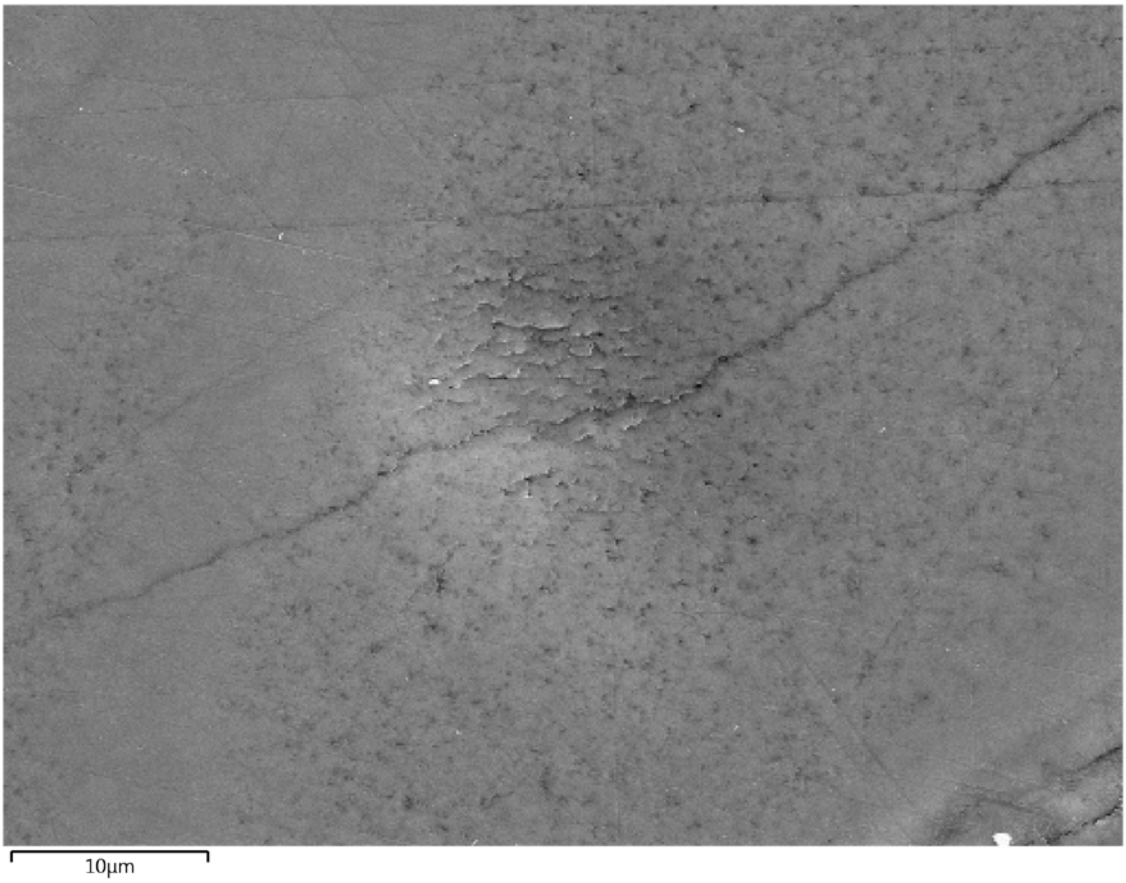

SEM image

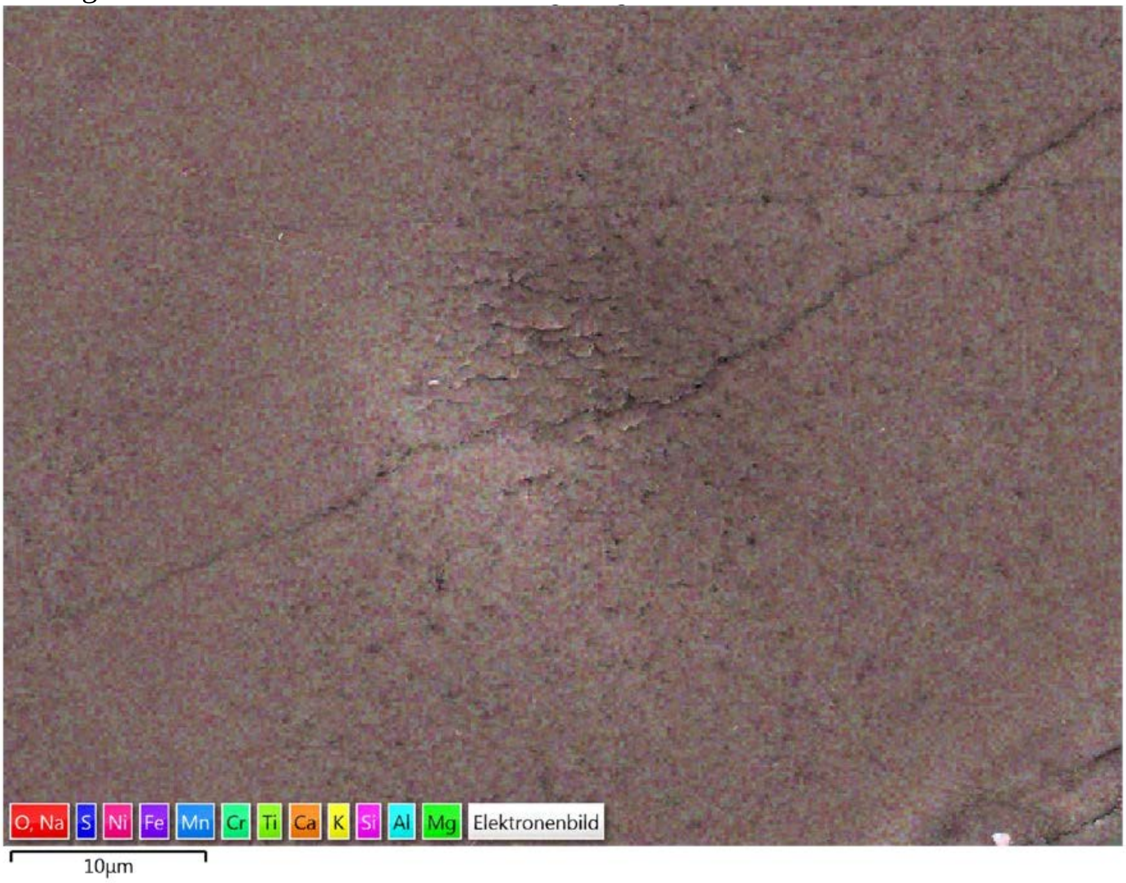

Overlay SEM-EDS image

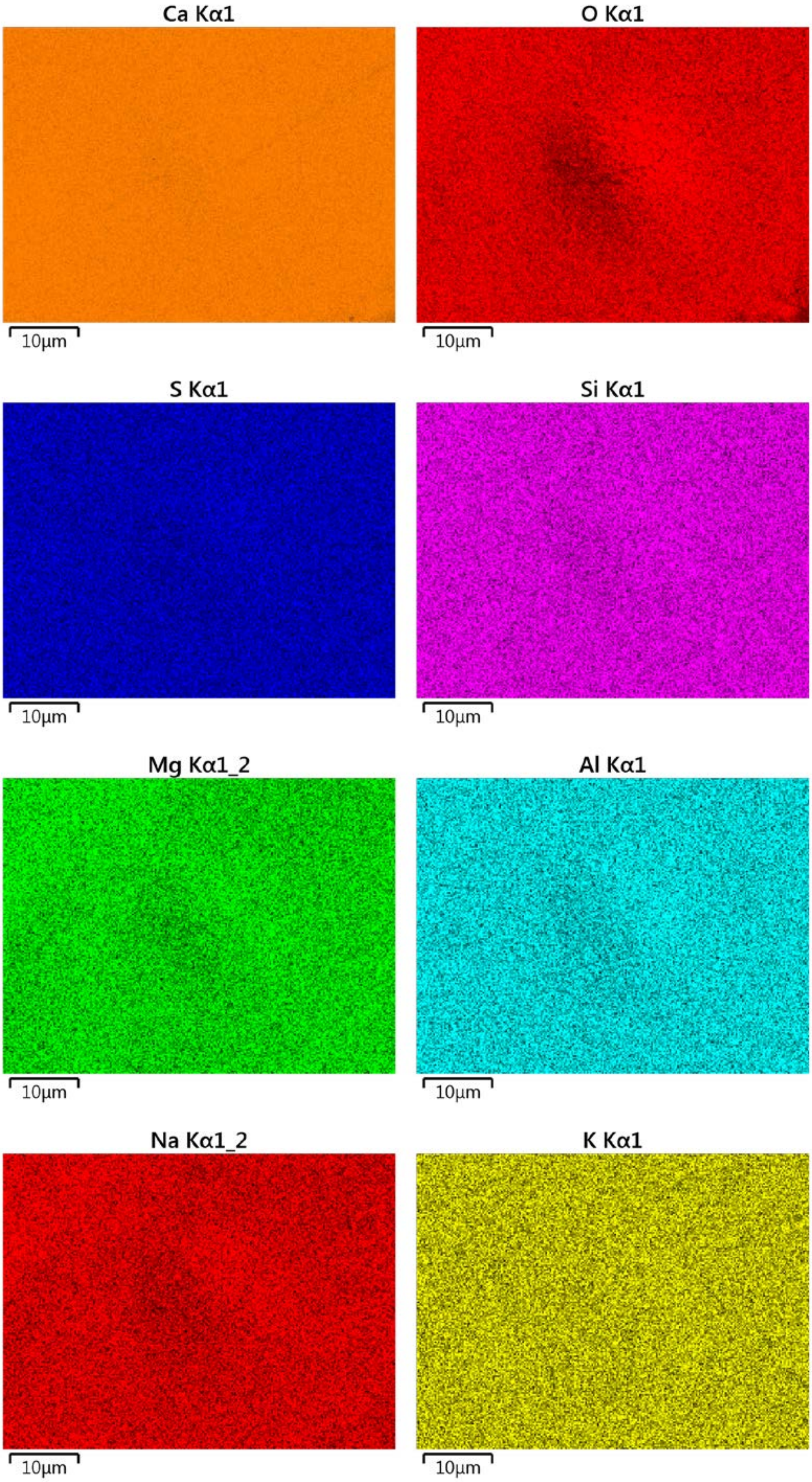
Ca Kα1
10µm
O Kα1
10µm
S Kα1
10µm
Si Kα1
10µm
Mg Kα1_2
10µm
Al Kα1
10µm
Na Kα1_2
10µm
K Kα1
10µm

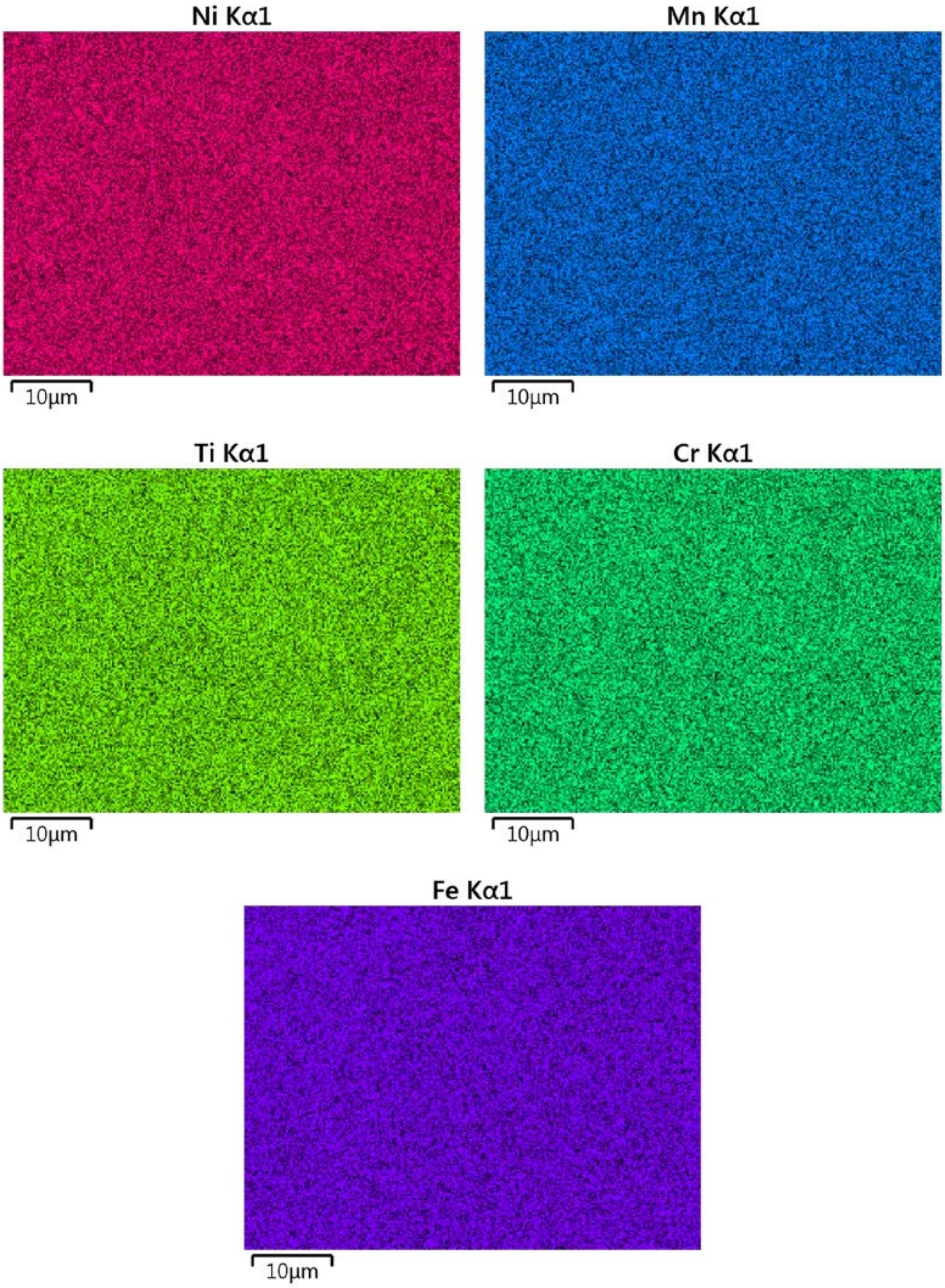
Ni Kα1
10μm
Mn Kα1
10μm
Ti Kα1
10μm
Cr Kα1
10μm
Fe Kα1
10μm

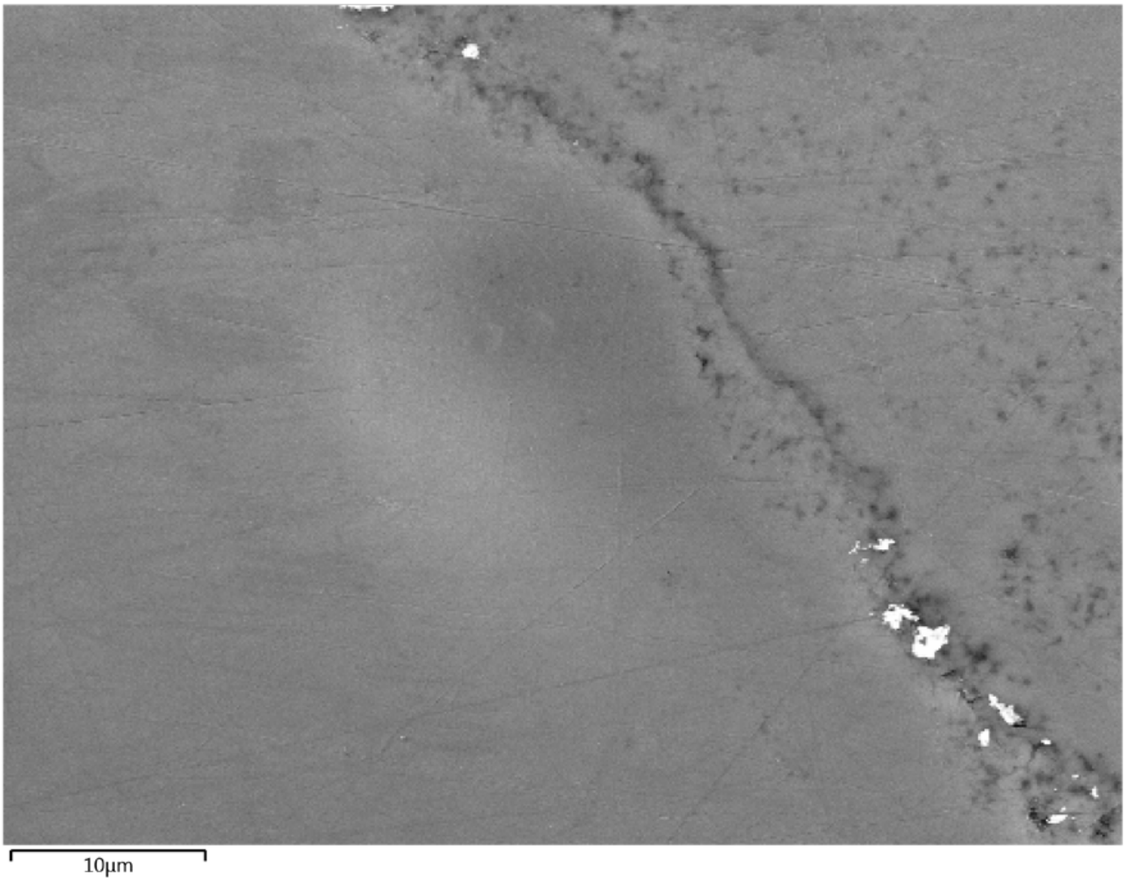

SEM image

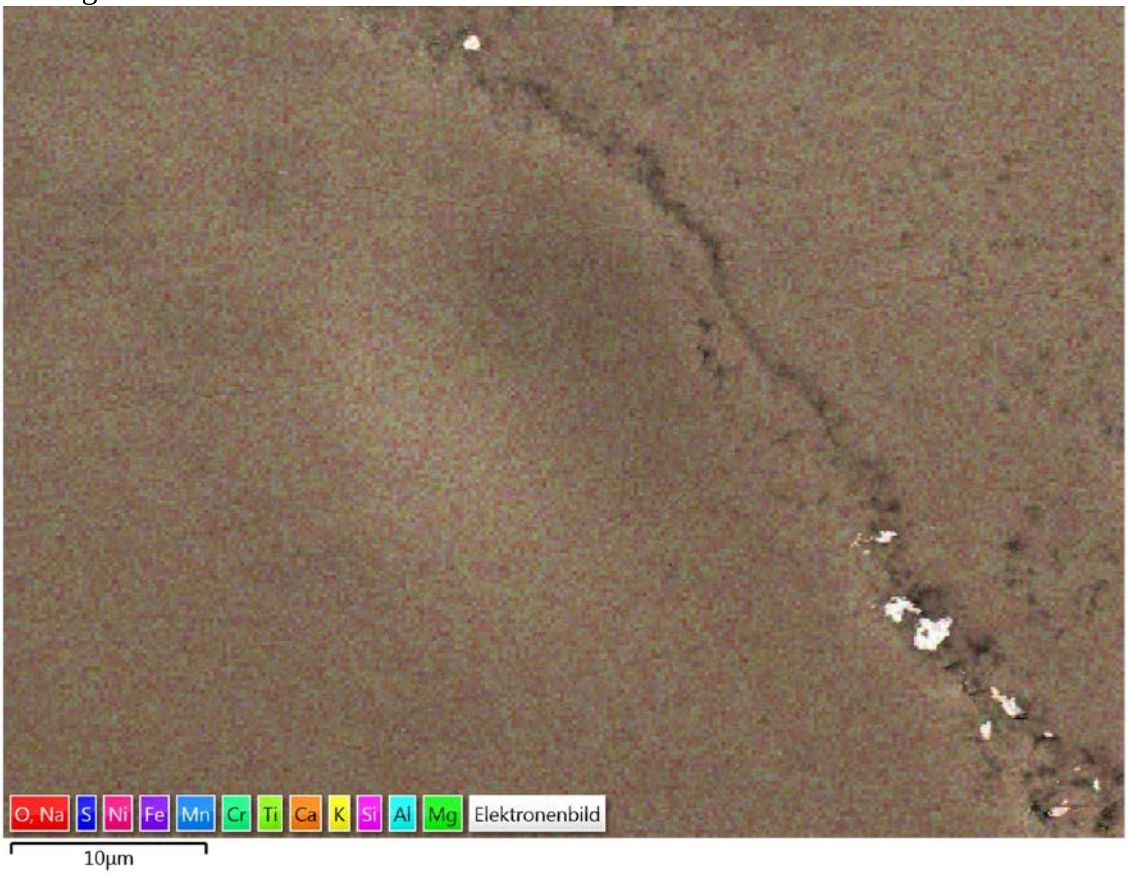

Overlay SEM-EDS image

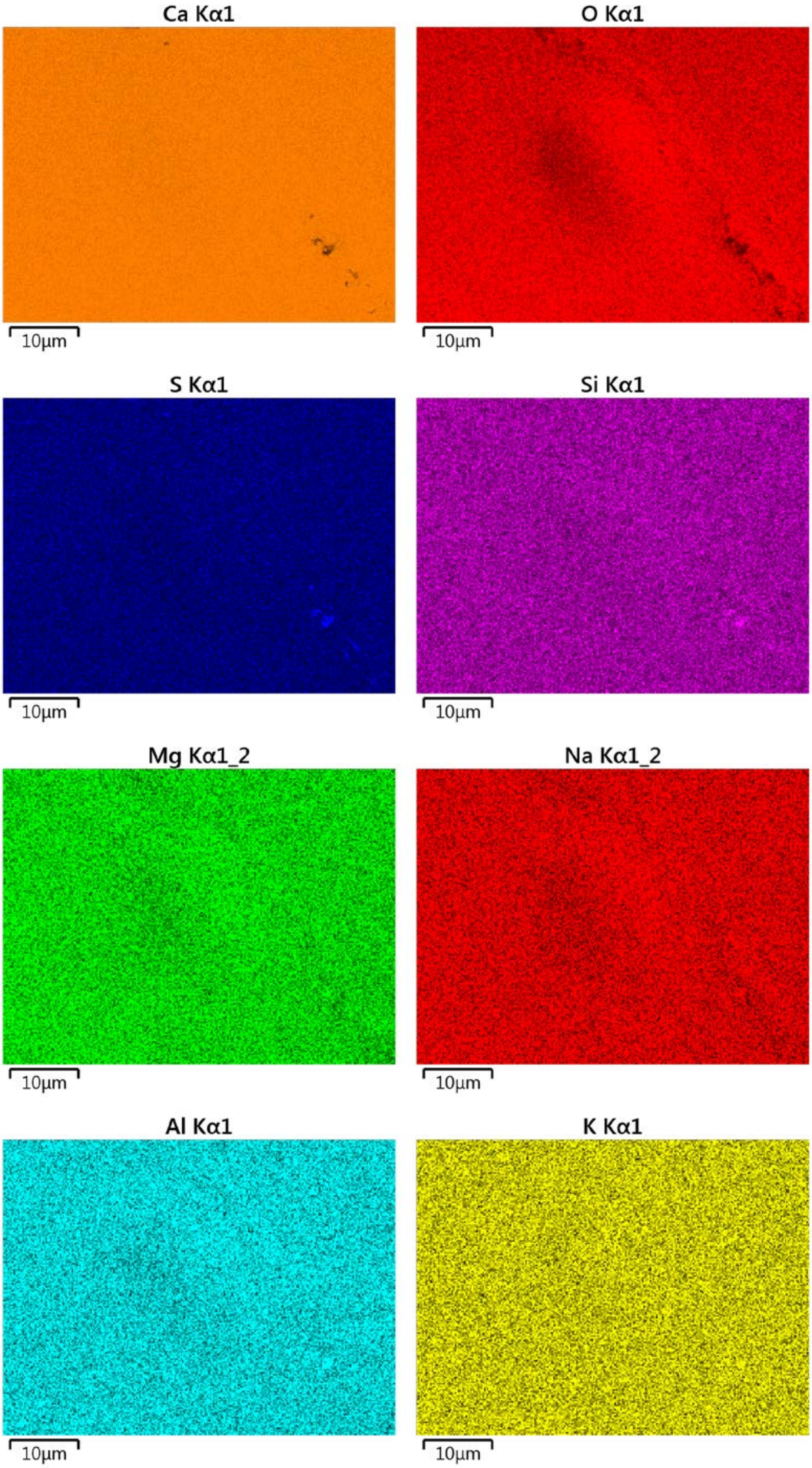
Ca Kα1
10μm
O Kα1
10μm
S Kα1
10μm
Si Kα1
10μm
Mg Kα1_2
10μm
Na Kα1_2
10μm
Al Kα1
10μm
K Kα1
10μm

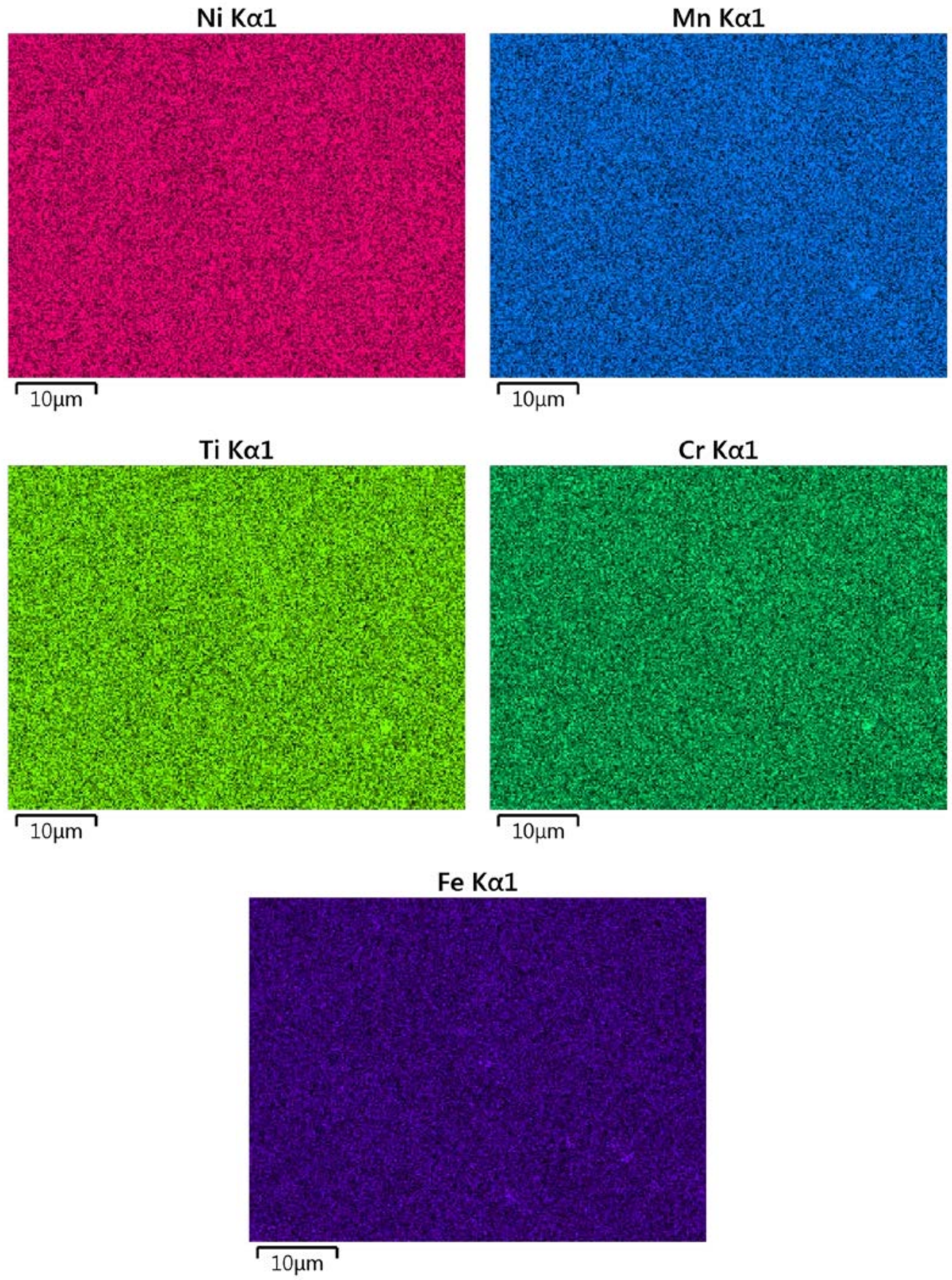
Ni Kα1
10μm
Mn Kα1
10μm
Ti Kα1
10μm
Cr Kα1
10μm
Fe Kα1
10μm

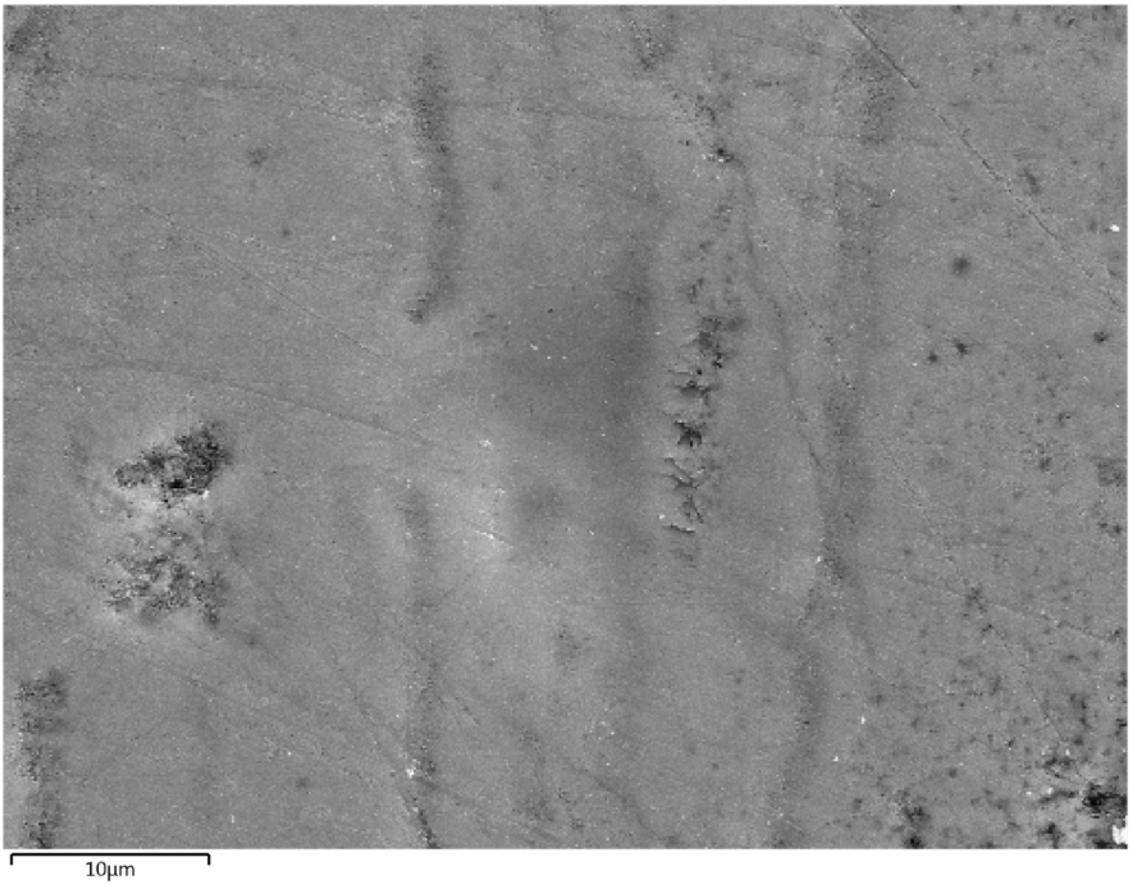

SEM image

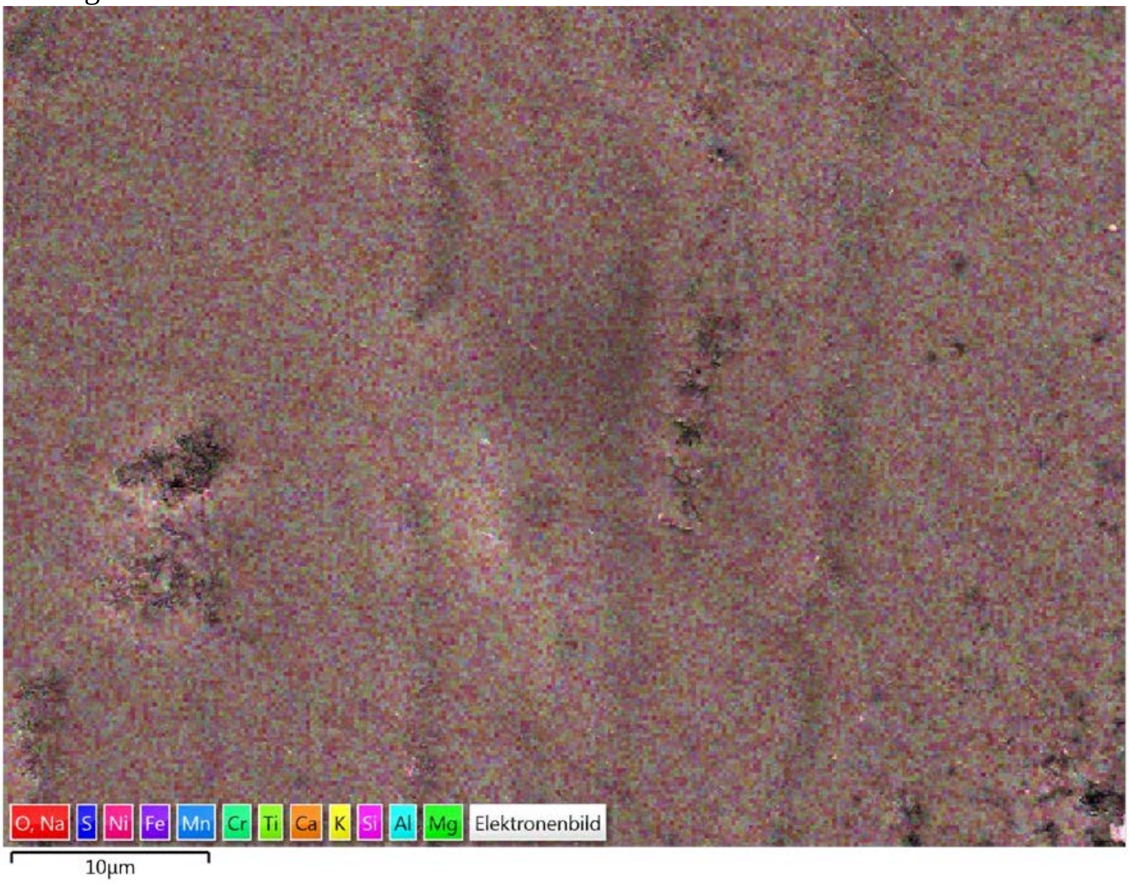

Overlay SEM-EDS image

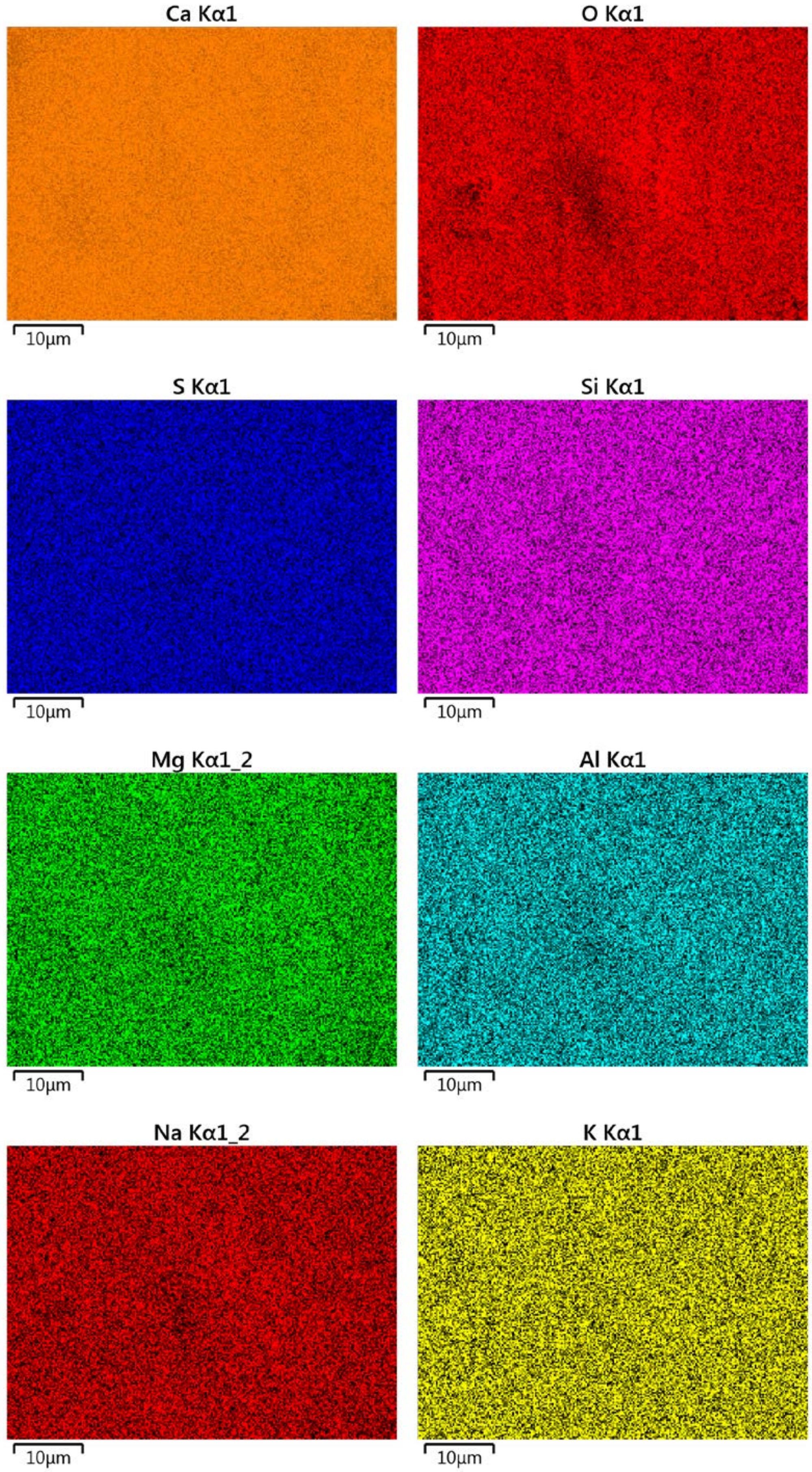
Ca Kα1
O Kα1
10μm
10μm
S Kα1
Si Kα1
10μm
10μm
Mg Kα1_2
Al Kα1
10μm
10μm
Na Kα1_2
K Kα1
10μm
10μm

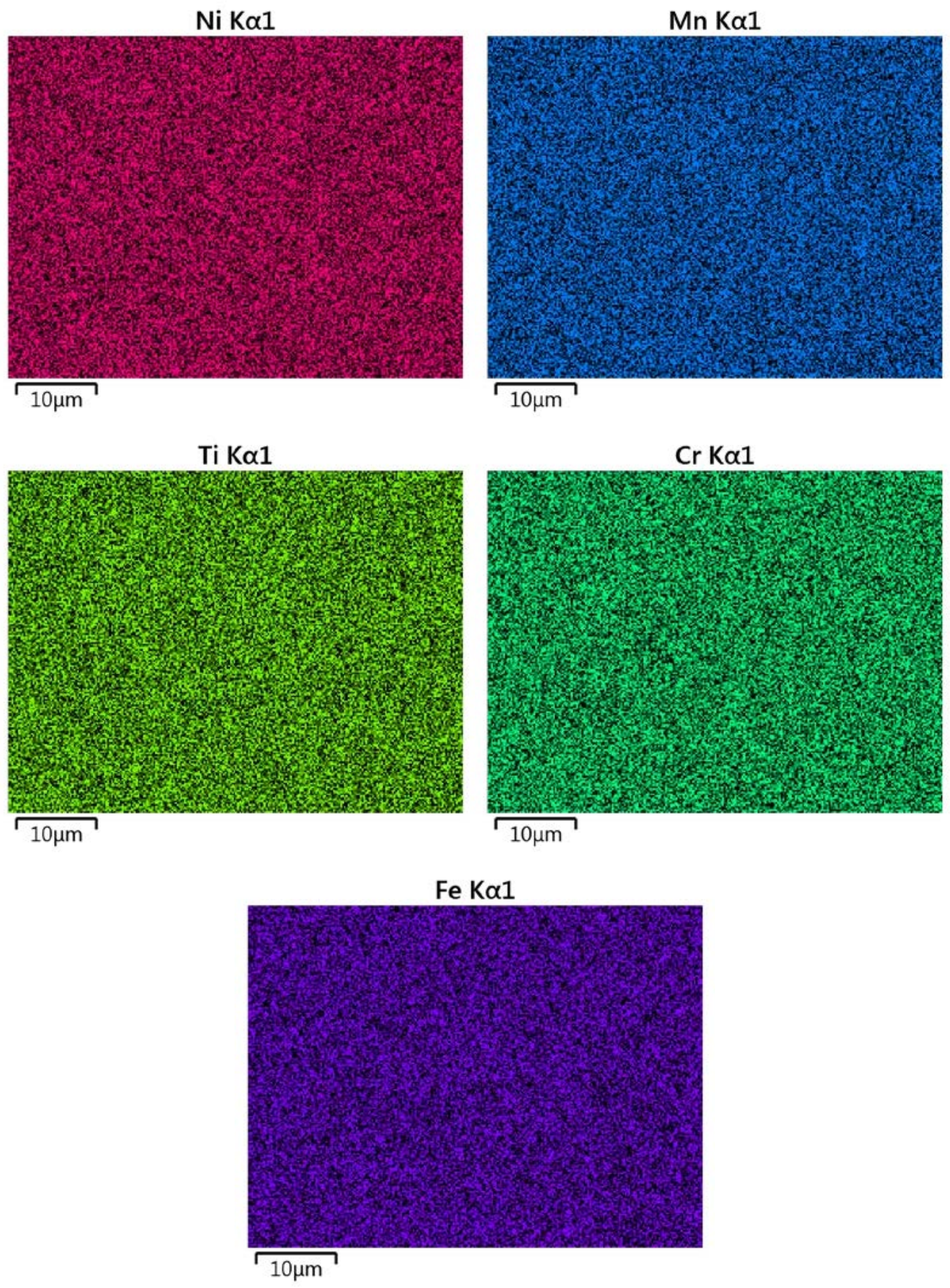
Ni Kα1
10μm
Mn Kα1
10μm
Ti Kα1
10μm
Cr Kα1
10μm
Fe Kα1
10μm

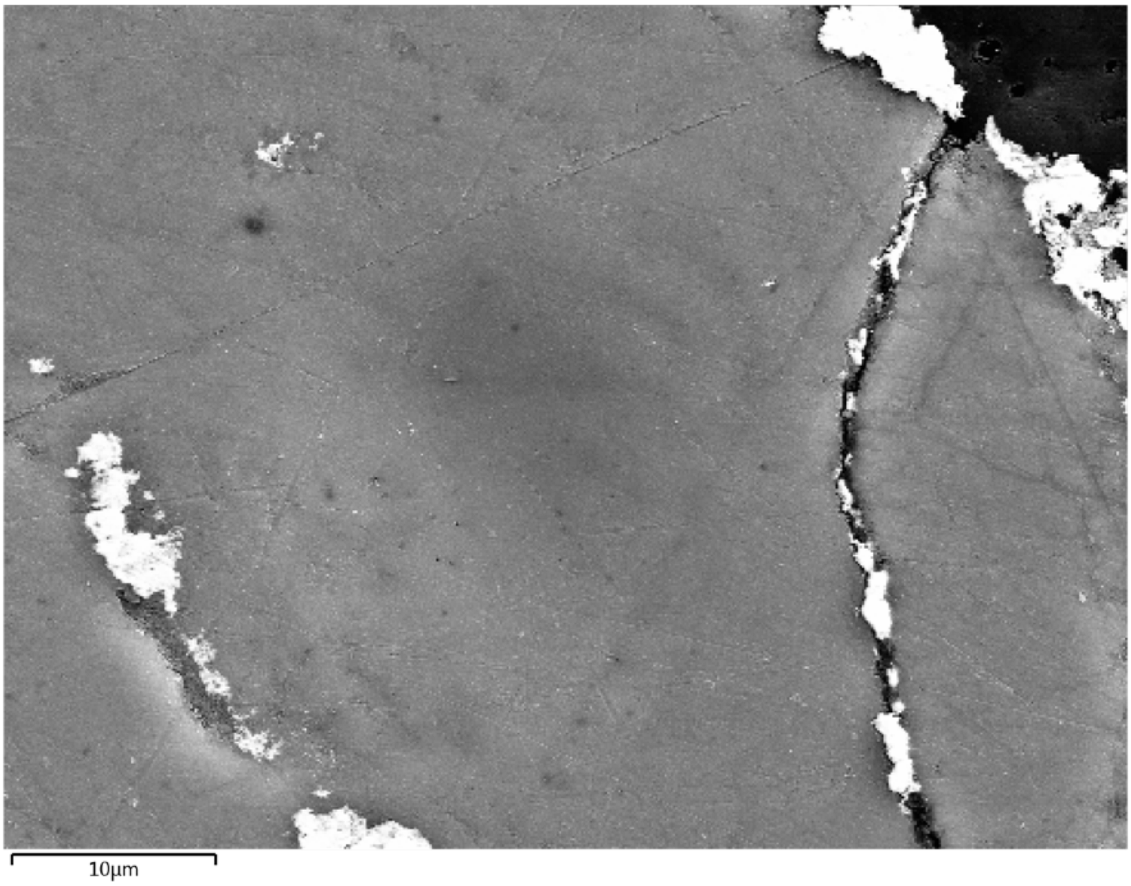

SEM image

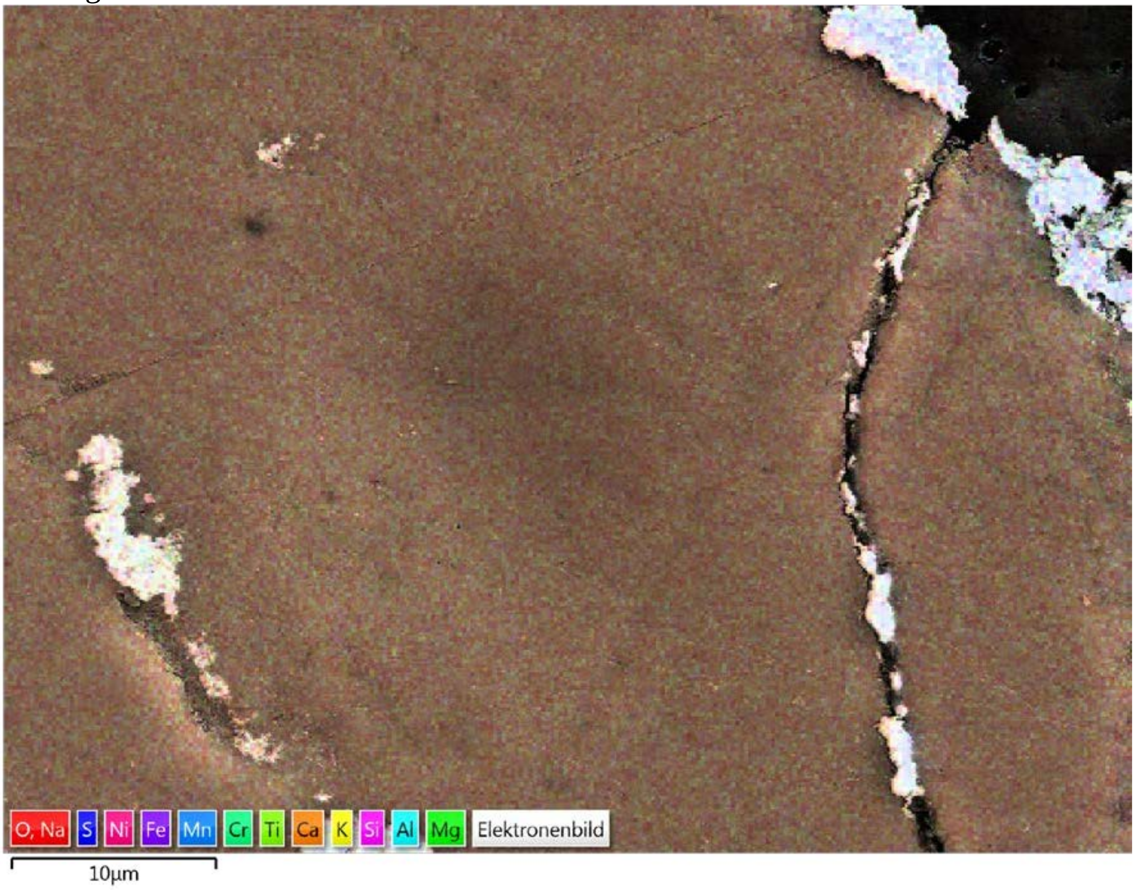

Overlay SEM-EDS image

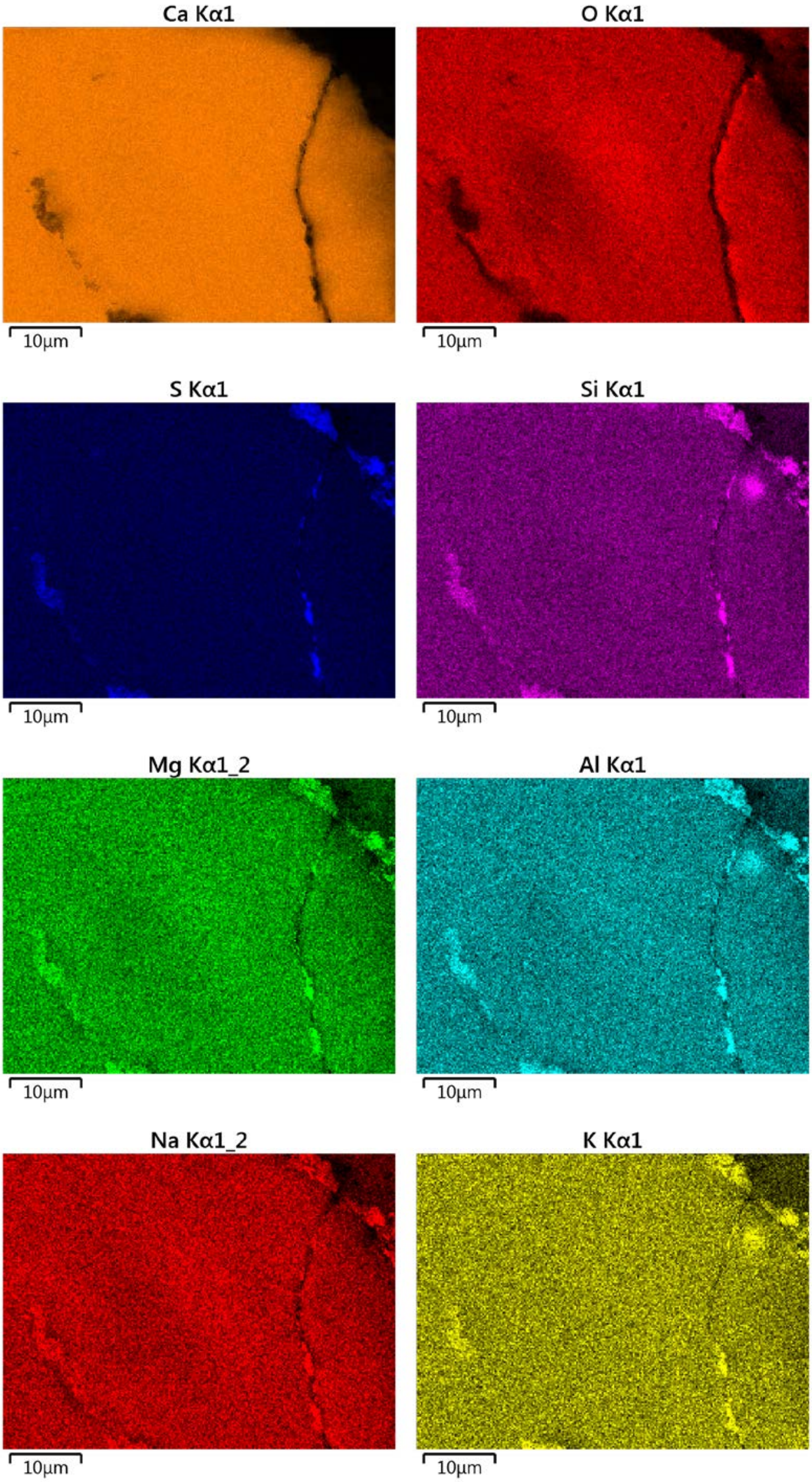
Ca Kα1
O Kα1
10μm
10μm
S Kα1
Si Kα1
10μm
10μm
Mg Kα1_2
Al Kα1
10μm
10μm
Na Kα1_2
K Kα1
10μm
10μm

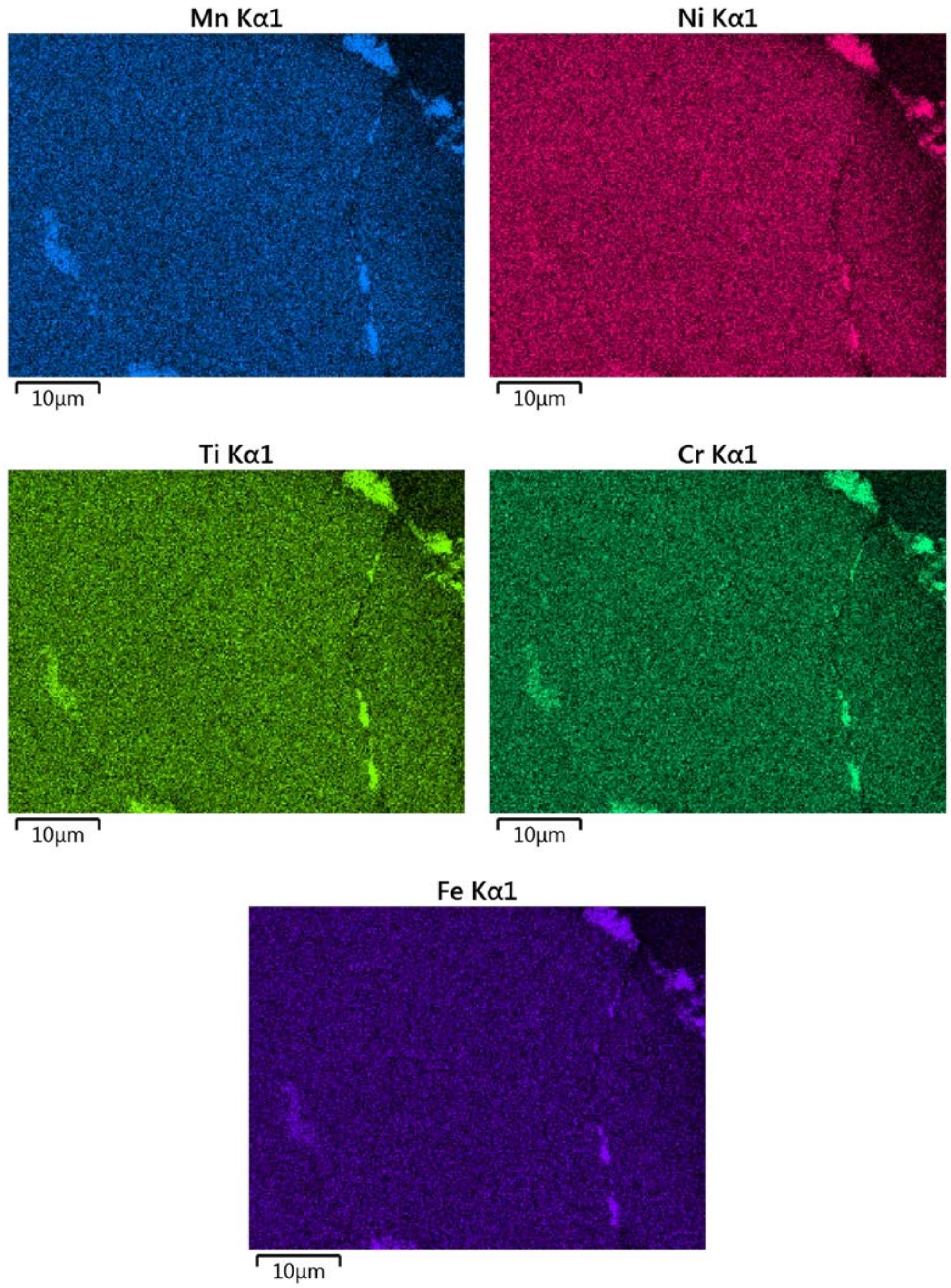
Mn Kα1
10μm
Ni Kα1
10μm
Ti Kα1
10μm
Cr Kα1
10μm
Fe Kα1
10μm
